# A Color‐Shifting Near‐Infrared Fluorescent Aptamer–Fluorophore Module for Live‐Cell RNA Imaging

**DOI:** 10.1002/anie.202107250

**Published:** 2021-08-20

**Authors:** Jingye Zhang, Lu Wang, Andres Jäschke, Murat Sunbul

**Affiliations:** ^1^ Institute of Pharmacy and Molecular Biotechnology (IPMB) Heidelberg University Im Neuenheimer Feld 364 69120 Heidelberg Germany; ^2^ Department of Chemical Biology Max Planck Institute for Medical Research Jahnstraße 29 69120 Heidelberg Germany

**Keywords:** fluorophores, ratiometric imaging, RNA aptamer, RNA imaging, SELEX

## Abstract

Fluorescent light‐up RNA aptamers (FLAPs) have become promising tools for visualizing RNAs in living cells. Specific binding of FLAPs to their non‐fluorescent cognate ligands results in a dramatic fluorescence increase, thereby allowing RNA imaging. Here, we present a color‐shifting aptamer‐fluorophore system, where the free dye is cyan fluorescent and the aptamer‐dye complex is near‐infrared (NIR) fluorescent. Unlike other reported FLAPs, this system enables ratiometric RNA imaging. To design the color‐shifting system, we synthesized a series of environmentally sensitive benzopyrylium‐coumarin hybrid fluorophores which exist in equilibrium between a cyan fluorescent spirocyclic form and a NIR fluorescent zwitterionic form. As an RNA tag, we evolved a 38‐nucleotide aptamer that selectively binds the zwitterionic forms with nanomolar affinity. We used this system as a light‐up RNA marker to image mRNAs in the NIR region and demonstrated its utility in ratiometric analysis of target RNAs expressed at different levels in single cells.

## Graphical abstract

A color‐shifting, near‐infrared (NIR) fluorescent aptamer–fluorophore module based on benzopyrylium‐coumarin (BC) fluorophores for ratiometric RNA imaging is reported. Free BC is cyan fluorescent, however its fluorescence emission shifts to NIR upon complexation with the BC‐binding aptamer (BeCA). This system enabled the visualization of mRNAs in the NIR region and the ratiometric analysis of target RNAs expressed at different levels in single cells.

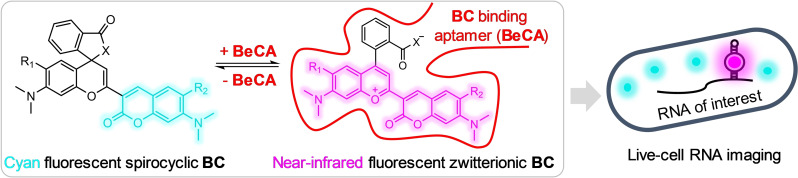

## Introduction

RNA labeling methods combined with advanced fluorescence microscopy enable imaging the complex dynamics of RNA in living cells with a high temporal and spatial resolution. The conventional methods to label RNA employ fluorophore‐labeled hybridization probes^[1]^ and molecular beacons,^[2]^ yet they suffer from impermeability and heterogeneous distribution of the probes.^[3]^ Fluorescent protein‐tagged RNA‐binding proteins (RBP) are still the current gold standard for live‐cell RNA imaging.^[4]^ However, these RBP‐based tagging systems including a recently developed CRISPR‐Cas system for endogenous RNA imaging^[5]^ have limitations such as high background fluorescence and potential alterations in RNA properties due to the bulky tag.^[3]^

The currently emerging field of fluorescent light‐up RNA aptamers (FLAPs) provides powerful tools for live‐cell RNA imaging. Non‐covalent binding of cell‐permeable, fluorogenic dyes to their cognate aptamers induces a fluorescence turn‐on, enabling RNA detection with high signal‐to‐background ratios. Among literature‐reported FLAP systems, the origin of dye fluorescence quenching can be based on: *i)* vibrational and rotational motions (e.g. MG aptamer,^[6]^ Spinach^[7]^ and its relatives,^[8]^ Pepper^[9]^); *ii)* ground‐state complex formation (e.g. SRB‐2,^[10]^ DNB,^[11]^ Riboglow,^[12]^ o‐Coral^[13]^ and RhoBAST^[14]^); *iii)* spirolactonization (e.g. SiRA^[15]^). SiRA was an important milestone towards in vivo RNA imaging because of the lack of photostable and bright FLAPs that fluoresce in the NIR region.

The fluorogenic nature of FLAPs enables imaging RNAs of interest (ROIs) in living cells in the presence of their cognate ligands. However, differences in the cellular uptake of the dye between different cells, heterogeneous probe distribution within a single cell, probe instability due to photobleaching, variations in the cell morphology such as cell thickness and focal plane during an imaging experiment could cause signal fluctuations and consequently misinterpretation of data.^[16]^ An effective way to solve this problem is to use a ratiometric system that enables imaging both the free dye and the RNA‐fluorophore complex simultaneously. Unfortunately, all FLAPs reported so far are based on a single‐color fluorescence turn‐on.

**Figure 1:**
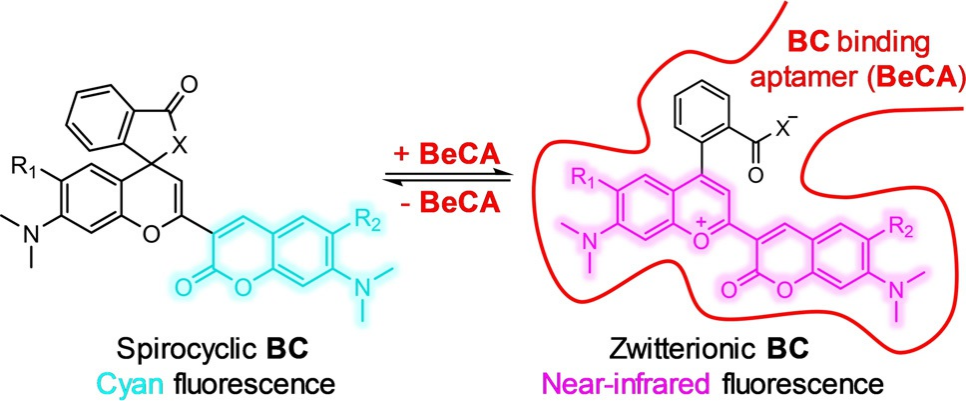
Design principle of color‐shifting, NIR fluorescent aptamer‐fluorophore module based on benzopyrylium‐coumarin (**BC**) fluorophores. Free **BC** is in the cyan fluorescent spirocyclic form, whereas the aptamer (**BeCA**)‐bound **BC** is in the NIR fluorescent zwitterionic form.

In this work, to address these issues, we report the evolution, characterization, and application of a novel color‐shifting NIR‐fluorescent aptamer‐fluorophore module based on spirolactamization of fluorophores for RNA imaging in living cells (Figure 1). The free fluorophore is cyan fluorescent in solution while the aptamer‐bound form fluoresces in the NIR region. We demonstrate the application of this color‐shifting FLAP system as a genetically encoded tag to visualize mRNAs in the NIR region using its light‐up feature as well as its capability for ratiometric analysis of RNA expression levels in single cells.

## Results and Discussion

**Design of a Color‐Shifting Aptamer‐Fluorophore Module**. Rhodamine dyes have been widely used for biological imaging owing to their high quantum yield, excellent photostability, and good cell permeability.[^13^, ^15^, ^17^] Furthermore, the rational design of new fluorescent probes that modulate the spirocyclization of rhodamine dyes is becoming a powerful method for background‐free fluorescence microscopy in vivo.^[18]^ The spirocyclic (lactone or lactam) form of rhodamine is colorless and non‐fluorescent due to the disruption of the conjugated π‐system, whereas the open form is colored and fluorescent due to the extended π‐conjugation. This fluorescence switching mechanism has also been applied to NIR‐fluorescent silicon rhodamines, enabling precise modulation of the ring‐open and ‐closed forms.[^18^, ^19^] However, these NIR fluorescent rhodamine scaffolds can only be used for fluorescence turn‐on probes.

Here, we aimed to design a color‐shifting aptamer‐fluorophore module that enables simultaneous imaging of the RNA‐fluorophore complex and the free fluorophore. Moreover, it is desirable to have the emission of the complex in the NIR region due to its better live cell compatibility. To this end, we chose an environmentally sensitive hybrid fluorophore consisting of benzopyrylium and coumarin moieties (**BC**) as the core structure of our probes. Similar to rhodamine dyes, **BC** fluorophores exist in an equilibrium between a spirocyclic and a zwitterionic form, however the closed form is cyan‐ and the open form is NIR‐fluorescent.^[20]^ We envisioned that the color‐switching property of **BC** fluorophores could be exploited for ratiometric RNA imaging provided that a high‐affinity aptamer binding exclusively to the open form can be generated. Moreover, the fluorescence light‐up feature of the aptamer can be used for imaging RNAs in the NIR region. The ideal color‐shifting **BC** compound should exist mainly as a cyan fluorescent spirocyclic form in the unbound state; however, it should efficiently switch to the NIR fluorescent zwitterionic form upon binding to the aptamer.

In order to increase the chances to evolve an aptamer binding preferentially to the NIR fluorescent open form, we planned to use a **BC** fluorophore that mainly exists in the zwitterionic form in aqueous solution at physiological pH. This fluorophore was immobilized on a solid support as bait during the in vitro evolution of aptamers. After the discovery of the aptamer, the structure of the **BC** scaffold was minimally modified in order to fine tune the open‐closed ratio. Finally, the color‐shifting efficiency of the aptamer upon binding to a series of **BC** analogs was investigated to find the best aptamer‐fluorophore pair for ratiometric RNA imaging.

**Figure 2:**
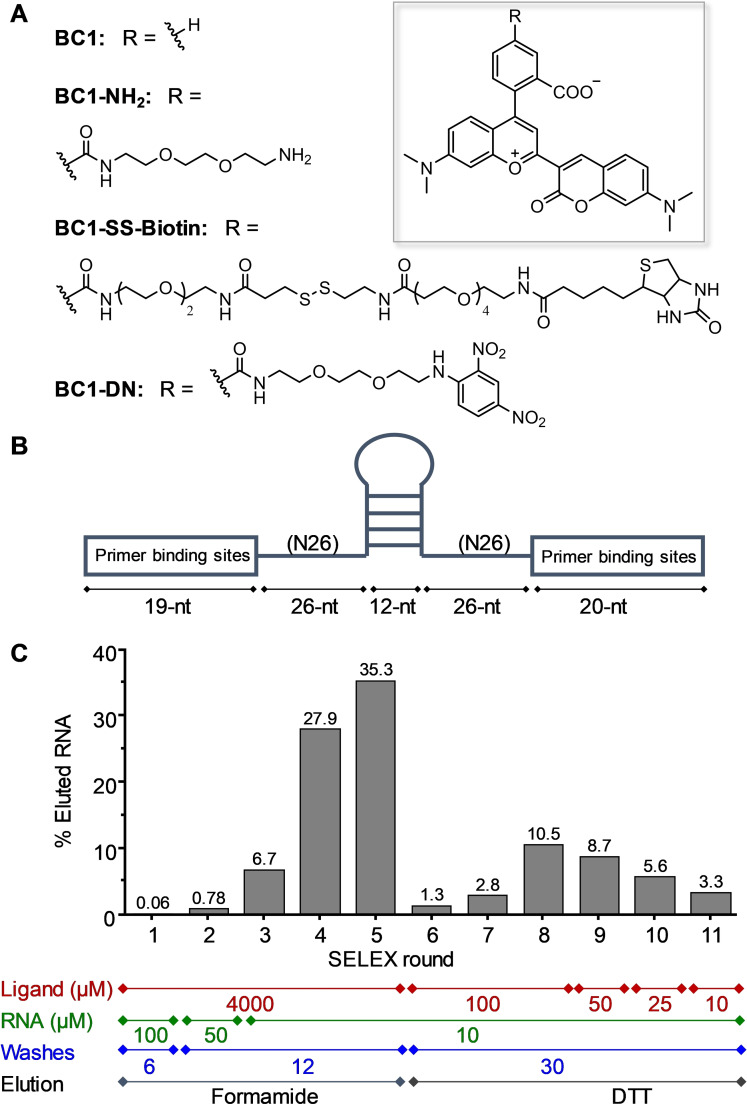
In vitro selection of **BC**‐binding aptamers. A) Chemical structures of **BC1**, **BC1‐NH_2_**, **BC1‐SS‐Biotin** and **BC1‐DN**. B) Design of the partially structured RNA library. C) Progress of the in vitro selection monitored by measuring the fraction of RNA eluted from the **BC1**‐conjugated beads.

**In vitro Evolution of Aptamers for BC1**. We performed SELEX (Systematic Evolution of Ligands by EXponential enrichment) to find RNA aptamers binding specifically to the **BC** zwitterion.^[21]^ **BC1** was chosen as the ligand for in vitro selection because it predominantly exists in the NIR fluorescent zwitterionic form^[20b]^ (Figure 2 A). To attach ligands to a solid support for SELEX, amine‐functionalized **BC1** (**BC1‐NH_2_**, Figure 2 A) was synthesized and immobilized on N‐Hydroxysuccinimide‐activated sepharose beads (Scheme S1).[^15^, ^20b^]

We started the selection with an RNA library containing ≈3×10^15^ different sequences. The sequence of the RNA library consisted of a 5′‐forward primer binding site, a 26‐nucleotide (nt) random region, a 12‐nt constant sequence forming a stable stem‐loop, another 26‐nt random region and a 3′‐reverse primer binding site^[22]^ (Figure 2 B). RNA transcripts were first incubated with a mock resin to remove sepharose‐binding RNA sequences, and then with sepharose beads functionalized with **BC1** (≈4 mM on the resin). After washing the beads, bound RNA was nonspecifically eluted with formamide solution, precipitated, reverse‐transcribed and PCR‐amplified. The obtained DNA was in vitro transcribed and the enriched RNA pool served as an input for the next round of selection (Figure S1). After 5 rounds of SELEX, the fraction of eluted RNA increased to 35 %, indicating the successful selection of **BC1**‐binding aptamers (Figure 2 C). In order to select for high affinity binders, we increased the selection pressure by gradually decreasing the ligand concentration on the beads. For this purpose, we synthesized a biotinylated **BC1** ligand containing a linker with a disulfide bond (**BC1‐SS‐Biotin**, Figure 2 A) and used streptavidin‐conjugated beads as solid supports (Scheme S1). This way, the concentration of **BC1‐SS‐Biotin** on streptavidin‐beads could be precisely and easily controlled and decreased during the course of SELEX. Furthermore, bound RNA could be specifically eluted with DTT by reducing the disulfide bond between the **BC1** and biotin. Selection stringency was increased gradually by decreasing the ligand concentration from 4 mM to 10 μM, lowering RNA input concentration from 100 to 10 μM, increasing the washes from 6 to 30 column volumes, and eluting resin‐bound RNA with DTT (Figure 2 C). After eleven iterative rounds, the eluted RNA pool was reverse‐transcribed, PCR‐amplified, cloned into plasmids that were subsequently transformed into bacteria. Single colonies of bacteria were picked and the plasmids were sequenced by Sanger sequencing.

**Identification of a High Affinity BC1‐Binding Aptamer**. After analyzing 60 individual colonies, we identified 52 unique RNA sequences (Figure S2), all of which were in vitro transcribed for screening according to their affinity towards **BC1**. Since **BC1** is predominantly in the NIR‐fluorescent zwitterionic form, binding of an aptamer to **BC1** would not necessarily cause a fluorescence or spectral change, making **BC1** impractical for aptamer screening. To solve this problem, we synthesized a fluorescence turn‐on probe of **BC1** by attaching the contact quencher dinitroaniline (**DN**) to yield **BC1‐DN** (Figure 2 A and Scheme S2). **DN** effectively quenches the fluorescence of **BC1** by forming an intramolecular ground state heterodimer.^[10]^ In the presence of a **BC1**‐binding aptamer, the fluorophore would interact with the aptamer rather than the quencher, thereby enhance the fluorescence. All RNA transcripts were screened based on the fluorescence intensity enhancement of **BC1‐DN** (100 nM) upon mixing with aptamers (10 μM) (Figure S2). Out of 52 sequences, 8 were determined to be highly active showing >6‐fold fluorescence enhancement, 29 were moderately active (3‐ to 6‐fold), and 15 were less active (<3‐fold). After measuring dissociation constants (*K*_D_) between **BC1‐DN** and the highly active sequences (Figure S3), we discovered that RNA8 showed the best binding affinity (*K*_D_=920 nΜ) and 10‐fold fluorescence enhancement; therefore, it was selected for further investigation.

**Figure 3:**
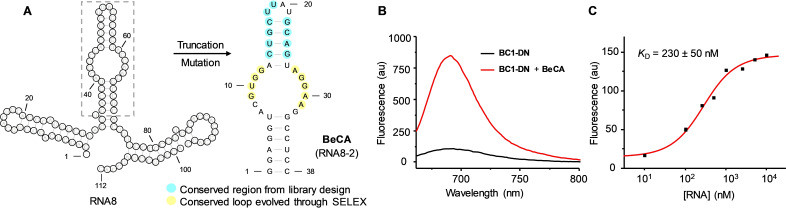
Truncation studies of **BC1**‐binding RNA aptamers. A) Predicted secondary structure of RNA8 and the minimal **BC1**‐binding aptamer **BeCA**. The conserved nucleotides are highlighted in blue or yellow depending on their origin. B) Fluorescence emission spectra of **BC1‐DN** (100 nM) in the presence or absence of **BeCA** (10 μM). C) *K*_D_ curve of the **BeCA**‐**BC1‐DN** complex. Fluorescence intensities were recorded after incubating **BC1‐DN** (100 nM) with different concentrations of the **BeCA** aptamer (0—25 μM). For the fluorescence measurements in panels (B) and (C), an excitation of 652±5 nm and a buffer containing 20 mM Hepes (pH 7.4), 5 mM MgCl_2_, and 125 mM KCl were used.

Truncation of RNA8 and the stabilization of the terminal helix by inserting an extra G‐C pair yielded a minimal 38‐nt RNA8‐2 aptamer, dubbed as **BeCA** (Figure 3 A and Figure S4). **BeCA** binds **BC1‐DN** with a *K*_D_ of 230 nM, and displays a 9.7‐fold fluorescence turn‐on (Figure 3 B,C). Further truncations or deletion of the internal hairpin structure caused a significant loss of fluorogenicity (Figure S4). Sequence alignment and secondary structure analysis of the aptamers with the highest turn‐on values revealed that sequences GUGG and AGGAA in the main loop are conserved (Figure 3 A, Figure S5A). Additional mutational studies did not considerably improve the affinity and fluorescence turn‐on of **BeCA** (Figure S5B).

**Figure 4:**
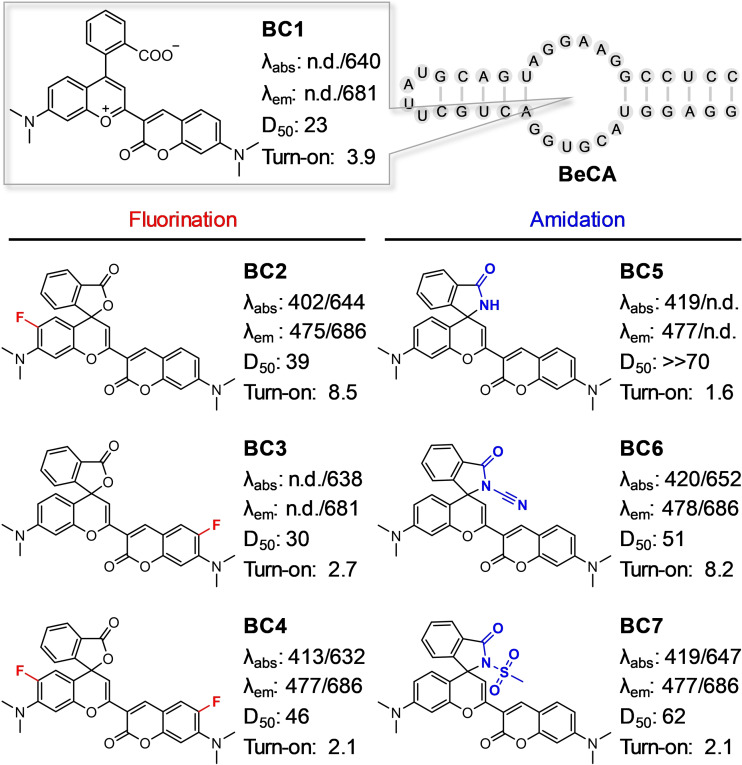
Chemical structures of **BC 1**–**7** and their fluorescence properties. *λ*_abs_ and *λ*_em_ represent the maximum absorption and emission wavelength (nm) of the spirocyclic/zwitterionic forms, respectively, in 20 mM Hepes (pH 7.4) containing 0.1 % Triton X‐100. n.d. represents not detected. D_50_ represented the dielectric constants at which half of the maximum absorbance of zwitterions were observed in dioxane/water mixture (v/v: 10/90–90/10). Turn‐on factors were determined by dividing maximal NIR fluorescence intensities of **BC** fluorophores (100 nM) in the presence of **BeCA** (10 μM) by those of **BC** fluorophores (100 nM). For the fluorescence measurements, the **BC** fluorophores were excited at their excitation maxima (±5 nm) in a buffer containing 20 mM Hepes (pH 7.4), 5 mM MgCl_2_, and 125 mM KCl.

**Fine‐Tuning Spirocyclization of BC**. The **BeCA** aptamer was designed and evolved to selectively bind **BC1**, which mainly exists in the zwitterionic form. To obtain a color‐shifting ligand for **BeCA**, we aimed to increase the propensity of **BC1** to form the corresponding spirocyclic form in the unbound state by chemically altering its structure. However, these modifications should be minimal so that the specific interactions between **BeCA** and **BC1** are not negatively affected. For this purpose, we took two different approaches. The first approach was based on the introduction of electron‐withdrawing fluorine atoms to the aromatic groups of benzopyrylium and coumarin (Figure 4). Decreasing the electron density in the π‐conjugated system allows more efficient nucleophilic attack by the carboxyl group to form the spirolactone.^[23]^ The second approach was, however, based on increasing the nucleophilicity of the carboxyl group without modifying benzopyrylium and coumarin. The conversion of the carboxyl group to electron‐deficient amides has recently been reported to shift rhodamines’ equilibrium to the spirocyclic configuration.[^18a^, ^20d^] Hence, electron‐withdrawing fluorine atoms (**BC 2**–**4**) and electron‐deficient amides (**BC 5**–**7**) were introduced to the **BC1** scaffold to yield a series of **BC** analogs (Figure 4 and Scheme S3). Probes **BC 1**–**7** were synthesized and characterized using literature‐reported routes[^18a^, ^20a^, ^23b^] (Supporting Information).

We then evaluated how fluorine or amide substitutions can modulate the equilibrium between spirocylic and zwitterionic forms by examining the absorbance of **BC 1**–**7** as a function of the dielectric constant. Using water‐dioxane mixtures, we determined the D_50_ values, the dielectric constant at which half of the maximum absorbance of zwitterion was observed (Figure 4 and Figure S6). Water promotes the formation of the zwitterionic form due to its high dielectric constant (*ϵ*_r_=80.4), whereas dioxane with a low dielectric constant (*ϵ*_r_=2.2) favors the spirocyclic form. Rhodamine‐based fluorophores with D_50_ values around 50 have been shown to be suitable candidates for the generation of fluorogenic probes for cellular imaging.^[18a]^ **BC1** showed a D_50_ value of 23, indicating that it exists primarily in the zwitterionic form in aqueous solution. Addition of an electron‐withdrawing fluorine atom to coumarin (**BC3**) and to benzopyrylium (**BC2**) increased the D_50_ values to 30 and 39, respectively. This result confirmed that fluorine substitutions successfully shift the equilibrium towards closed form in aqueous solution. Furthermore, introduction of two fluorine atoms (**BC4**), one for each coumarin and benzopyrylium, elevated the D_50_ value to 46, as expected.

Exchanging the carboxylic acid group with an amide group (**BC5**) yielded a fluorophore with dramatically increased D_50_ (≫70), implying that **BC5** is almost completely in the cyan fluorescent spirolactam form in water. However, the **BC** derivatives carrying cyanamide (**BC6**) and methylsulfonamide (**BC7**) have D_50_ values of 51 and 62, respectively, lower than that of **BC5** due to the decreased nucleophilicity of electron‐deficient amides. Yet, both **BC6** and **BC7** displayed much higher D_50_ values than **BC1**.

Overall, minimal chemical modifications on the **BC1** scaffold enabled fine‐tuning of the equilibrium between spirocyclic and zwitterionic forms as demonstrated by the increased D_50_ values, ranging from 23 to >70. As a result, fluorophores **BC 4**–**7** hold potential to function as color‐shifting ligands for the **BeCA** aptamer in imaging applications.

**Characterization of a Color‐Shifting Aptamer‐Fluorophore Module**. The ideal color‐shifting **BC** fluorophore for **BeCA** should be cyan fluorescent in solution but emit in the NIR region upon binding to the aptamer. Therefore, the binding of **BeCA** to the ideal fluorophore would decrease the cyan fluorescence, increase the NIR fluorescence, and thereby increase the NIR/cyan ratio. First, we evaluated whether the chemically modified **BC 4**–**7** analogs bind **BeCA** and cause a fluorescence increase in NIR emission and a decrease in cyan fluorescence (Figure 4). Among these dyes, **BC6** showed the highest fluorescence enhancement of 8.2‐fold upon aptamer binding. As indicated by the D_50_ value, **BC6** displayed its maximum absorbance in the cyan region and existed mainly in the spirocyclic form (Figure S6). Therefore, **BC6** was chosen as the color‐shifting ligand for **BeCA**. Furthermore, the cyan and NIR fluorescence intensities of **BC6** did not significantly change in the presence of total RNA, supporting the specific interaction between **BC6** and **BeCA** (Figure S7).

**Figure 5:**
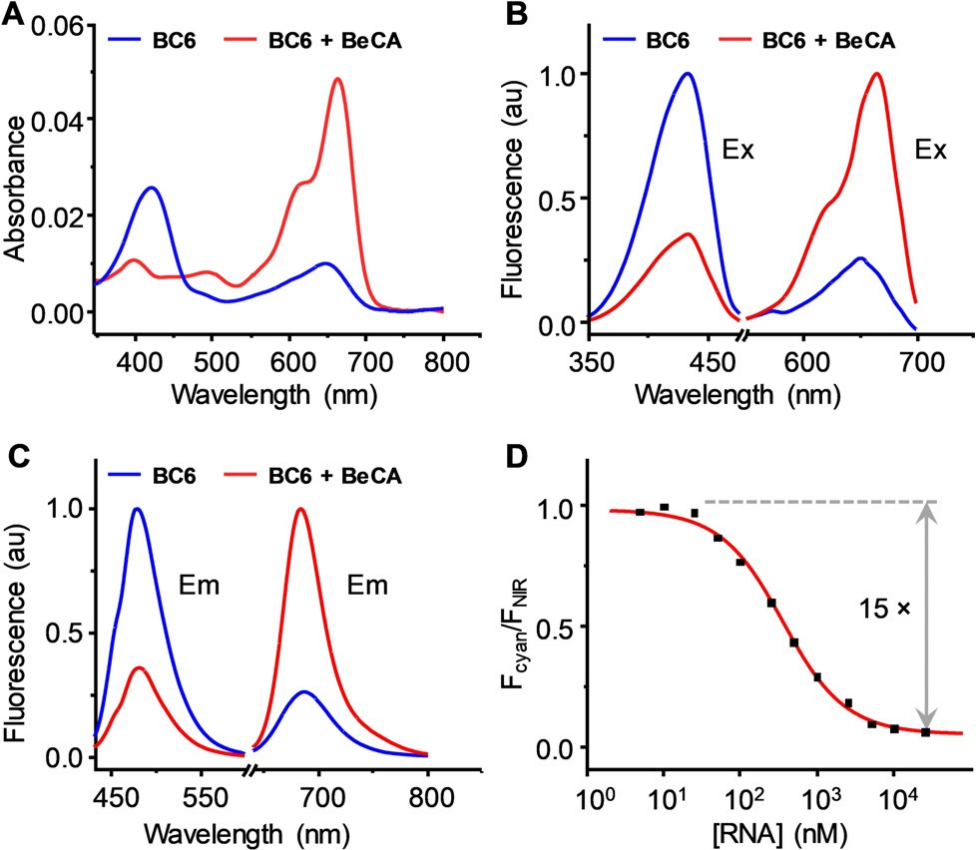
Photophysical characterization of the **BeCA**‐**BC6** complex. Absorption (A), excitation (B), and emission (C) spectra of **BC6** (1 μM) in the presence and absence of **BeCA** (25 μM). D) Plot of the emission ratio (*F*_cyan_/*F*_NIR_) of **BC6** (100 nM) as a function of **BeCA** concentration (0–25 μM). The spectra were measured in a buffer containing 20 mM Hepes (pH 7.4), 5 mM MgCl_2_, 125 mM KCl, and 0.05 % Triton X‐100.

Next, we examined the photophysical features of **BC6** in the presence of **BeCA** (Figure 5 and Table S1). Titration of **BC6** with the **BeCA** aptamer led to a decrease in the absorbance peak of the spirocyclic form (*λ*=420 nm), while an increase in the absorbance peak of the zwitterionic form (*λ*=665 nm), indicating the propensity of **BC6** to favor the zwitterionic form when bound to **BeCA**. Consistent with the absorbance change, the cyan fluorescence (*λ*=478 nm) dropped while the NIR fluorescence (*λ*=684 nm) increased with increasing concentration of **BeCA**. The color‐shifting process of **BC6**, from unbound to **BeCA**‐bound state, produced a dynamic range (cyan/NIR emission ratio change) as high as 15‐fold, substantially higher than the single‐wavelength NIR fluorescence turn‐on (Figure 5).

**BeCA** binds the ligand **BC6** with a *K*_D_ of 220 nM (Figure S8A), displaying essentially the same affinity as **BC1‐DN**. Notably, the fluorescence of **BeCA‐BC6** is independent of magnesium and potassium ions: 90 % of the maximum fluorescence was retained even in the absence of either of these cations, suggesting that the folding of **BeCA** and its complexation with **BC1** are independent of magnesium and potassium (Figure S8B,C). An increase in temperature from 25 to 37 °C caused a decrease in the fluorescence of **BeCA‐BC6** by only 25 %, comparable to other literature‐reported, thermally stable aptamer systems[^8a^, ^24^] (Figure S8D).

**Figure 6:**
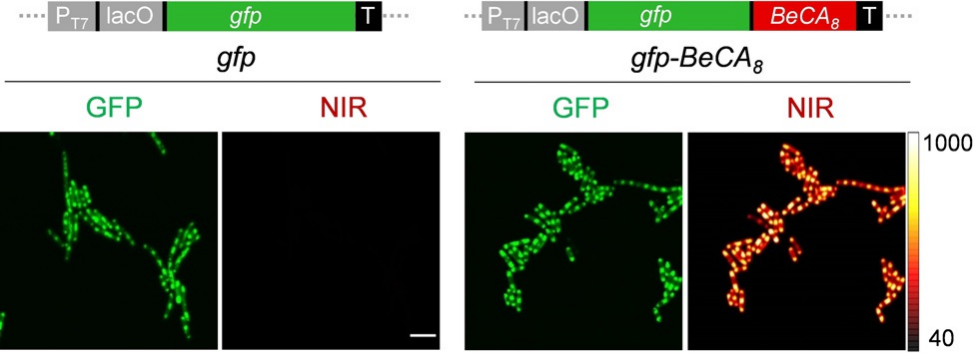
Confocal images of *gfp* mRNA tagged with **BeCA** in live bacteria. *E. coli* bacteria were transformed with either *gfp‐BeCA_8_* or *gfp* plasmids and mRNA expression was induced by the addition of IPTG. The cells were incubated with **BC6** (200 nM) for 15 min and imaged. Images in GFP and NIR channels were acquired using 488 nm and 640 nm lasers. Scale bar, 5 μm.

**BeCA as a NIR Light‐up Aptamer for RNA Imaging in Live Cells**. First, we analyzed the utility of **BeCA**‐**BC6** as a NIR fluorescence turn‐on tag for imaging mRNAs, which generally possess short half‐lives, complex structures and have low abundance. To this end, eight synonymous copies of **BeCA** without any stabilizing scaffold were introduced into the 3′‐untranslated region of the green fluorescent protein (*gfp*) gene. *Escherichia coli* (*E. coli*) expressing either *gfp* (control) or *gfp‐BeCA_8_* mRNA were imaged in the presence of **BC6** (Figure 6 and Figure S9). The bright NIR fluorescence emission was detected predominantly at the poles of bacteria expressing *gfp‐BeCA_8_* mRNA, but not in the control cells. The accumulation of mRNA at the bacterial poles demonstrated by the NIR signal was likely due to localization of the pET plasmid where mRNA transcription initiates.[^24^, ^25^] The average NIR fluorescence intensity at the bacterial poles of *gfp‐BeCA_8_*‐expressing cells was 6‐fold higher than that of the control bacteria when 200 nM of **BC6** was used. Furthermore, bacteria expressing *gfp* or *gfp‐BeCA_8_* displayed similar GFP fluorescence intensities, suggesting that the **BeCA** tag did not significantly affect the transcription and translation processes.

**BeCA** was also expressed as synonymous repeats in mammalian cells and successfully visualized using the fluorophore **BC6** in HEK293T cells (Figure S10). This experiment demonstrated that **BeCA** folds correctly in mammalian cells and could be used to image other target RNAs utilizing its light‐up feature and NIR fluorescence.

**BeCA for Ratiometric RNA imaging in Live Bacteria**. To investigate whether the **BeCA**‐**BC6** color‐shifting aptamer‐fluorophore module could be used for ratiometric imaging of RNAs, proof‐of‐principle live‐cell imaging experiments were performed. We expressed **BeCA** embedded in a tRNA scaffold in *E. coli* and imaged bacteria in the presence of **BC6** (500 nM) (Figure S11). **BeCA**‐expressing cells showed a much higher fluorescence signal in the NIR channel than the tRNA‐expressing control cells, indicating that **BeCA** selectively binds the **BC6** zwitterion and lights up in the NIR channel. On the other hand, bacteria, whether expressing **BeCA** or not, showed similar cyan fluorescence due to the dynamic equilibrium between the intracellular and extracellular **BC6**. Increasing the concentration of **BC6** from 500 nM to 1 μM in bacterial imaging did not significantly improve the NIR signal in bacteria expressing **BeCA**, yet it resulted in elevated NIR background fluorescence in control bacteria. As expected, using a higher concentration of **BC6** increased the cyan fluorescence in both **BeCA**‐expressing and control bacteria. Even in the presence of as low as 200 nM of **BC6**, both cyan and NIR channel images of **BeCA**‐expressing cells exhibited excellent image quality.

Next, we tested if we can detect variations in cyan and NIR emission intensities of **BeCA‐BC6** upon environmental changes such as exposure to high salt concentration. Upon addition of ammonium acetate to the **BeCA‐BC6** complex in vitro, which can replace mono and divalent cations in the aptamer, we observed an increase in the cyan fluorescence (1.7‐fold) and decrease in both NIR fluorescence (4.1‐fold) and the ratio of NIR/cyan fluorescence (6.8‐fold) intensities. This can be explained by unfolding of the aptamer followed by the dissociation of the dye from the aptamer (Figure S12A). Motivated by this result, we performed a similar experiment with **BeCA**‐expressing live bacteria that had already been incubated with **BC6**. After a rapid treatment of the bacteria with ammonium acetate, confocal images indicated a fluorescence increase in the cyan channel and a decrease in both NIR and ratiometric (NIR/cyan) channels as anticipated (Figure S12B,C). Ratiometric images were obtained by pixel‐to‐pixel division of the fluorescence intensities in the NIR channel by the cyan channel. This experiment allowed us to visualize the unfolding of the **BeCA** aptamer in live bacteria exploiting the ratiometric features of **BeCA‐BC6**.

**Figure 7:**
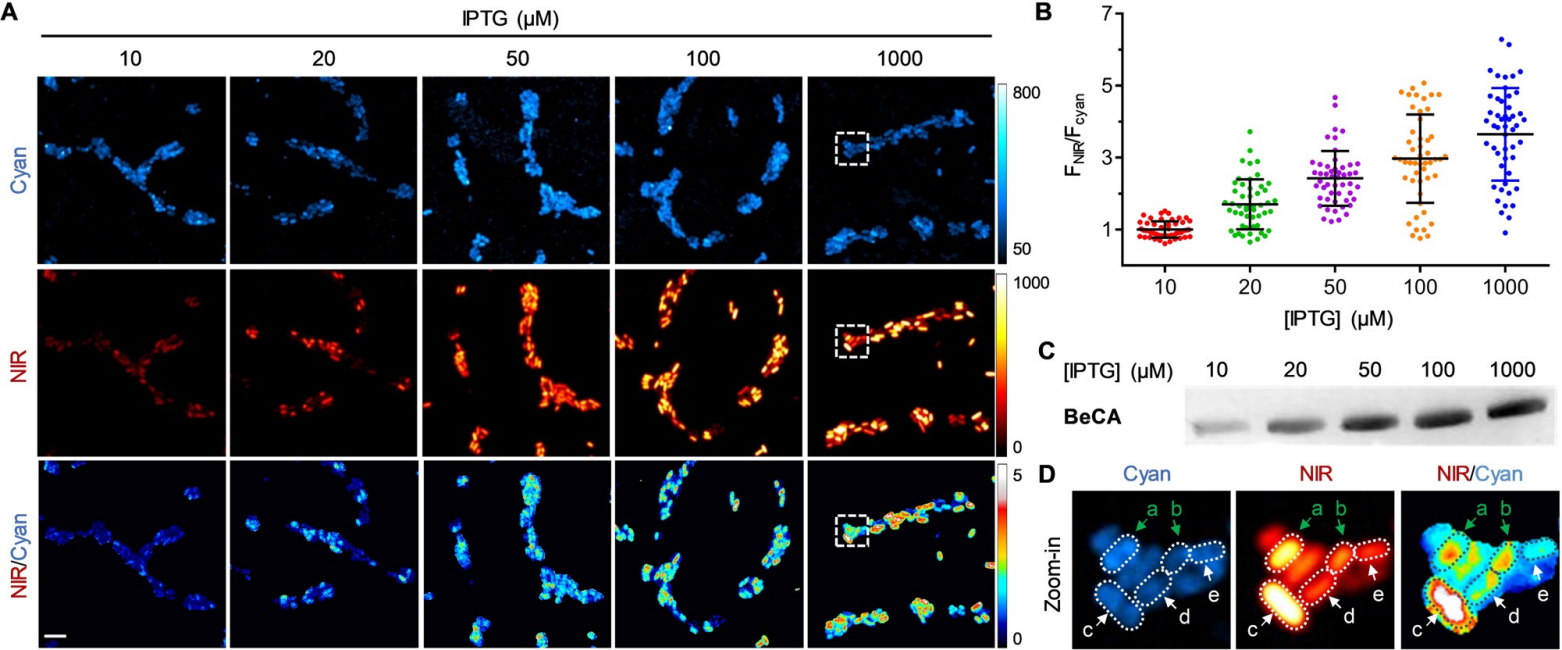
Confocal images of live *E. coli* expressing different levels of **BeCA**. A) Bacteria were transformed with plasmids expressing **BeCA** and the transcription was induced by addition of IPTG at varying concentrations (10–1000 μM). Then, bacteria were incubated with **BC6** (200 nM, 15 min) and imaged in M9 medium containing 2 mM of MgCl_2_. Images in cyan and NIR channels were acquired using 405 nm and 640 nm lasers, respectively. Scale bar: 5 μm. B) Quantification of the mean fluorescence ratio (NIR/cyan) of bacteria expressing **BeCA** at different IPTG concentrations (10–1000 μM). Each dot represents a single cell. Means ± standard deviation (s.d.) are shown (*n*=50 cells from five independent images). Mean of the NIR/cyan ratio for bacteria treated with 10 μM was normalized to 1. C) Denaturing polyacrylamide gel analysis of **BeCA** in total RNAs isolated from bacteria treated with various IPTG concentrations (10–1000 μM). D) Zoom‐in images of the **BeCA**‐expressing cells depicted in panel A. Green arrows indicate the cells **a** and **b**, of which **BeCA** concentration was corrected using ratiometric analysis. White arrows show transcription heterogeneity at the single‐cell level. Transcription efficiency analysis according to the ratiometric images: cell **c** > cell **d** > cell **e**.

We also used the color‐shifting capability of **BeCA**‐**BC6** for the analysis of RNA expression levels in living cells. The transcription of **BeCA** can be effectively activated by isopropyl‐β‐d‐thiogalactopyranoside (IPTG).^[26]^ Cells expressing **BeCA** were treated with varying concentrations of IPTG, and imaged in both cyan and NIR channels simultaneously (Figure 7 A). The mean NIR/cyan ratios of bacteria treated with 20, 50, 100, and 1000 μM of IPTG were determined to be, respectively, 1.7, 2.4, 3.0 and 3.7 fold higher than that of bacteria treated with 10 μM of IPTG (Figure 7 B). These numbers correlate well with in vitro quantification of **BeCA** in total RNAs isolated from the bacteria treated with different concentrations of IPTG (Figure 7 C and Figure S13). This result clearly shows that the ratiometric images obtained by our color‐shifting FLAP system can be utilized to evaluate the expression levels of RNA transcripts.

Besides the determination of the intracellular concentration of a particular ROI at the population (bulk) level, the ratiometric images obtained from the **BeCA**‐**BC6** system could provide more precise information at the single‐cell level. As shown in Figure 7 D, the amounts of RNA transcripts in single bacteria (cells **a** and **b**) do not always correlate well with the NIR fluorescence intensities. This might be due to variations in intracellular probe concentration, cell thickness and focal plane. Owing to the dual‐fluorescence nature of **BeCA**‐**BC6**, we can use the cyan channel to correct for these discrepancies. Indeed, the ratiometric signals suggest that cells **a** and **b** in Figure 7 D had similar levels of intracellular **BeCA** transcripts. Extensive heterogeneity of RNA transcription at the single‐cell level in *E. coli* due to stochasticity in transcriptional regulation could also be revealed in the ratiometric images especially at high IPTG concentration (Figure 7 D, cells **c**–**e**).^[27]^

## Conclusion

In summary, we have presented the development of a color‐shifting NIR fluorescent aptamer‐fluorophore module **BeCA**‐**BC6** for live‐cell RNA imaging. To achieve that, we exploited the intramolecular spirocyclization of an environmentally sensitive hybrid fluorophore, **BC**. It exists in a dynamic equilibrium between a cyan‐fluorescent, spirocyclic, closed form and a NIR‐fluorescent, zwitterionic, open form. In vitro selection, truncation, and mutation studies rendered a 38‐nt minimal aptamer **BeCA**, which binds selectively to the zwitterionic form of **BC**. By introducing electron‐withdrawing fluorine atoms and electron‐deficient amine groups to **BC**, we obtained a series of **BC** analogs with various open‐closed ratios. The best probe **BC6** exists primarily in the closed state with an emission maximum of 478 nm and emits at 684 nm when bound to **BeCA**, representing the most NIR‐shifted FLAP in the literature. Thus, **BeCA‐BC6** is a valuable addition to the RNA imaging toolbox due to the lack of FLAPs functioning in the NIR window where cells have much lower absorption (less phototoxicity), lower auto‐fluorescence and deeper penetration.

Moreover, we demonstrated that **BC6** showed an emission ratio change (cyan/NIR) as high as 15‐fold upon binding to **BeCA**. In live‐cell RNA imaging experiments, the cyan fluorescence from unbound probes reveals the intracellular probe delivery and its distribution while the NIR fluorescence indicates the RNA location. **BeCA‐BC6** is the only aptamer‐fluorophore pair which allows simultaneous imaging of both free fluorophore and the complex. We used this feature to obtain ratiometric images of bacteria with different expression levels of **BeCA**. Ratiometric images, in contrast to single‐color fluorescence images, do not suffer from problems associated with varying dye uptake, heterogeneous probe distribution, probe instability, cell morphology and fluctuations in focal plane. Thus, the dual‐color feature of **BeCA‐BC6** allowed us to more accurately analyze the expression levels of RNA transcripts and revealed the transcriptional heterogeneity at the single‐cell level.

We also showed that multiple repeats of **BeCA** can be genetically fused to more complex and short‐lived mRNAs to increase the signal‐to‐background ratio. Combined with the ratiometric advantages, **BeCA‐BC6** can be used to precisely analyze the abundance of target RNAs, which could provide new insights in gene expression, regulation, developmental plasticity and disease diagnostics.^[28]^

The spirocyclic probes including **BC6** have excellent membrane permeability compared to their zwitterionic counterparts.^[20d]^ Further, spirocyclic probes are less likely to be switched to their zwitterionic forms by other cellular components, thus resulting in less unspecific NIR staining inside the cells. As demonstrated in this study, aptamer binding can significantly change their biophysical properties and modulate equilibrium dynamics. In the future, by employing library mutagenesis and fluorescence activated cell sorting (FACS), it would be possible to evolve tailored color‐shifting fluorophore‐aptamer pairs with various colors, higher brightness, better affinity and improved thermal stability.

## Conflict of interest

The authors declare no conflict of interest.

## Supporting information

As a service to our authors and readers, this journal provides supporting information supplied by the authors. Such materials are peer reviewed and may be re‐organized for online delivery, but are not copy‐edited or typeset. Technical support issues arising from supporting information (other than missing files) should be addressed to the authors.

Supporting Information
