# Supplementary material for: A Color‐Shifting Near‐Infrared Fluorescent Aptamer–Fluorophore Module for Live‐Cell RNA Imaging
Source: Angew Chem Int Ed Engl. 2021 Aug 20;60(39):21441–8. doi: 10.1002/anie.202107250 (PMC8518806; doi:10.1002/anie.202107250)
Supplement: Supplementary file 1 — Supporting Information [file ANIE-60-21441-s001.pdf]

Angewandte Chemie
Eine Zeitschrift der Gesellschaft Deutscher Chemiker

Supporting Information

A Color-Shifting Near-Infrared Fluorescent Aptamer–Fluorophore Module for Live-Cell RNA Imaging

Jingye Zhang, Lu Wang, Andres Jäschke, and Murat Sunbul**

anie_202107250_sm_miscellaneous_information.pdf

Table of Contents

1. Supplemental Figures and Tables 2
2. General Materials and Instruments 15
3. Synthesis and Characterization 15
4. Selection, Truncation and Mutation Study 26
   - 4.1 DNA library preparation 26
   - 4.2 Resin preparation 27
   - 4.3 Selection protocol 28
   - 4.4 Sanger sequencing 28
   - 4.5 Activity screening of sequenced RNA pool 29
   - 4.6 Sequence alignment 29
   - 4.7 Dissociation constant measurement of active sequences 29
   - 4.8 Truncation analysis of RNA8 29
   - 4.9 Mutant activity screening 30
5. Substrate Screening for BeCA 30
   - 5.1 Absorbance and emssion spectra of BC 1–7 30
   - 5.2 Absorbance spectra of BC 1–7 at various dielectric constants 30
   - 5.3 Fluorescence light-up studies of BC 1–7 upon binding BeCA 31
6. Characterization of BeCA-BC6 31
   - 6.1 Absorption, excitation and emission spectra of BeCA-BC6 31
   - 6.2 Dissociation constant measurement of BeCA-BC6 31
   - 6.3 Temperature dependence of BeCA-BC6 31
   - 6.4 Magnesium and potassium concentration dependence of BeCA-BC6 31
   - 6.5 Photophysical properties of BC6 and BeCA-BC6 31
7. Live-cell RNA imaging using color-shifting BeCA-BC6 32
   - 7.1 Cloning of *pET28-BeCA* 32
   - 7.2 Cloning of *pET28-gfp-BeCA*$_8$ 32
   - 7.3 Cloning of *pAV-Tornado-BeCA*$_4$ 33
   - 7.4 General method for live-cell imaging in bacteria 33
   - 7.5 Total RNA isolation and gel analysis of BeCA 34
   - 7.6 General method for circular RNA imaging in mammalian cells 34
8. Supplemental Spectra 35
9. References 57

1. Supplemental Figures and Tables

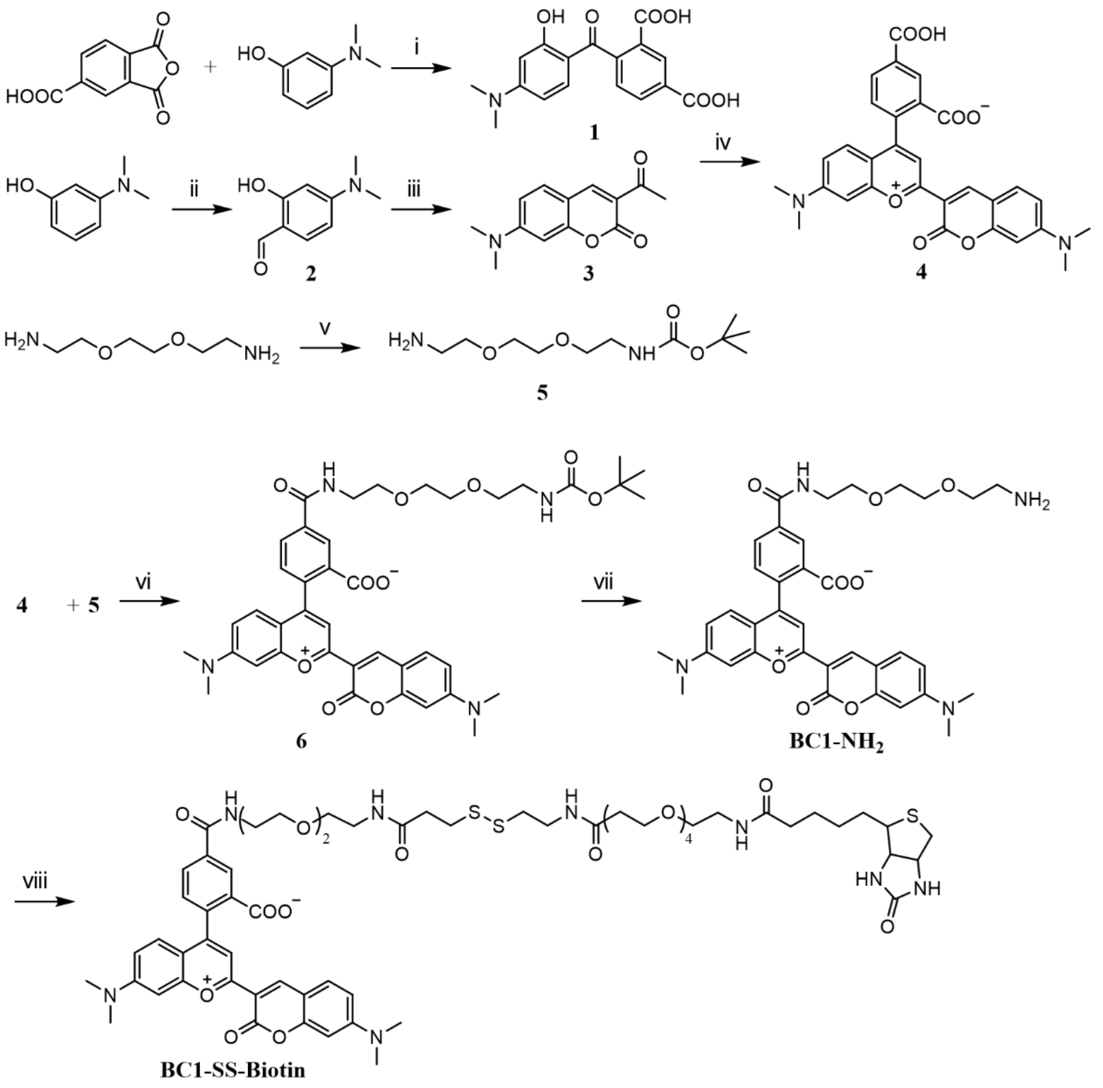

Scheme S1. Synthetic routes of SELEX ligands, **BC1-NH$_2$** and **BC1-SS-Biotin**: (i) toluene, 110 ºC. 24 h; (ii) $POCl_3$, DMF, 70 ºC, 1 h; (iii) EAA, EtOH, 85 ºC, 5 h; (iv) H_2SO_4, 90 ºC, 6 h; (v) Boc_2O, DCM, r.t., 2 h; (vi) TBTU, DIPEA, DMF, r.t., 12 h; (vii) TFA, DCM, r.t., 2 h; (viii) NHS-SS-PEG$_4$-Biotin, DMF, r.t., 40 min.

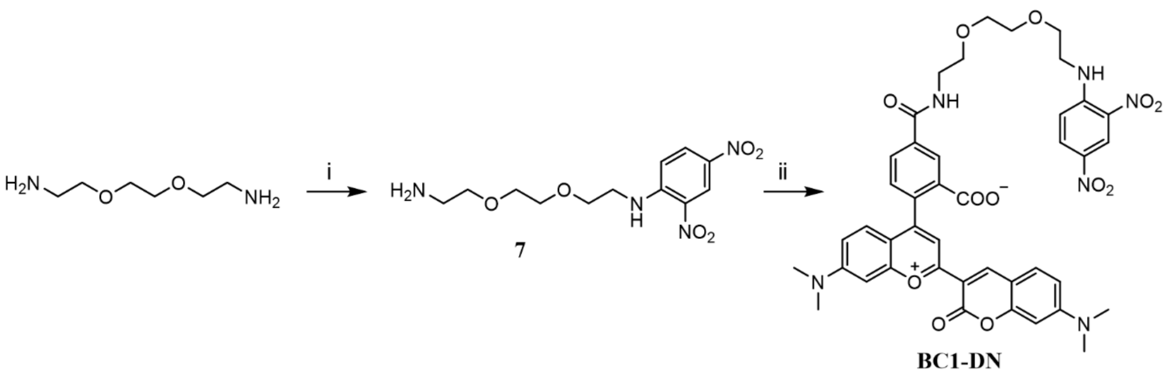

Scheme S2. Synthetic routes of **BC1-DN**: (i) DNFB, DCM, r.t., 1 h; (ii) **4**, BOP, DIPEA, DMF, r.t. 1 h.

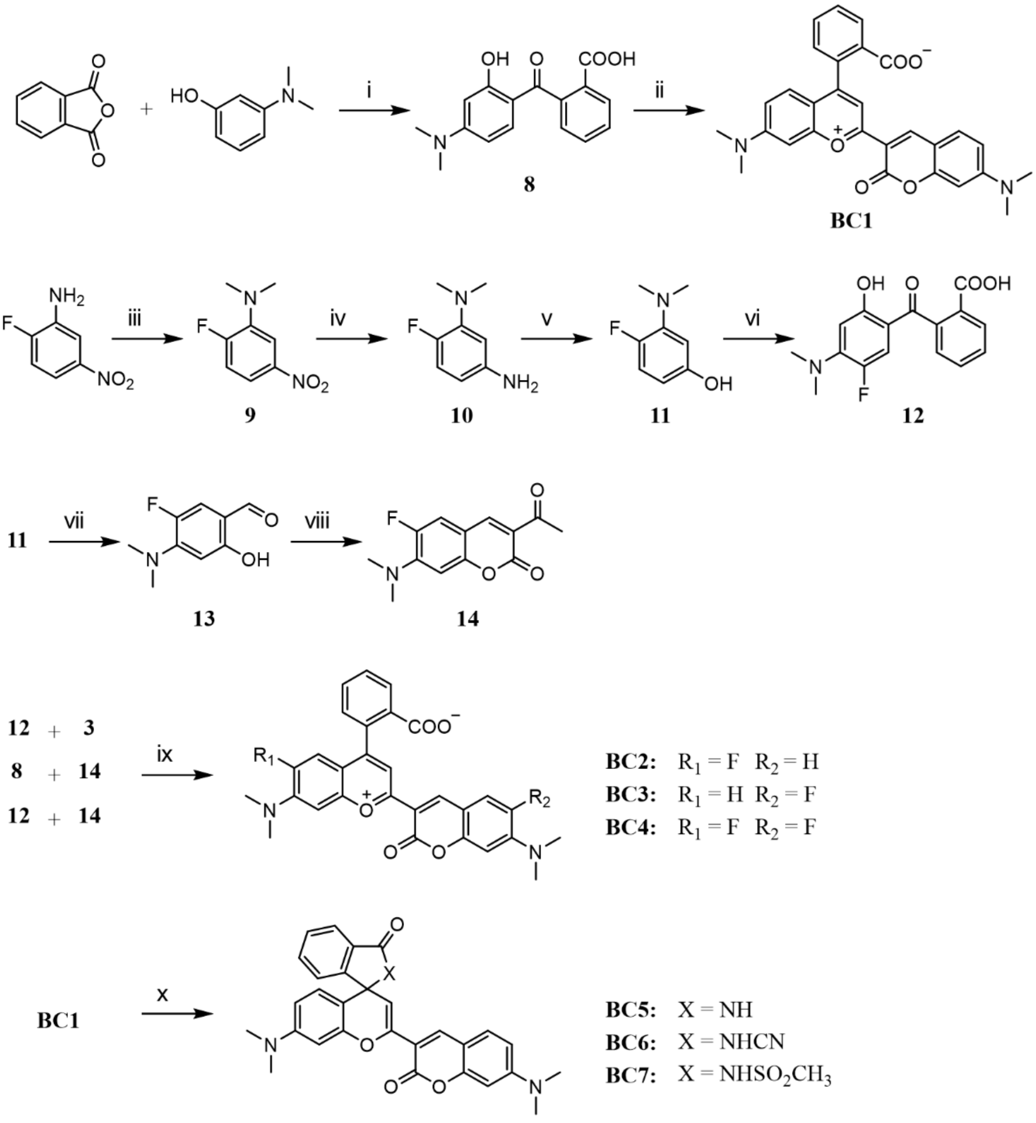

Scheme S3. Synthetic routes of **BC 1–7**: (i) toluene, 110 ºC. 24 h; (ii) **3**, H_2SO_4, 90 ºC, 6 h; (iii) CH_2O, $NaBH_4$, H_2SO_4, THF, r.t.; (iv) Pd/C, H_2, EtOAc, r.t., 4 h; (v) $NaNO_2$, H_2SO_4, $Cu(NO_3)_2·3H_2O$, Cu_2O, r.t.; (vi) phthalic anhydride, $ZnCl_2$, 180 ºC, 6 h, then NaOH, 90 ºC, 8 h; (vii) $POCl_3$, DMF, 75 ºC, 2 h; (viii) EAA, EtOH, 85 ºC, 4 h; (ix) H_2SO_4, 90 ºC, 24 h; (x) $POCl_3$, DCM, 60 ºC, 4 h, then NH3(aq)/cyanamide/methanesulfonamide ACN, Et_3N, r.t., 2 h.

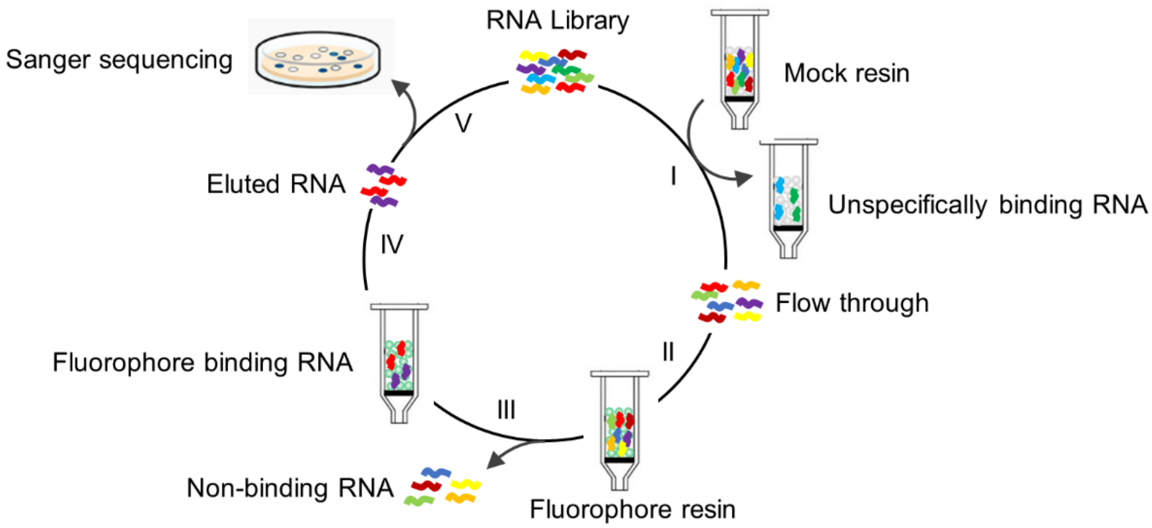

Figure S1. Scheme showing a typical SELEX cycle for the selection of benzopyrylium-coumarin (**BC**) fluorophore binding aptamers. **I**-Negative selection, **II**-Binding, **III**-Washing, **IV**-Elution, **V**-Reverse transcription, PCR, and *in vitro* transcription.

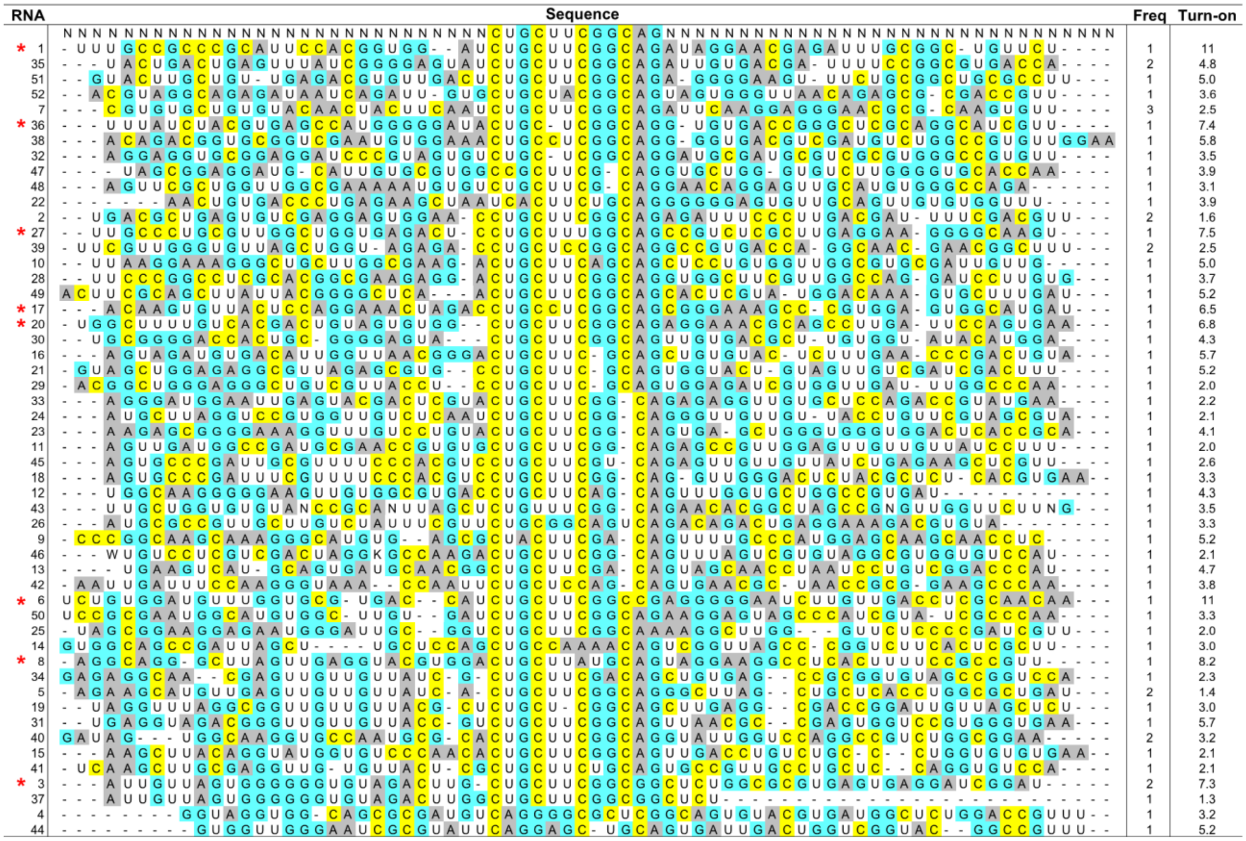

Figure S2. Screening of the selected RNAs for binding to **BC1**. Sanger sequencing results of the selected RNA sequences, their frequencies and fluorescence turn-on factors are shown. The fluorescence turn-on factors were determined by dividing the maximal fluorescence intensities of **BC1-DN** (100 nM) in the presence of RNA aptamers (10 μM) to that of **BC1-DN** (100 nM). RNA transcripts contain the primer binding sequences at the 5' and 3' ends. The highly active RNAs with a turn-on factor > 6 were indicated with a red asterisk. The fluorescence assay was performed with an excitation wavelength of 652 nm ± 5 nm in a buffer containing 20 mM Hepes (pH 7.4), 5 mM $MgCl_2$, and 125 mM KCl.

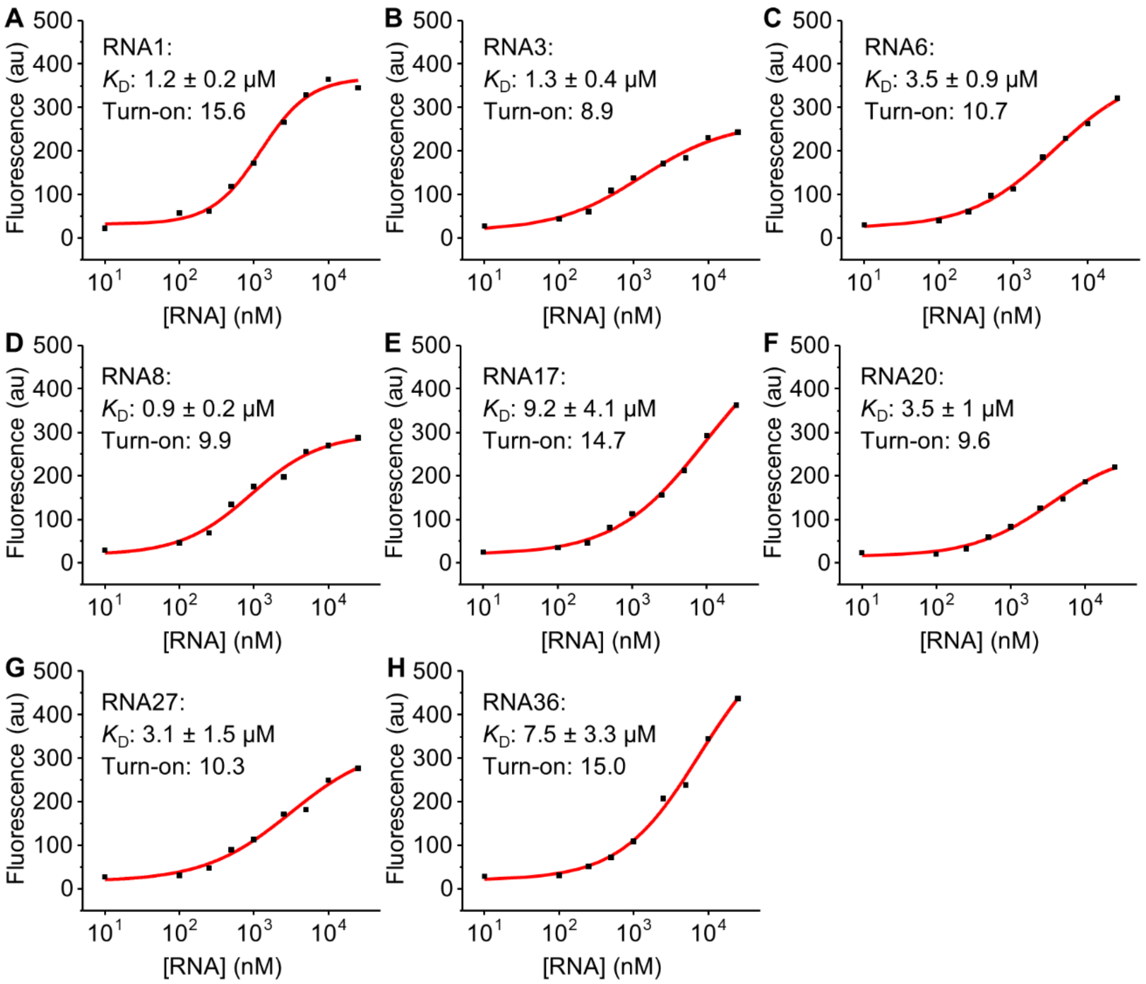

Figure S3. Characterization of the eight highly active RNA sequences. The K_D values were determined by titrating **BC1-DN** (100 nM) with different amounts of RNA (0 – 25 μM) individually. The fluorescence turn-on factors were determined by dividing the maximal fluorescence intensities of **BC1-DN** (100 nM) in the presence of RNA mutants (25 μM) by that of **BC1-DN** (100 nM). For the fluorescence measurements, an excitation wavelength of 652 ± 5 nm and a buffer containing 20 mM Hepes (pH 7.4), 5 mM $MgCl_2$, and 125 mM KCl were used.

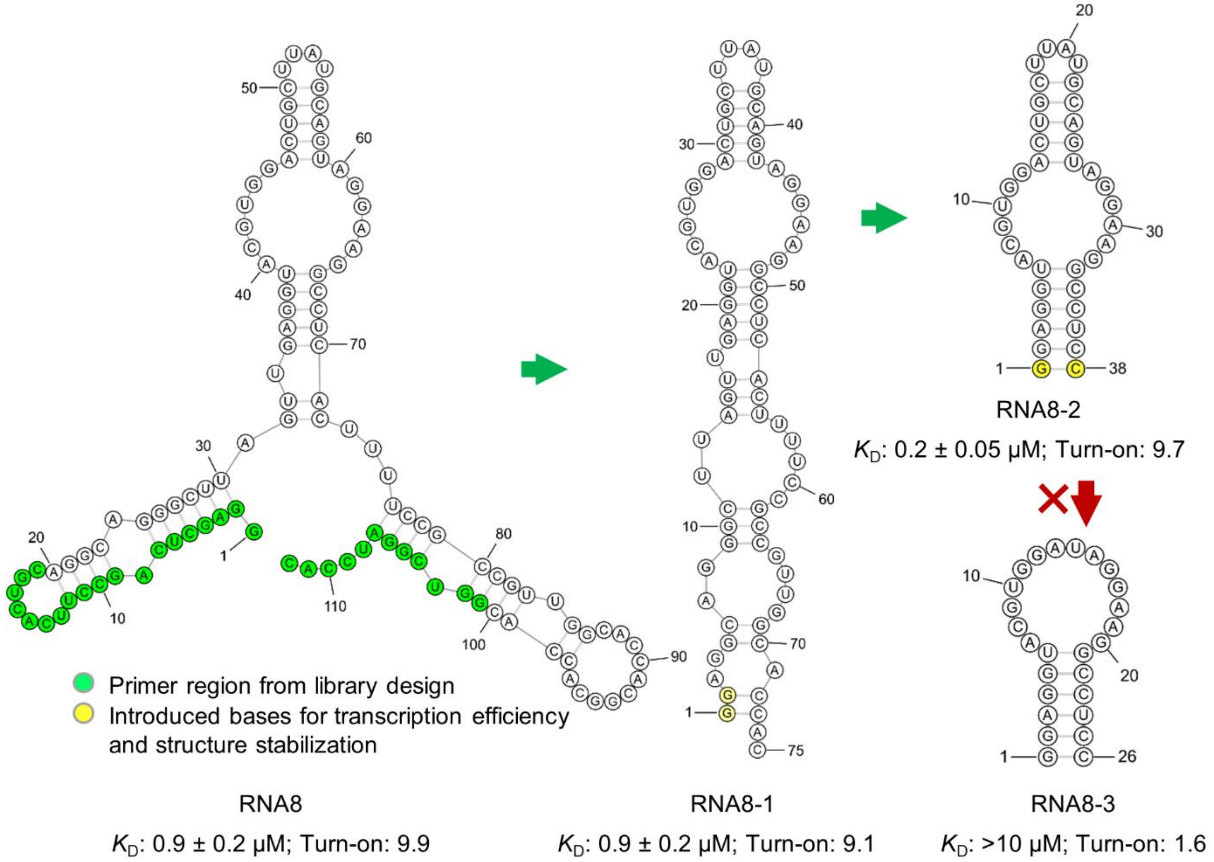

Figure S4. Truncation analysis of RNA8. The predicted secondary structures of RNA8 and its truncated versions, RNA8-1, RNA8-2, RNA8-3 are shown. The K_D values were determined by titrating **BC1-DN** (100 nM) with different amounts of RNA (0 – 10 μM) individually. The fluorescence turn-on factors were determined by dividing the maximal fluorescence intensities of **BC1-DN** (100 nM) in the presence of RNA mutants (10 μM) by that of **BC1-DN** (100 nM). For the fluorescence measurements, an excitation wavelength of 652 ± 5 nm and a buffer containing 20 mM Hepes (pH 7.4), 5 mM $MgCl_2$, and 125 mM KCl were used.

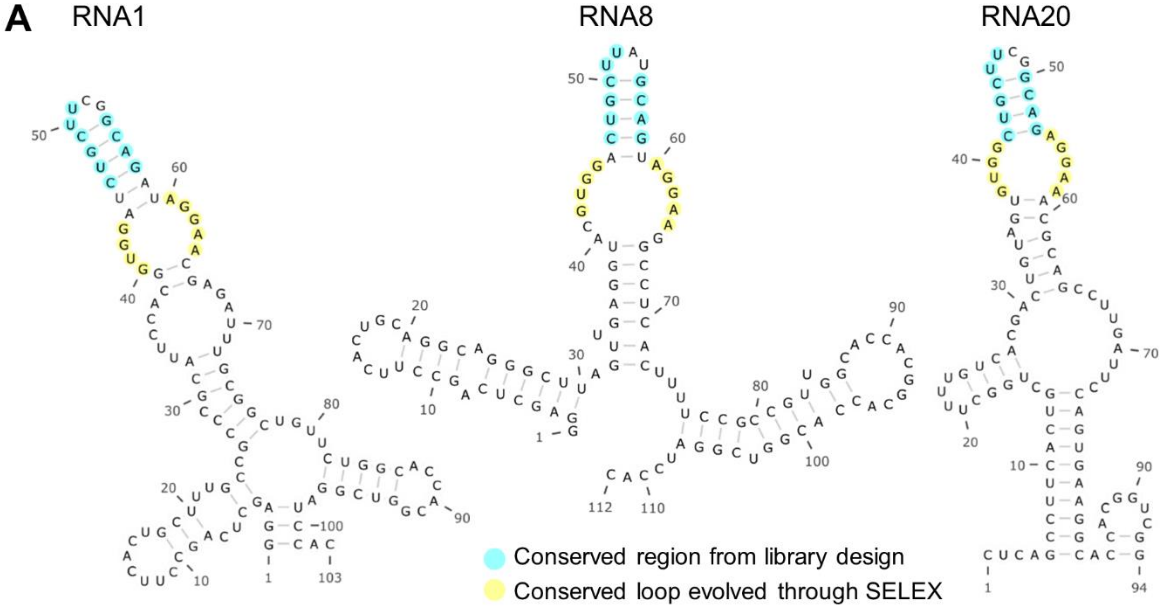

B RNA8-2 mutation chart

Mutational site

RNA	1	2	3	4	5	6	7	8	9	10	11	12	13	14	15	16	17	18	19	20	21	22	23	24	25	26	27	28	29	30	31	32	33	34	35	36	37	38	K_D	Turn-on
8-2	G	G	A	G	G	U	A	C	G	U	G	G	A	C	U	G	C	U	U	A	U	G	C	A	G	U	A	G	G	A	A	G	G	C	C	U	C	C	0.23 ± 0.05	9.7
M1	G	G	A	G	G	C	A	C	G	U	G	G	A	C	U	G	C	U	U	A	U	G	C	A	G	U	A	G	G	A	A	G	G	C	C	U	C	C	1.1 ± 0.47	9.4
M2	G	G	A	G	G	G	A	C	G	U	G	G	A	C	U	G	C	U	U	A	U	G	C	A	G	U	A	G	G	A	A	G	C	C	C	U	C	C	1.7 ± 0.48	5.0
M3	G	G	A	G	G	U	A	C	G	U	G	G	A	C	U	G	C	U	U	A	U	G	C	A	G	U	A	G	G	A	A	G	A	C	C	U	C	C	0.62 ± 0.19	6.1
M4	G	G	A	G	G	U	A	C	G	U	G	G	A	C	U	G	C	U	U	A	U	G	C	A	G	U	A	G	G	A	A	G	C	C	C	U	C	C	4.6 ± 1.4	10.2
M5	G	G	A	G	G	U	A	C	G	U	G	G	A	C	U	G	C	U	U	A	U	G	C	A	G	U	A	G	G	A	A	G	U	C	C	U	C	C	1.3 ± 0.27	6.8
M6	G	G	A	A	A	U	A	C	G	U	G	G	A	C	U	G	C	U	U	A	U	G	C	A	G	U	A	G	G	A	A	G	G	U	U	U	C	C	0.35 ± 0.05	10.2
M7	G	G	A	G	-	U	A	C	G	U	G	G	A	C	U	G	C	U	U	A	U	G	C	A	G	U	A	G	G	A	A	G	G	-	C	U	C	C	0.20 ± 0.03	10.5
M8	G	G	A	G	G	U	-	C	G	U	G	G	A	C	U	G	C	U	U	A	U	G	C	A	G	U	A	G	G	A	A	G	G	C	C	U	C	C	1.1 ± 0.44	9.9
M9	G	G	A	G	G	U	A	-	G	U	G	G	A	C	U	G	C	U	U	A	U	G	C	A	G	U	A	G	G	A	A	G	G	C	C	U	C	C	0.25 ± 0.06	6.7
M10	G	G	A	G	G	U	A	C	G	U	G	G	A	C	U	G	C	U	U	A	U	G	C	A	G	U	A	G	G	A	A	G	-	C	C	U	C	C	0.77 ± 0.18	7.1
M11	G	G	A	G	G	U	A	C	G	U	G	G	G	C	U	G	C	U	U	A	U	G	C	A	G	C	A	G	G	A	A	G	G	C	C	U	C	C	0.27 ± 0.08	8.5
M12	G	G	A	G	G	U	A	C	G	U	G	G	A	C	U	G	C	U	U	C	G	G	C	A	G	U	A	G	G	A	A	G	G	C	C	U	C	C	0.33 ± 0.12	9.3

Figure S5. Identification of common motif and mutation study of RNA8-2 (**BeCA)**. (A) Predicted secondary structures of RNA1, RNA8, and RNA20 are shown. The conserved nucleotides evolved through SELEX and the conserved nucleotides from the library design are highlighted in yellow and blue, respectively. (B) Activity screening of **BeCA** mutants. RNA sequences of all 12 mutants are shown with mutation sites highlighted in green. The K_D values were determined by titrating **BC1-DN** (100 nM) with different amounts of RNA (0 – 10 μM) individually. The fluorescence turn-on factors were determined by dividing the maximal fluorescence intensities of **BC1-DN** (100 nM) in the presence of RNA mutants (10 μM) by that of **BC1-DN** (100 nM). For the fluorescence measurements, an excitation of 652 ± 5 nm was used in a buffer containing 20 mM Hepes (pH 7.4), 5 mM $MgCl_2$, and 125 mM KCl.

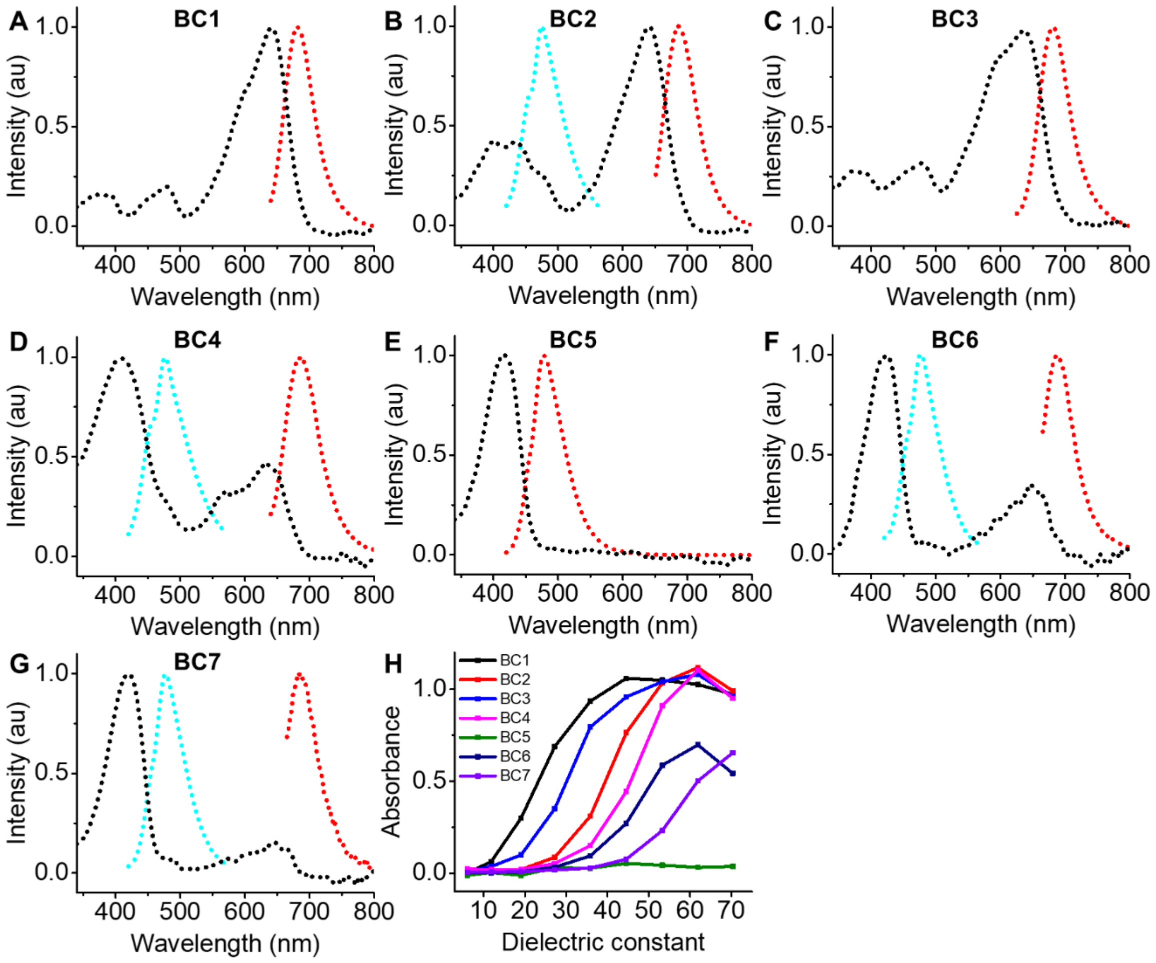

Figure S6. Spectroscopic characterization of **BC 1–7**. (A–G) Normalized absorbance and emission spectra of **BC 1–7** (1 μM) in 20 mM Hepes containing 0.1% Triton X-100. Black, cyan and red dotted lines represent the absorbance, emission spectra of the spirocyclic form, and the emission spectra of the zwitterionic form, respectively. (H) Normalized absorbance of **BC 1–7** (5 μM) in zwitterionic form in water/dioxane mixtures (v/v: 10/90 – 90/10) as a function of dielectric constant.

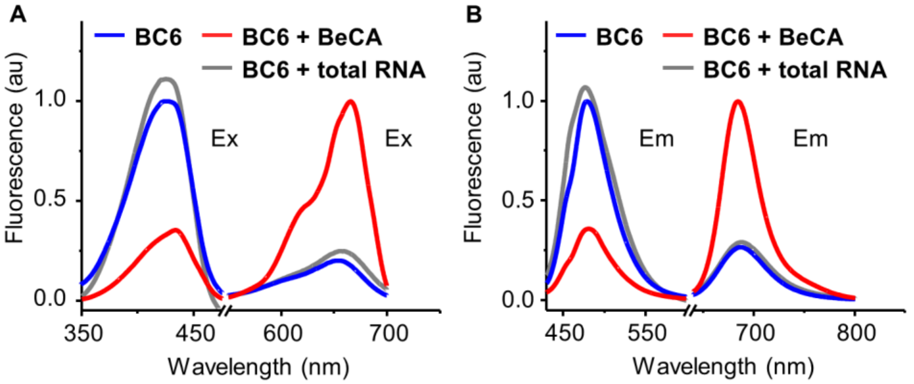

Figure S7. **BC6** specifically binds **BeCA**. Excitation (A) and emission (B) spectra of **BC6** (1 μM) in the presence of **BeCA** (25 μM, 0.3 mg/mL) or total RNA (0.3 mg/mL). The spectra were measured in a buffer containing 20 mM Hepes (pH 7.4), 5 mM $MgCl_2$, 125 mM KCl, and 0.05% Triton X-100.

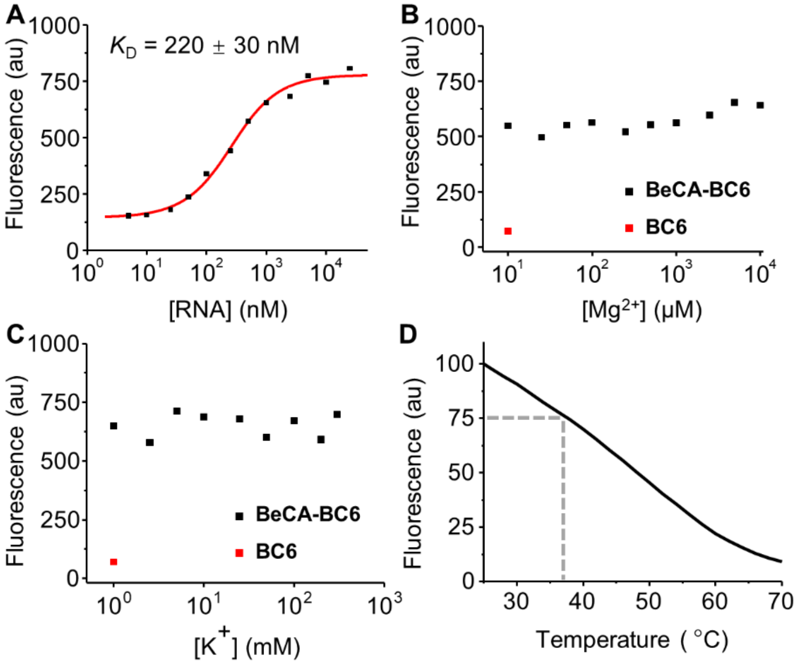

Figure S8. Characterization of **BeCA-BC6** complex. (A) K_D curve of **BeCA-BC6** complex. The K_D value was determined by titrating **BC6** (100 nM) with different amounts of **BeCA** (0 – 25 μM) individually in a buffer solution containing 20 mM Hepes (pH 7.4), 5 mM $MgCl_2$, and 125 mM KCl. (B) Magnesium dependence of **BeCA-BC6** complex (**BeCA**: 5 μM, **BC6**: 5 μM) measured in 20

mM Hepes (pH 7.4) and 125 mM KCl. (C) Potassium dependence of **BeCA-BC6** complex (**BeCA**: 5 μM, **BC6**: 5 μM) measured in 20 mM Hepes (pH 7.4) and 5 mM $MgCl_2$. (D) Temperature dependent fluorescence of **BeCA-BC6** complex (**BeCA**: 5 μM, **BC6**: 5 μM) measured in a buffer solution containing 20 mM Hepes (pH 7.4), 5 mM $MgCl_2$, and 125 mM KCl. For the fluorescence measurements in all panels, an excitation wavelength of 665 ± 5 nm was used, and the fluorescent intensities at the emission maximum of 684 nm were plotted.

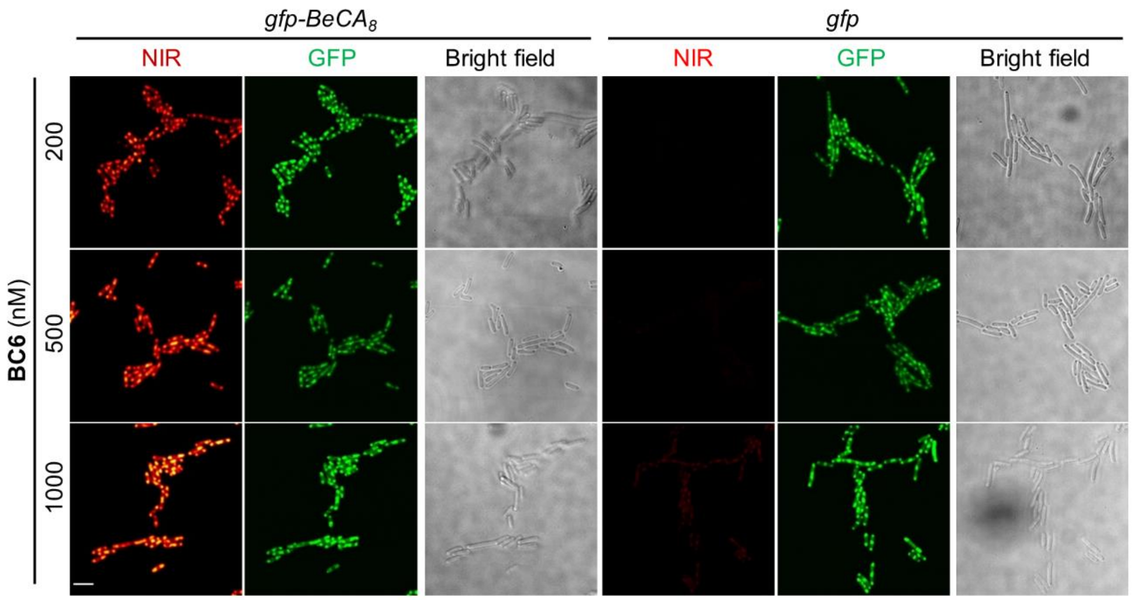

Figure S9. Live-cell confocal images of *gfp* mRNA tagged with **BeCA** in *E. coli*. Bacteria were transformed with either *gfp-BeCA*$_8$ or *gfp* plasmid and the mRNA expression was induced by the addition of IPTG. Cells were incubated with **BC6** (200, 500, 1000 nM) for 15 min and imaged. Images in green and NIR channels were acquired using 488 nm and 640 nm lasers, respectively. Scale bar, 5 μm.

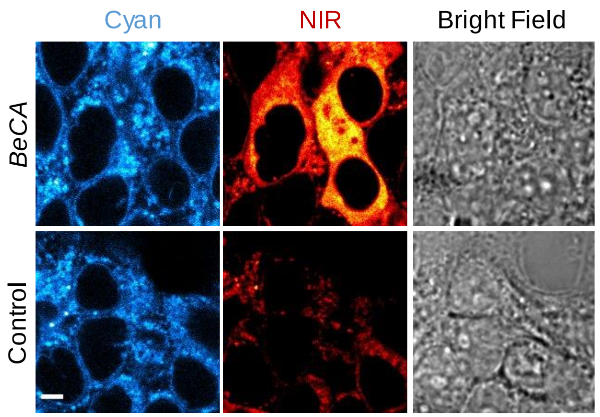

Figure S10. Confocal images of live HEK293T cells expressing circular **BeCA** aptamers. Cells were transfected with *P$_{U6}$-Tornado-BeCA$_4$* plasmid and incubated with **BC6** (500 nM). Untransfectected cells were used as a negative control. Images in cyan and NIR channels were acquired using 405 nm and 640 nm lasers, respectively. Scale bar, 5 µm.

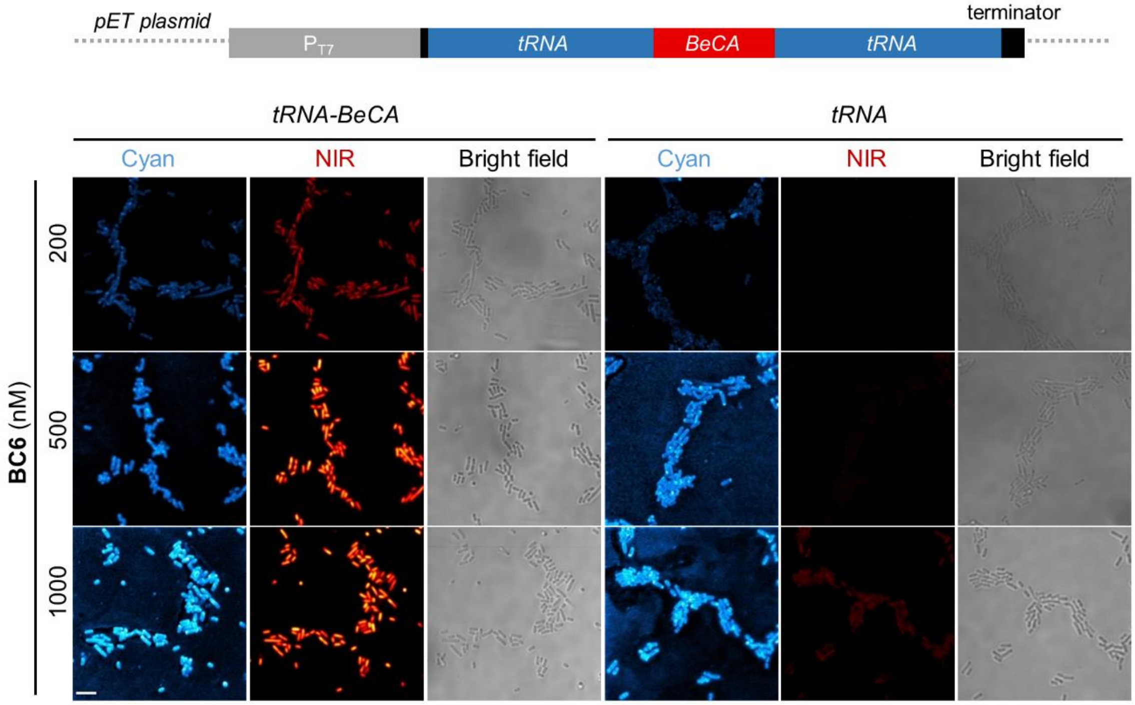

Figure S11. Live-cell confocal images of **BeCA** in *E. coli*. Bacteria were transformed with either a *tRNA-BeCA* or *tRNA* plasmid, and the RNA expression was induced by the addition of IPTG. Cells were incubated with **BC6** (200, 500, 1000 nM) and imaged. Images in cyan and NIR channels were acquired using 405 nm and 640 nm lasers, respectively. Scale bar, 5 µm.

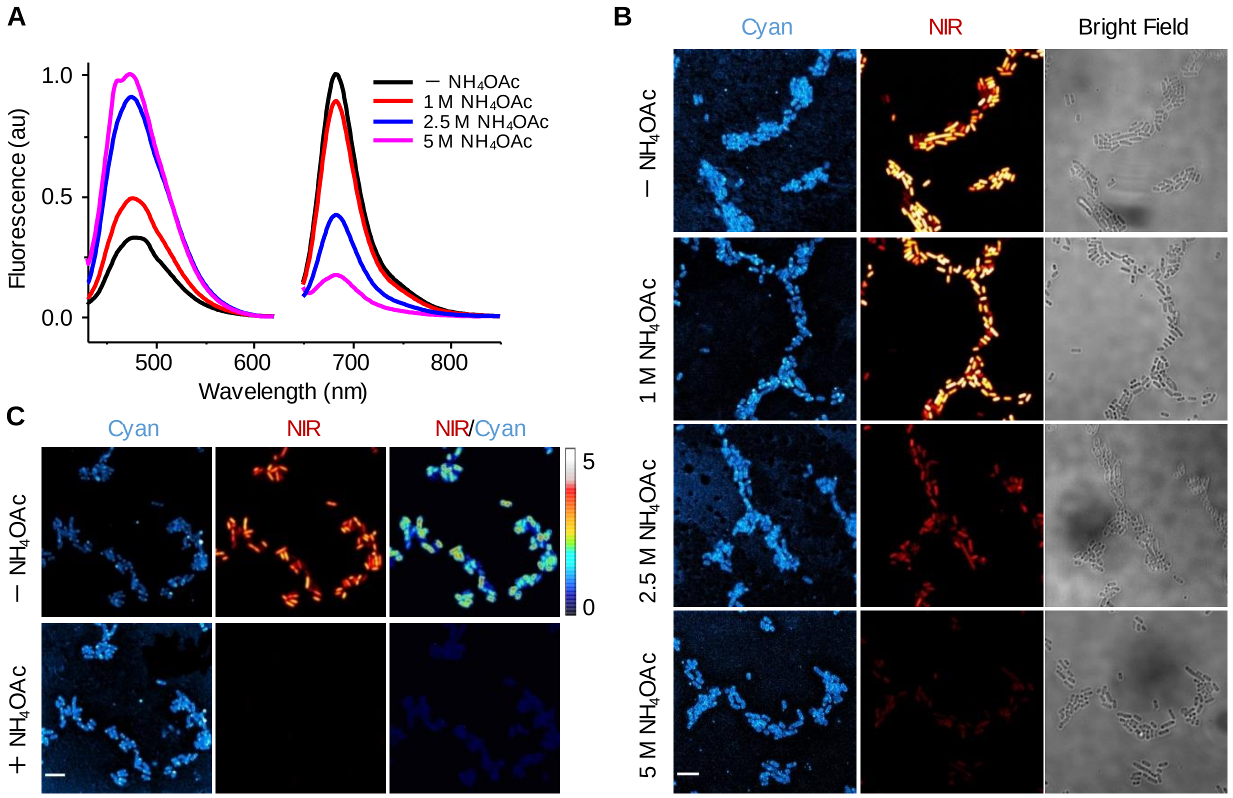

Figure S12. High concentration of ammonium acetate (NH_4OAc) disrupts the interaction between **BeCA** and **BC6**. (A) NH_4OAc dependent fluorescence decrease of **BeCA-BC6** complex. **BeCA-BC6** (**BeCA**: 10 μM, **BC6**: 500 nM) was incubated with varying concentrations of NH_4OAc (0, 1, 2.5, and 5 M, pH = 7) in a buffer containing 20 mM Hepes (pH 7.4), 5 mM $MgCl_2$, 125 mM KCl and 0.1% Trition X-100. The cyan and NIR fluorescence intensites were measured using excitation wavelengths of 420 ± 5 nm and 630 ± 5 nm, respectively. (B) Imaging of **BeCA**-expressing bacteria treated with NH_4OAc. The images were taken 15 min after adding varying concentrations of NH_4OAc (0 – 5 M). (C) Ratiometric images of **BeCA**-expressing bacteria before and after addition of NH_4OAc (5 M). For (B) and (C), bacteria were transformed with *tRNA-BeCA* plasmid and the RNA expression was induced by addition of IPTG (1 mM). Cells were incubated with **BC6** (500 nM) for 1h. Images in cyan and NIR channels were acquired using 405 nm and 640 nm lasers, respectively. Scale bar, 5 μm.

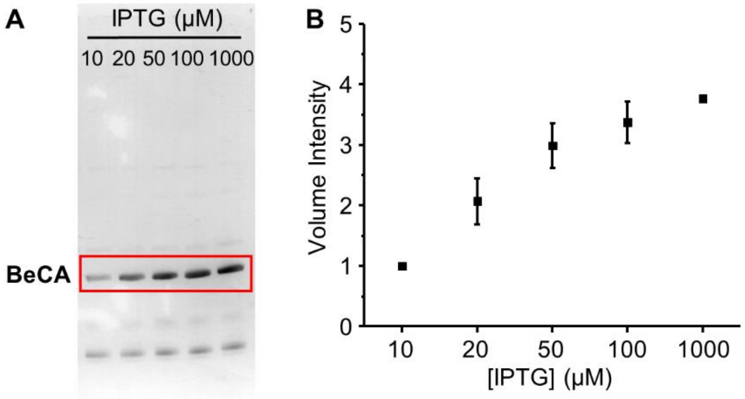

Figure S13. *In vitro* quantification of **BeCA** in total RNAs isolated from bacteria treated with different amounts of IPTG (10 – 1000 μM). (A) Denaturing polyacrylamide gel analysis of **BeCA**. Under each condition, the same number of bacteria was used to isolate total RNAs. OD_{600} values were used to quantify the number of bacteria. (B) Normalized band volume intensity of **BeCA**. The band intensities of different samples were normalized to that of the sample treated with 10 μM IPTG. Mean ± standard deviation (s.d.) are shown (*n=2* gels from two independent experiments).

Table S1. Photophysical properties of **BC6** and **BeCA-BC6**.

	λ_{abs} (nm)	λ_{em} (nm)	ε ($mol^{-1}cm^{-1}$)	Φ
**BC6**	420[a] / 652[b]	478[a] / 686[b]	27200[a] / 11500[b]	0.04[a] / 0.08[b]
**BeCA-BC6**	n.d.[a] / 665[b]	n.d.[a] / 684[b]	n.d.[a] / 52700[b]	n.d.[a] / 0.07[b]

λ_{abs}: maximum absorption wavelength; λ_{em}: maximum emission wavelength; ε: molar extinction coefficient; Φ: quantum yield; n.d.: not detected; [a] spirocyclic form; [b] zwitterionic form. The measurements were performed in a buffer solution containing 20 mM Hepes (pH 7.4), 5 mM $MgCl_2$, 125 mM KCl, and 0.05% Triton X-100.

2. General Materials and Instruments

All chemical reagents and solvents for synthesis were purchased from Sigma-Aldrich GmbH, Alfa Aesar GmbH & Co. KG and Tokyo Chemical Industry Co. and used without further purification unless noted otherwise. Deuterated solvents for NMR spectroscopy were purchased from Euriso-Top GmbH. Reactions were monitored by thin layer chromatography (TLC) performed on Polygram Sil G/UV254 TLC plates (Macherey-Nagel GmbH & Co KG). Flash column chromatography was conducted using silica gel from Aldrich with a pore size of 60 Å and a particle size range of 40 – 63 µm. Preparative RP-HPLC was performed on a 1100 HPLC (Agilent Technologies, Inc.) equipped with a 5 µm C18 Phenomenex Luna reversed-phase column (Phenomenex, Inc.) using gradients of solvent A consisting of 0.1% (v/v) trifluoroacetic acid in water and solvent B consisting of 0.1% (v/v) trifluoroacetic acid in acetonitrile. A typical gradient was from 10% to 95% B within 60 min. The HPLC-purified fractions were dried on an Alpha 1-4 LD lyophilizer (Martin Christ Gefriertrocknungsanlagen GmbH) equipped with a vacuum pump (Vacuubrand GmbH & Co KG). All NMR spectra were recorded on a Varian Mercury Plus 300 or 500 MHz. The chemical shifts are reported in parts per million (ppm) and the coupling constants are given in hertz (Hz). High-resolution mass spectrometry (HRMS) was performed on a Bruker microTOF-QII mass spectrometer. Absorption spectra were recorded on a Cary 50 UV-Visible spectrophotometer (Agilent Technologies, Inc.) at 25 °C unless noted. Fluorescence measurements were performed on a FP-6500 fluorescence spectrometer (JASCO) at 25 °C unless noted. RNA concentrations were determined using a NanoDrop ND-1000 spectrophotometer. All oligonucleotides were purchased from Integrated DNA Technologies, Inc. All sequencing experiments were carried out at Microsynth Seqlab. All enzymes used for cloning were purchased from Thermo Fisher Scientific Inc. unless noted otherwise. The plasmids were purchased from Addgene (1GFP, #29663; pAV-U6+27-Tornado-Broccoli, #124360) and Merck KGaA (pET-28a(+)). Bacterial strains were purchased from Thermo Fisher Scientific Inc.

3. Synthesis and Characterization

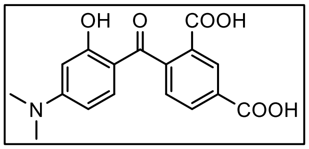

4-(4-(dimethylamino)-2-hydroxybenzoyl)isophthalic acid (1): 3-Dimethylaminophenol (2.20 g, 16.0 mmol) was dissolved in toluene (50 mL) and heated to 60 °C. Powdered trimellitic anhydride (3.84 g, 20.0 mmol) was added while stirring. The mixture was refluxed for 24 h and cooled down to room temperature. The residue was filtered off, washed with toluene (3 × 30 mL), dissolved in methanol (80 mL), and refluxed for 10 min. Then, acetic acid (20 mL) was added and the solvent was

removed under reduced pressure at 40 °C. The solid was refluxed in methanol (50 mL) for 2 h and kept at 4 °C overnight. The crystals formed were filtered off and washed with ice-cold methanol (50 mL) to give pure compound **1** as brown crystalline solid.

Yield: 724 mg, 2.24 mmol, 14%.

^{1}H NMR (300 MHz, C_2D_6OS) δ 8.45 (s, 1H), 8.16 (d, J = 7.8 Hz, 1H), 7.49 (d, J = 7.9 Hz, 1H), 6.77 (d, J = 9.1 Hz, 1H), 6.17 (d, *J* = 7.4 Hz, 1H), 6.08 (s, 1H), 2.97 (s, 6H).

^{13}C NMR (75 MHz, C_2D_6OS) δ 198.0, 166.5, 164.6, 156.2, 144.2, 134.2, 133.1, 132.1, 131.1, 130.4, 128.7, 109.9, 104.9, 97.4.

HRMS (ESI): m/z calc. for $C_{17}H_{15}NNaO_6$ 325.0792; found 325.0796, $[M+Na]^+$.

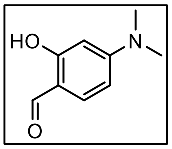

4-(dimethylamino)-2-hydroxybenzaldehyde (2): Phosphorus(V) oxychloride (4.67 mL, 50.0 mmol) was added dropwise into dimethylformamide (8 mL) at 0 ºC under argon. The mixture was stirred for 5 min at 0 ºC and then 40 minutes at room temperature. A solution of 3-(dimethylamino)phenol (3.43 g, 25.0 mmol) in dimethylformamide (2 mL) was added dropwise. The mixture was heated and stirred at 70 ºC for 1 h. The solution was poured onto crushed ice (approx. 50 g) and extracted with ethyl acetate. The combined organic phases were dried over anhydrous magnesium sulfate and evaporated. The crude product was purified by column chromatography using hexane:ethyl acetate mixtures (10:1 to 10:4).

Yield: 2.80 g, 17.0 mmol, 68%.

^{1}H NMR (300 MHz, $CDCl_3$) δ 9.52 (s, 1H), 7.26 (d, *J* = 5.7 Hz, 1H), 6.27 (dd, *J* = 8.9, 2.4 Hz, 1H), 6.07 (d, *J* = 2.4 Hz, 1H), 3.06 (s, 6H).

^{13}C NMR (75 MHz, $CDCl_3$) δ 192.4, 164.0, 156.1, 135.1, 111.7, 104.5, 97.2, 40.1.

HRMS (ESI): m/z calc. for $C_9H_{11}NNaO_2$ 188.0682; found 188.0684, $[M+Na]^+$.

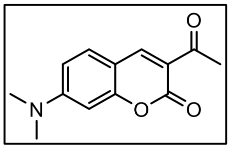

3-acetyl-7-(dimethylamino)chromen-2-one (3): Compound **2** (407 mg, 2.50 mmol) and ethyl acetoacetate (403 mg, 3.10 mmol) were dissolved in absolute ethanol (5 mL). Piperidine (9 drops) was slowly added and the solution was heated under reflux for 5 h. The mixture was cooled at 4 ºC overnight, leading to the formation of a yellow precipitate which was filtered off and washed with ice-cold ethanol to provide the pure product.

Yield: 450 mg, 1.95 mmol, 78%.

^{1}H NMR (300 MHz, $CDCl_3$) δ 8.44 (d, J = 0.7 Hz, 1H), 7.41 (d, J = 8.9 Hz, 1H), 6.63 (dd, J = 8.9, 2.5 Hz, 1H), 6.46 (d, J = 2.4 Hz, 1H), 3.12 (s, 7H), 2.67 (s, 3H).

^{13}C NMR (75 MHz, $CDCl_3$) δ 195.7, 160.7, 158.3, 154.9, 148.0, 131.6, 116.8, 110.0, 108.4, 97.0, 40.2, 30.6.

HRMS (ESI): m/z calc. for $C_3H_{13}NNaO_3$ 254.0788; found 254.0778, $[M+Na]^+$.

Compound 4: Compounds **1** (659 mg, 2.00 mmol) and **3** (462 mg, 2.00 mmol) were dissolved in concentrated sulfuric acid (5 mL) and stirred for 6 h at 90 °C. The mixture was cooled down to room temperature and ice (approx. 15 g) was added carefully. After adding 70% perchloric acid (1 mL), the mixture was filtered, and the precipitate was washed with water. The crude product was air-dried overnight and further purified by column chromatography using dichloromethane:methanol mixtures (10:1 to 10:3).

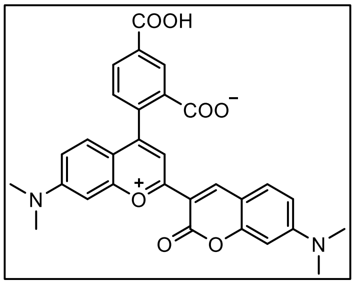

Yield: 356 mg, 680 µmol, 34%.

^{1}H NMR (300 MHz, C_2D_6OS) δ 9.11 (s, 1H), 8.64 (d, J = 1.7 Hz, 1H), 8.32 (dd, J = 8.0, 1.8 Hz, 1H), 8.11 (s, 1H), 7.66 (dd, J = 15.7, 8.5 Hz, 2H), 7.23 (s, 3H), 6.95 (d, *J* = 8.8 Hz, 1H), 6.68 (d, J = 2.3 Hz, 1H), 3.28 (s, 6H), 3.17 (s, 6H).

^{13}C NMR (126 MHz, C_2D_6OS) δ 166.4, 162.0, 158.4, 158.1, 158.1, 157.2, 156.9, 146.7, 140.1, 133.5, 133.1, 133.0, 131.9, 131.3, 130.7, 129.4, 118.0, 116.0, 112.5, 111.7, 110.2, 106.0, 97.3, 96.8, 40.7, 40.2.

HRMS (ESI): m/z calc. for $C_{30}H_{25}N_2O_7$ 525.1656; found 525.1639, $[M+H]^+$.

N-Boc-2,2'-(ethylenedioxy)diethylamine (5): To a solution of 2,2'-(Ethylenedioxy)bis(ethylamine) (4.45 g, 30.0 mmol) in anhydrous dichloromethane (200 mL) at 0 °C was added di-tert-butyl dicarbonate (1.20 g, 5.50 mmol) dissolved in anhydrous dichloromethane (100 mL) dropwise over a period of 1 h. The reaction mixture was allowed to warm up to room temperature and stirred for 2 h. The solution was washed with brine (2 × 100 mL), dried over anhydrous magnesium sulfate and the solvent was removed under reduced pressure. The transparent and highly viscous liquid was lyophilized and used without further purification.

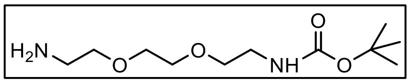

Yield: 967 mg, 3.90 mmol, 78%.

^{1}H NMR (300 MHz, $CDCl_3$) δ 3.61 (s, 4H), 3.52 (dt, *J* = 9.4, 5.2 Hz, 4H), 3.31 (q, *J* = 5.4 Hz, 2H), 2.87 (t, J = 5.2 Hz, 2H), 1.43 (s, 9H).

^{13}C NMR (75 MHz, $CDCl_3$) δ 156.0, 79.1, 73.5, 70.2, 41.8, 40.3, 28.4.

HRMS (ESI): m/z calc. for $C_{11}H_{25}N_2O_4$ 249.1809; found 249.1817, $[M+H]^+$.

BC1-NH$_2$: Compound **4** (105 mg, 200 µmol) and 2-(1H-benzotriazole-1-yl)-1,1,3,3-tetramethylaminium tetrafluoroborate (77.0 mg, 240 µmol) was dissolved in anhydrous dimethylformamide (1 mL). After 30 min stirring at room temperature, N,N-diisopropylethylamine (77.6 mg, 600 µmol) and **5** (99.2 mg, 400 µmol) was added. The solution was stirred overnight. The next day, the mixture was diluted with water (100 mL) and extracted with ethyl acetate (5 × 100 mL). The combined organic phases were dried over anhydrous magnesium sulfate and evaporated under reduced pressure. The obtained residue was purified by column chromatography using dichloromethane/methanol (10:0 to 10:2) to yield compound **6**. Compound **6** was dissolved in a trifluoroacetic acid:dichloromethane mixture (1:4) and stirred at room temperature for 2 h under argon. The solvent was removed under vacuum, and the blue residue was purified by RP-HPLC.

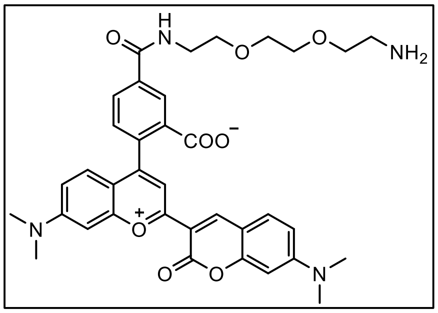

Yield: 16.0 mg, 24.5 µmol, 12%.

^{1}H NMR (500 MHz, C_2D_6OS) δ 8.68 (s, 1H), 8.39 (d, J = 1.5 Hz, 1H), 8.23 (dd, J = 8.0, 1.6 Hz, 1H), 7.69 (d, J = 8.9 Hz, 1H), 7.44 (d, J = 8.1 Hz, 1H), 6.85 (dd, J = 9.0, 2.5 Hz, 1H), 6.69 (d, J = 1.5 Hz, 1H), 6.61 (d, J = 2.4 Hz, 1H), 6.54 (d, J = 1.4 Hz, 2H), 6.38 (s, 1H), 3.61 – 3.54 (m, 8H), 3.47 (q, J = 5.9 Hz, 4H), 3.08 (s, 6H), 2.97 (s, 6H).

^{13}C NMR (126 MHz, C_2D_6OS) δ 168.7, 165.3, 158.9, 156.1, 155.9, 154.3, 152.3, 152.2, 147.4, 142.0, 136.5, 134.8, 130.8, 128.4, 126.4, 124.6, 123.7, 110.7, 110.4, 110.3, 108.4, 105.2, 99.6, 98.7, 97.0, 84.1, 70.1, 69.9, 69.2, 68.3, 40.6, 40.4, 40.2, 40.1.

HRMS (ESI): m/z calc. for $C_{36}H_{39}N_4O_8$ 655.2762; found 655.2801, $[M+H]^+$.

BC1-SS-Biotin: To a solution of **BC1-NH$_2$** (1.30 mg, 2.00 µmol) in anhydrous dimethylformamide (100 µL), NHS-SS-PEG$_4$-biotin (1.80 mg, 2.40 µmol) was added. After stirring for 20 min, triethylamine (364 µg, 3.60 µmol) was added and the mixture was stirred for another 20 min. The product was purified via RP-HPLC.

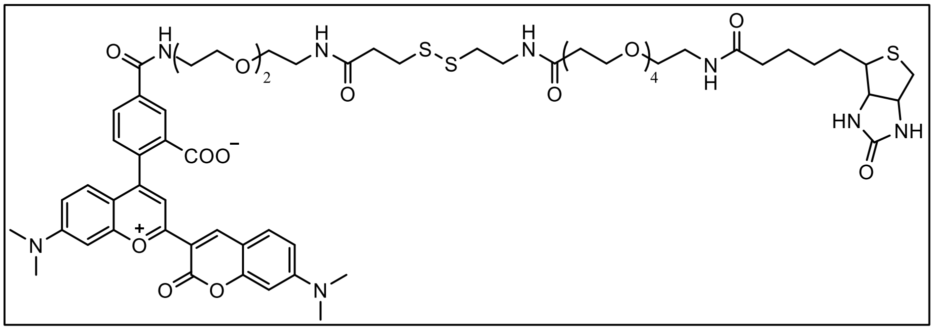

Yield: 246 µg, 0.19 µmol, 10%.

HRMS (ESI): m/z calc. for $C_{62}H_{82}N_8NaO_{16}S_3$ 1313.4903; found 1313.4946, $[M+Na]^+$.

N-(2-(2-(2-Aminoethoxy)ethoxy)ethyl)-2,4-dinitroaniline (7): 1-Fluoro-2,4-dinitrobenzene (205 mg, 1.10 mmol) dissolved in dichloromethane (5 mL) was added dropwise to a solution of 2,2'-(Ethylenedioxy)bisethylenediamine (1.48 g, 10.0 mmol) in dichloromethane (10 mL) at 0 °C. After the addition was complete, the mixture was stirred for 30 minutes at room temperature and then diluted with a mixture of dichloromethane (100 mL) and water (100 mL). The organic phase was separated and mixed thoroughly with 0.1 M hydrochloric acid (50 mL). The aqueous phase was recovered and the pH was adjusted to 12 with sodium hydroxide. The product was extracted into the organic phase again using dichloromethane (3 × 50 mL). The organic phases were combined, washed with brine (100 mL), dried over anhydrous magnesium sulfate, and evaporated to yield pure compound **7**.

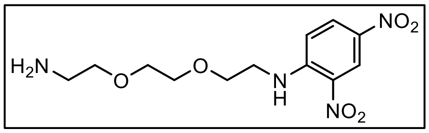

Yield: 310 mg, 0.98 mmol, 92%.

^{1}H NMR (300 MHz, $CDCl_3$) δ 9.12 (d, J = 2.7 Hz, 1H), 8.25 (dd, J = 9.5, 2.7 Hz, 1H), 6.93 (d, J = 9.5 Hz, 1H), 3.83 (dd, J = 5.7, 4.8 Hz, 2H), 3.74 – 3.68 (m, 2H), 3.68 – 3.63 (m, 2H), 3.59 (d, J = 5.1 Hz, 2H), 3.51 (t, J = 5.2 Hz, 2H), 2.86 (t, J = 5.2 Hz, 2H).

^{13}C NMR (75 MHz, $CDCl_3$) δ 148.4, 136.1, 130.5, 130.2, 124.3, 114.0, 73.5, 70.8, 70.3, 68.5, 43.2, 41.7.

HRMS (ESI): m/z calc. for $C_{12}H_{19}N_4O_6$ 315.1299; found 315.1293, $[M+H]^+$.

BC1-DN: Compound **7** (250 mg, 80.0 µmol) was added into a mixture of compound **4** (280 mg, 53.0 µmol), (benzotriazol-1-yloxy)tris(dimethylamino)phosphonium hexafluorophosphate (278 mg, 63.0 µmol) and N,N-diisopropylethylamine (20.0 mg, 155 µmol) in anhydrous dimethylformamide (1 mL). The mixture was stirred for 1 h. Then, the mixture was diluted with water (50 mL) and extracted with ethyl acetate (5 × 50 mL). The combined organic phases were dried over magnesium sulfate and evaporated under reduced pressure. The obtained blue solid was purified by RP-HPLC.

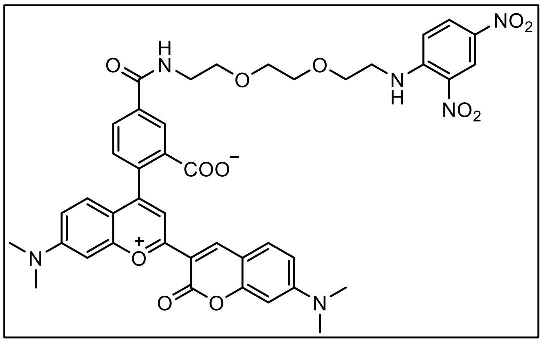

Yield: 5.30 mg, 6.46 µmol, 12%.

^{1}H NMR (400 MHz, CD_3OD) δ 8.93 (s, 1H), 8.87 (d, J = 2.8 Hz, 1H), 8.63 (d, J = 1.7 Hz, 1H), 8.23 (s, 1H), 8.21 – 8.14 (m, 2H), 7.61 (d, *J* = 9.0 Hz, 1H), 7.49 (d, J = 7.9 Hz, 1H), 7.25 – 7.07 (m, 4H), 6.92 (d, J = 9.0 Hz, 1H), 6.60 (s, 1H), 3.83 (t, J = 5.2 Hz, 2H), 3.73 (d, J = 4.8 Hz, 6H), 3.64 (t, J = 5.3 Hz, 2H), 3.60 (t, J = 5.2 Hz, 2H), 3.31 (s, 6H), 3.22 (d, J = 1.8 Hz, 6H).

^{13}**C NMR** (101 MHz, CD_3OD) δ 166.5, 166.5, 162.2, 162.1, 158.5, 158.4, 158.2, 157.3, 157.0, 148.3, 146.1, 139.0, 136.2, 135.6, 132.5, 130.9, 130.6, 130.0, 129.9, 129.7, 129.0, 123.3, 116.9, 116.0, 114.6, 111.9, 111.7, 110.0, 105.8, 96.6, 96.1, 70.2, 70.2, 69.1, 68.4, 42.8, 39.8, 39.7, 39.4.
HRMS (ESI): m/z calc. for $C_{42}H_{41}N_6O_{12}$ 821.2777; found 821.2783, $[M+H]^+$.

2-(4-(dimethylamino)-2-hydroxybenzoyl)benzoic acid (8): 3-Dimethylaminophenol (4.93 g, 36.0 mmol) was dissolved in toluene (10 mL) at 60 ºC in a 100 mL flask. Powdered phthalic anhydride (5.33 g, 36.0 mmol) was added to this solution and the mixture was heated under reflux for 24 h. The solvent was removed under reduced pressure. The residue was purified by column chromatography using cyclohexane:ethyl acetate mixtures (10:0 to 10:2).

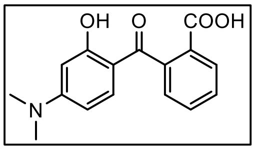

Yield: 560 mg, 1.96 mmol, 6%.
^{1}**H NMR** (300 MHz, $CDCl_3$) δ 8.10 (dd, J = 7.9, 1.2 Hz, 1H), 7.63 (td, J = 7.5, 1.4 Hz, 1H), 7.53 (td, *J* = 7.6, 1.4 Hz, 1H), 7.36 (dd, J = 7.5, 1.3 Hz, 1H), 6.89 (d, J = 9.1 Hz, 1H), 6.16 (d, J = 2.5 Hz, 1H), 6.07 (dd, J = 9.1, 2.5 Hz, 1H), 3.03 (s, 6H).
^{13}**C NMR** (75 MHz, $CDCl_3$) δ 198.6, 169.8, 165.2, 156.0, 141.1, 134.3, 132.8, 131.1, 129.2, 128.0, 127.6, 110.3, 104.0, 97.8, 40.0.
HRMS (ESI): m/z calc. for $C_{16}H_{16}NO_4$ 286.1074; found 286.1075, $[M+H]^+$.

BC1: Compounds **8** (456 mg, 1.60 mmol) and **3** (360 mg, 1.60 mmol) were dissolved in concentrated sulfuric acid (8 mL) and stirred for 6 h at 90 ºC. The mixture was cooled down to room temperature and ice (approx. 15 g) was carefully added. After adding 70% perchloric acid (0.70 mL), the mixture was filtered, and the precipitate was washed with water. The crude product was air-dried overnight and further purified by column chromatography using dichloromethane:methanol mixtures (50:1 to 20:1).

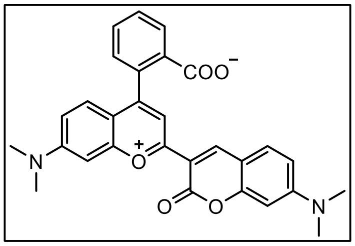

Yield: 477 mg, 1.00 mmol, 60%.
^{1}**H NMR** (500 MHz, C_2D_6OS) δ 9.10 (s, 2H), 8.13 (d, *J* = 7.8 Hz, 1H), 7.84 (td, *J* = 7.6, 1.3 Hz, 1H), 7.77 (t, *J* = 7.6 Hz, 1H), 7.73 (d, *J* = 9.1 Hz, 1H), 7.49 (d, *J* = 7.6 Hz, 1H), 7.20 (s, 3H), 6.98 (dd, *J* = 9.1, 2.4 Hz, 1H), 6.71 (d, *J* = 2.4 Hz, 1H), 3.26 (s, 6H), 3.19 (s, 6H).
^{13}**C NMR** (75 MHz, C_2D_6OS) δ 169.2, 158.9, 156.1, 154.3, 153.8, 152.2, 152.2, 151.5, 147.2, 141.9, 135.7, 130.8, 130.3, 128.8, 128.2, 127.3, 126.0, 125.0, 124.3, 110.6, 110.4, 108.4, 105.7, 98.6, 97.0, 40.3, 40.2.
HRMS (ESI): m/z calc. for $C_{29}H_{25}N_2O_5$ 481.1758; found 481.1747, $[M+H]^+$.

2-fluoro-5-nitro-N,N-dimethylaniline (9): 3 M Sulfuric acid (16.0 mL, 48.0 mmol) and 37% formaldehyde (19.0 mL, 234 mmol) were added into tetrahydrofuran (150 mL). A suspension of 2-fluoro-5-nitroaniline (9.98 g, 64.0 mmol) and sodium borohydride (14.0 g 370 mmol) in 150 mL tetrahydrofuran was added to the above solution in small portions. The reaction mixture was acidified with 3 M sulfuric acid (16.0 mL, 48.0 mmol) again when half of the suspension was added. After 15 min stirring, potassium hydroxide (1 M) was added to the reaction mixture until pH 10 was reached and the organic phase was separated. The aqueous phase was extracted with ethyl acetate (3 × 150 mL). The combined organic phases were washed with brine (2 × 200 mL), dried over magnesium sulfate, and evaporated under reduced pressure. Further purification was carried out by column chromatography using cyclohexane:ethyl acetate mixtures (10:0 to 10:0.5).

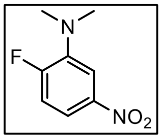

Yield: 3.10 g, 16.8 mmol, 26%.

^{1}H NMR (300 MHz, $CDCl_3$) δ 7.70 (ddd, *J* = 6.9, 2.9, 0.9 Hz, 2H), 7.08 (ddd, *J* = 12.3, 9.8, 0.7 Hz, 1H), 2.94 (d, *J* = 1.3 Hz, 6H).

^{13}C NMR (75 MHz, $CDCl_3$) δ 157.9 (d, *J* = 256.3 Hz), 144.6, 141.2 (d, *J* = 10.0 Hz), 116.5 (d, *J* = 24.2 Hz), 115.7 (d, *J* = 9.4 Hz), 113.0 (d, *J* = 6.2 Hz), 42.3, 42.3.

HRMS (ESI): m/z calc. for $C_8H_{10}FN_2O_2$ 185.0721; found 185.0723, $[M+H]^+$.

5-amino-2-fluoro-N,N-dimethylaniline (10): To a Schlenk flask filled with argon, 10% palladium on carbon (90 mg) and ethyl acetate (4 mL) were added. The argon was exchanged by hydrogen gas and the mixture was stirred vigorously for 15 min at room temperature to activate the catalyst. Compound **9** (595 mg, 3.20 mmol) in ethyl acetate (3 mL) was added to the solution and stirred for 4 h at room temperature. After the reaction was completed, the hydrogen was replaced with argon. The mixture was filtrated through Celite, and the filter cake was washed thoroughly with ethyl acetate. The solvent was evaporated under reduced pressure to yield pure **10**.

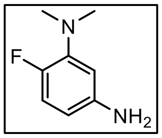

Yield: 498 mg, 3.23 mmol, quantitative.

^{1}H NMR (300 MHz, $CDCl_3$) δ 6.77 (ddd, *J* = 15.8, 12.1, 8.5 Hz, 1H), 6.33 – 5.78 (m, 2H), 2.94 – 2.67 (m, 6H).

^{13}C NMR (75 MHz, $CDCl_3$) δ 149.0 (d, *J* = 235.4 Hz), 142.7 (d, *J* = 2.2 Hz), 141.1 (d, *J* = 9.9 Hz), 116.3 (d, *J* = 22.3 Hz), 107.0 (d, *J* = 7.5 Hz), 105.5 (d, *J* = 2.8 Hz), 42.8, 42.7.

HRMS (ESI): m/z calc. for $C_8H_{12}FN_2$ 155.0979; found 155.0981, $[M+H]^+$.

2-fluoro-5-hydroxy-N,N-dimethylaniline (11): Compound **10** (2.80 g, 18.0 mmol) was dissolved in 35% sulfuric acid (22 mL) and the temperature was decreased to 0 °C. Sodium nitrite (1.52 g, 22.0 mmol) dissolved in water (16 mL) was added and the reaction mixture was stirred for 30 min. Following addition of several crystals of urea, the mixture was poured into a solution of copper (II) nitrate trihydrate (60.0 g, 248 mmol) in water (600 mL). After stirring for several minutes, copper (I) oxide (2.15 g, 15.0 mmol) was added in small portions. The mixture was stirred vigorously for 1 h. Then, a saturated solution of sodium bicarbonate was added until pH 8 was reached. The suspension was filtered, and the precipitate was washed thoroughly with ethyl acetate. The filtered solution was extracted with ethyl acetate (3 × 200 mL) The combined organic phases were washed with brine, dried over magnesium sulfate and evaporated under reduced pressure. The crude product was purified via column chromatography using cyclohexane:ethyl acetate mixtures (10:0 to 10:3).

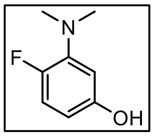

Yield: 1.70 g, 11.0 mmol, 61%.

^{1}H NMR (300 MHz, $CDCl_3$) δ 6.85 (dd, *J* = 12.7, 8.6 Hz, 1H), 6.38 (dd, *J* = 7.4, 3.0 Hz, 1H), 6.26 (dt, *J* = 8.6, 3.1 Hz, 1H), 2.82 (d, *J* = 0.9 Hz, 6H).

^{13}C NMR (75 MHz, $CDCl_3$) δ 151.8, 149.6 (d, *J* = 237.0 Hz), 141.4 (d, *J* = 10.2 Hz), 116.3 (d, *J* = 22.9 Hz), 106.4 (d, *J* = 7.8 Hz), 105.5 (d, *J* = 3.4 Hz), 42.7, 42.6.

HRMS (ESI): m/z calc. for $C_8H_{11}FNO$ 156.0819; found 156.0821, $[M+H]^+$.

2-(4-(dimethylamino)-5-fluoro-2-hydroxybenzoyl)benzoic acid (12): A mixture of powdered phthalic anhydride (296 mg, 2.00 mmol) and compound **11** (155 mg, 1.00 mmol) was heated at 150 °C under argon. After 20 min, zinc chloride (136 mg, 1.00 mmol) was added and the reaction mixture was heated up to 180 °C for 6 h. After cooling to room temperature, water (10 mL) was added. The suspension was filtered, and the precipitate was washed twice with cold-water. A dark violet solid was obtained. The solid was dissolved in 35% sodium hydroxide (5 mL) and stirred at 90 °C for 8 h under argon. After the reaction was complete, the mixture was neutralized with hydrochloric acid (conc.) to pH 6 in an ice bath. A dark pink precipitate was formed and collected via filtration. The crude product was purified by column chromatography with dichloromethane:methanol mixtures (10:0 to 10:1).

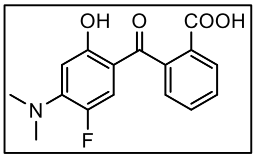

Yield: 69.0 mg, 227 µmol, 23%.

^{1}H NMR (500 MHz, $CDCl_3$) δ 8.13 (dd, *J* = 7.9, 1.3 Hz, 1H), 7.67 (td, *J* = 7.5, 1.3 Hz, 1H), 7.58 (td, *J* = 7.7, 1.3 Hz, 1H), 7.36 (dd, *J* = 7.6, 1.3 Hz, 1H), 6.61 (d, *J* = 14.8 Hz, 1H), 6.26 (d, *J* = 7.8 Hz, 1H), 3.04 (d, *J* = 2.0 Hz, 6H).

^{13}C NMR (126 MHz, $CDCl_3$) δ 198.8 (d, *J* = 2.4 Hz), 168.7, 160.8, 147.3 (d, *J* = 9.6 Hz), 145.5 (d, *J* = 236.8 Hz), 140.7, 132.9, 131.1, 129.5, 127.6, 127.6, 118.2 (d, *J* = 23.8 Hz), 109.8 (d, *J* = 6.5 Hz), 102.9 (d, *J* = 3.7 Hz), 42.1, 42.0.
HRMS (ESI): m/z calc. for $C_{16}H_{14}FNNaO_4$ 326.0799; found 326.0808, $[M+Na]^+$.

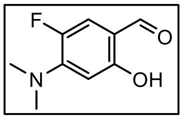

5-fluoro-4-(dimethylamino)-2-hydroxybenzaldehyde (13): Phosphorus(V) oxychloride (1.53 g, 10.0 mmol) was added dropwise into anhydrous dimethylformamide (15 mL) at 0 ºC under argon. Compound **11** (605 mg, 3.90 mmol) in dimethylformamide (10 mL) was slowly added into this solution. The reaction mixture was brought to room temperature, stirred for 30 min, and then heated to 75 °C, and stirred for 2 h. After the reaction was complete, ice-cold water (30 mL) was added. The mixture was neutralized with sodium acetate and extracted with ethyl acetate (4 × 100 mL). The combined organic phases were washed with brine, dried over magnesium sulfate, and evaporated. The crude product was purified by column chromatography using cyclohexane:ethyl acetate mixtures (10:0 to 10:2)
Yield: 249 mg, 1.36 mmol, 36%.
^{1}H NMR (300 MHz, $CDCl_3$) δ 7.04 (d, *J* = 13.8 Hz, 1H), 6.17 (d, *J* = 7.5 Hz, 1H), 3.07 (d, *J* = 2.2 Hz, 6H).
^{13}C NMR (75 MHz, $CDCl_3$) δ 192.0 (d, *J* = 2.3 Hz), 160.1, 147.5 (d, *J* = 9.4 Hz), 146.2 (d, *J* = 237.5 Hz), 118.6 (d, *J* = 23.0 Hz), 110.7 (d, *J* = 6.2 Hz), 102.2 (d, *J* = 3.7 Hz), 42.1, 42.0.
HRMS (ESI): m/z calc. for $C_9H_{10}FNNaO_2$ 206.0588; found 206.0592, $[M+Na]^+$.

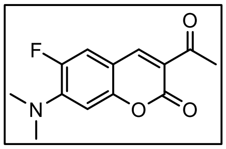

3-acetyl-7-(dimethylamino)-6-fluoro-2H-chromen-2-one (14): Compound **13** (238 mg, 1.30 mmol) and ethyl acetoacetate (182 mg, 1.40 mmol) were dissolved in absolute ethanol (3 mL). A few drops of piperidine were slowly added to the solution and the mixture was refluxed for 4 h. The reaction mixture was kept at 4 ºC overnight, leading to formation of a yellow precipitate which was filtered off and washed with ice-cold ethanol. The crude product was purified with column chromatography using cyclohexane:ethyl acetate mixtures (10:0.5 to 10:2).
Yield: 241 mg, 0.97 mmol, 77%.
^{1}H NMR (500 MHz, $CDCl_3$) δ 8.39 (s, 1H), 7.16 (d, *J* = 13.4 Hz, 1H), 6.58 (d, *J* = 7.6 Hz, 1H), 3.13 (d, *J* = 2.2 Hz, 7H), 2.69 (s, 3H).
^{13}C NMR (126 MHz, $CDCl_3$) δ 195.5, 160.2, 154.2, 149.8 (d, *J* = 244.4 Hz), 147.0 (d, *J* = 3.1 Hz), 145.8 (d, *J* = 10.1 Hz), 118.9, 115.5 (d, *J* = 24.1 Hz), 108.6 (d, *J* = 9.4 Hz), 102.1 (d, *J* = 4.6 Hz), 42.3, 42.3, 30.6.

HRMS (ESI): m/z calc. for $C_{13}H_{12}FNNaO_3$ 272.0693; found 272.0702, $[M+Na]^+$.

General method of the condensation reaction of benzoic acid and coumarin to BC2, BC3, and BC4: Compounds **12** (30.3 mg, 100 μmol) and **3** (23.0 mg, 100 μmol) for **BC2**, compounds **8** (28.5 mg, 100 μmol) and **14** (24.9 mg, 100 μmol) for **BC3**, compounds **12** (29.0 mg, 100 μmol) and **14** (24.9 mg, 100 μmol) for **BC3** were dissolved in concentrated sulfuric acid (2 mL) and stirred at 90 ºC for 24 h under argon. The mixture was cooled down to room temperature and crushed ice (approx. 10 g) was added. After adding 70% perchloric acid (2 drops), the mixture was filtered. The precipitate was washed with water, dried in air overnight and purified by RP-HPLC.

BC2:

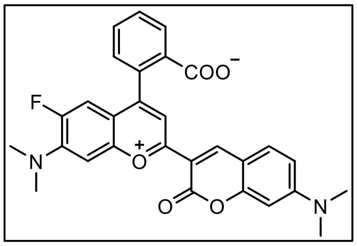

Yield: 7.30 mg, 14.7 μmol, 15%.

^{1}H NMR (500 MHz, $CDCl_3$) δ 8.41 (s, 1H), 8.03 (d, *J* = 7.7 Hz, 1H), 7.67 (td, *J* = 7.5, 1.2 Hz, 1H), 7.60 (td, *J* = 7.5, 1.1 Hz, 1H), 7.49 (d, *J* = 8.9 Hz, 1H), 7.27 (d, *J* = 5.9 Hz, 2H), 6.80 (d, *J* = 7.6 Hz, 2H), 6.69 (dd, *J* = 8.9, 2.5 Hz, 1H), 6.49 (d, *J* = 2.4 Hz, 1H), 6.39 (d, *J* = 13.3 Hz, 1H), 3.12 (s, 6H), 3.00 – 2.93 (m, 6H).

^{13}C NMR (126 MHz, $CDCl_3$) δ 169.2, 158.9, 156.3, 154.0, 152.1, 150.2, 134.4, 130.2 (d, *J* = 3.1 Hz), 129.7, 126.9, 124.5 (d, *J* = 5.4 Hz), 113.5 (d, *J* = 23.9 Hz), 110.0, 108.8, 104.6, 97.1, 42.5 (d, *J* = 5.4 Hz), 40.3.

HRMS (ESI): m/z calc. for $C_{29}H_{24}FN_2O_5$ 499.1664; found 499.1662, $[M+H]^+$.

BC3:

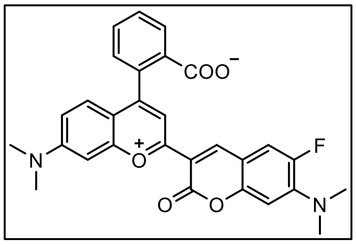

Yield: 7.20 mg, 14.5 μmol, 10%.

^{1}H NMR (500 MHz, $CDCl_3$) δ 8.36 (s, 1H), 7.98 (dd, *J* = 7.5, 1.0 Hz, 1H), 7.64 (td, *J* = 7.5, 1.2 Hz, 1H), 7.57 (td, *J* = 7.5, 1.0 Hz, 1H), 7.27 – 7.22 (m, 2H), 6.73 (s, 1H), 6.63 (dd, *J* = 12.9, 8.2 Hz, 2H), 6.55 (d, *J* = 2.5 Hz, 1H), 6.43 (dd, *J* = 8.9, 2.6 Hz, 1H), 3.07 (d, *J* = 1.9 Hz, 6H), 3.01 (s, 6H).

^{13}C NMR (126 MHz, $CDCl_3$) δ 169.6, 158.5, 152.6, 152.1, 151.4 (d, *J* = 55.1 Hz), 149.3, 144.6 (d, *J* = 10.2 Hz), 139.6, 134.4, 129.4, 128.4, 126.8, 125.3, 124.1, 114.3 (d, *J* = 24.4 Hz), 110.0, 109.6, 109.5, 102.8, 102.8, 98.3, 42.3, 42.2, 40.3.

HRMS (ESI): m/z calc. for $C_{29}H_{24}FN_2O_5$ 499.1664; found 499.1653, $[M+H]^+$.

BC4:

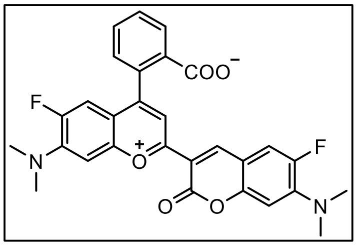

Yield: 5.70 mg, 11.0 µmol, 11%.

^{1}H NMR (300 MHz, $CDCl_3$) δ 8.37 (s, 1H), 8.00 (dt, J = 7.4, 1.1 Hz, 1H), 7.62 (dtd, J = 22.6, 7.4, 1.2 Hz, 2H), 7.26 (d, *J* = 2.1 Hz, 1H), 7.25 – 7.21 (m, 1H), 6.78 (d, J = 7.5 Hz, 2H), 6.60 (d, J = 7.5 Hz, 1H), 6.36 (d, J = 13.3 Hz, 1H), 3.07 (d, J = 1.9 Hz, 7H), 2.95 (d, J = 1.3 Hz, 6H).

^{13}C NMR (126 MHz, $CDCl_3$) δ 169.1, 158.4, 152.2, 151.9, 151.1, 150.2, 149.2, 144.8 (d, *J* = 10.6 Hz), 134.5, 129.8, 126.7, 124.3, 114.5 (d, *J* = 24.8 Hz), 113.5 (d, *J* = 23.7 Hz), 109.4, 104.6, 102.6 (d, *J* = 3.3 Hz), 42.5, 42.5, 42.3, 42.3.

HRMS (ESI): m/z calc. for $C_{29}H_{23}F_2N_2O_5$ 517.1570; found 517.1570, $[M+H]^+$.

General method for the amidation of 5-carboxyl BC1 to BC5, BC6, and BC7: To a solution of **BC1** (23.3 mg, 50.0 µmol) in anhydrous dichlormethane (3 mL), phosphorus(V) oxychloride (61.0 mg, 400 µmol) was added dropwise. The mixture was refluxed for 4 h. After cooling, the reaction mixture was evaporated under reduced pressure to obtain the crude acid chloride. An ammonia solution (conc. 300 µL) for **BC5** or cyanamide (42.0 mg, 1.00 mmol) for **BC6** or methanesulfonamide (95.1 mg, 1.00 mmol) for **BC7** and triethylamine (40.0 mg, 396 µmol) in acetonitrile (2 mL) was added to the acid chloride. The reaction mixture was stirred at room temperature. After 2 h, water was added to the reaction mixture, and the aqueous phase was extracted with dichloromethane. The combined organic layers were dried over magnesium sulfate and purified with RP-HPLC.

BC5:

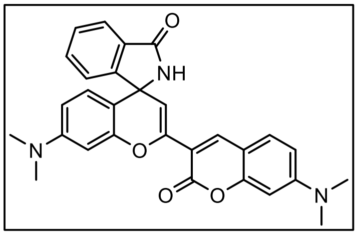

Yield: 4.30 mg, 8.98 µmol, 18%.

^{1}H NMR (400 MHz, $CDCl_3$) δ 8.32 (s, 1H), 7.89 (d, *J* = 7.6 Hz, 1H), 7.54 – 7.43 (m, 3H), 7.21 (d, *J* = 7.2 Hz, 1H), 6.74 – 6.67 (m, 2H), 6.52 (dd, J = 12.7, 2.5 Hz, 2H), 6.44 (d, J = 8.6 Hz, 2H), 3.11 (s, 6H), 3.00 (s, 6H).

^{13}C NMR (101 MHz, $CDCl_3$) δ 169.3, 159.2, 155.8, 154.3, 154.3, 153.4, 151.7, 151.2, 145.0, 139.4, 132.8, 130.1, 129.5, 128.4, 128.2, 124.0, 123.3, 112.7, 109.6, 108.7, 107.7, 103.3, 98.8, 97.3, 58.3, 40.4, 40.2.

HRMS (ESI): m/z calc. for $C_{29}H_{26}N_3O_4$ 480.1918; found 480.1911, $[M+H]^+$.

BC6:

Yield: 3.80 mg, 7.54 µmol, 14%.

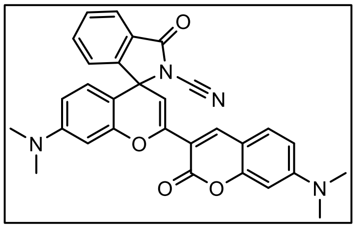

^{1}H NMR (400 MHz, $CDCl_3$) δ 8.31 (s, 1H), 7.89 (d, J = 7.7 Hz, 1H), 7.60 (td, J = 7.5, 1.2 Hz, 1H), 7.49 (t, *J* = 7.5 Hz, 1H), 7.39 (d, J = 8.8 Hz, 1H), 7.22 (d, J = 7.7 Hz, 1H), 6.61 (dd, J = 8.8, 2.4 Hz, 1H), 6.50 – 6.42 (m, 4H), 6.34 (dd, J = 8.9, 2.5 Hz, 1H), 3.03 (s, 6H), 2.92 (s, 6H).

^{13}C NMR (101 MHz, $CDCl_3$) δ 166.1, 159.2, 156.0, 153.7, 152.6, 151.9, 151.8, 147.9, 140.8, 135.4, 129.9, 129.6, 127.8, 126.4, 124.9, 124.6, 111.6, 110.1, 109.8, 108.6, 107.1, 103.7, 98.8, 98.7,

HRMS (ESI): m/z calc. for $C_{30}H_{24}N_4NaO_4$ 527.1690; found 527.1682, $[M+Na]^+$.

BC7:

Yield: 5.80 mg, 10.0 µmol, 20%.

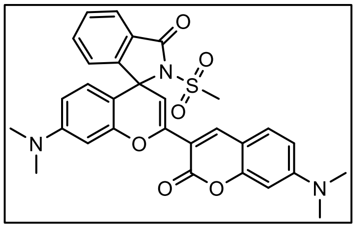

^{1}H NMR (400 MHz, $CDCl_3$) δ 8.31 (s, 1H), 7.88 (d, J = 7.7 Hz, 1H), 7.55 (td, J = 7.5, 1.2 Hz, 1H), 7.45 (td, J = 7.5, 1.0 Hz, 1H), 7.38 (d, J = 8.8 Hz, 1H), 7.17 (d, J = 7.7 Hz, 1H), 6.60 (dd, J = 8.9, 2.4 Hz, 1H), 6.54 (d, J = 8.8 Hz, 1H), 6.44 (dd, J = 7.1, 2.4 Hz, 2H), 6.39 (s, 1H), 6.32 (dd, J = 8.8, 2.5 Hz, 1H), 3.14 (s, 3H), 3.03 (s, 6H), 2.91 (s, 6H).

^{13}C NMR (101 MHz, $CDCl_3$) δ 167.0, 159.6, 155.9, 153.5, 152.8, 152.6, 151.4, 146.8, 140.4, 134.9, 129.7, 129.2, 127.7, 127.5, 124.7, 124.2, 112.3, 109.7, 109.3, 108.8, 106.6, 100.4, 98.7, 97.2, 68.2, 42.5, 40.3, 40.2.

HRMS (ESI): m/z calc. for $C_{30}H_{27}N_3NaO_6S$ 580.1513; found 580.1510, $[M+Na]^+$.

4. Selection, Truncation and Mutation Study

4.1 DNA library preparation

The DNA library contains a T7 promoter sequence, two fixed primer binding sites, two 26-nucleotide (nt) random regions (indicated with N) interspersed by a 12-nt constant region. A reverse complementary ssDNA library was ordered from IDT. The phosphoramidites used for the synthesis of the random regions were hand-mixed in an A:C:G:T ratio of 3:3:2:2. The dsDNA library (3 × 10^{15} molecules, 320 nM) was prepared via PCR amplification (6-cycles) using the ssDNA as templates. PCR conditions: primer A (10 µM), primer B (10 µM), dNTPs (1 mM), $MgCl_2$ (6 mM), Taq DNA polymerase, and PCR buffer (20 mM Tris HCl, 50 mM KCl, pH 8.4). After phenol:chloroform:isoamyl alcohol (PCI; 25:24:1) extraction, the PCR product was precipitated with sodium acetate (0.3 M) and isopropanol. The DNA pellet was dissolved in Millipore water and used for *in vitro* transcription of the RNA library.

DNA library design (122-nt)	TCTAATACGACTCACTATAGGAGCTCAGCCTTCACTGCNNNNNNNNNNNNNNNNNNNNNNNNNNCTGCTTCGGCAGNNNNNNNNNNNNNNNNNNNNNNNNNNGGCACCACGGTCGGATCCAC<br>(Underline: T7 promoter; Green: primer binding site; Blue: inserted constant region)
ssDNA template (99-nt)	GATCCGACCGTGGTGCCNNNNNNNNNNNNNNNNNNNNNNNNNCTGCCGAAGCAGNNNNNNNNNNNNNNNNNNNNNNNNNNGCAGTGAAGGCTGAGTCC
Forward primer (primer A)	TCTAATACGACTCACTATAGGAGCTCAGCCTTCACTGC
Reverse primer (primer B)	GTGGATCCGACCGTGGTGCC

4.2 Resin preparation

Preparation of the sepharose resin functionalized with BC1-NH$_2$ for rounds 1 – 5. 4 mL of NHS-activated sepharose 4 Fast Flow (GE Healthcare Life Sciences) resin was transferred into a 15 mL falcon and washed with 1 mM hydrochloric acid (2 × 8 mL, ice-cold). The resin was resuspended in 100 mM Hepes buffer (1 mL, pH 7.4). **BC1-NH$_2$** (11 mg, 17 μmol) was dissolved in 100 μL DMSO and further diluted with 1 mL Hepes buffer. The dye solution was added to the resin on ice and mixed thoroughly. The suspension was kept at 25 °C overnight with vigorous shaking. Next day, the resin was further incubated with ethanolamine (0.2 M, 12 μL) at 25 °C for 2 h to quench any unreacted NHS groups. The resin was washed with 50 mM Hepes buffer (6 × 8 mL) until the supernatant became colorless. The resin was washed once more with sodium acetate (0.1 M, pH 5.5,) and stored in sodium acetate containing 20% ethanol at 4 °C. The coupling efficiency of **BC1-NH$_2$** was determined to be 4 mM by measuring the absorption, thereby the concentration of the dye before reaction (total dye) and after reaction in wash solutions (unreacted dye). The mock resin for negative selection was prepared analogously without adding **BC1-NH$_2$**.

Preparation of the streptavidin-agarose resin functionalized with BC1-SS-Biotin for rounds 6 – 11.

In order to easily control the **BC1** concentration on beads, the resin for selection was switched to streptavidin-functionalized agarose resin. **BC1-SS-Biotin** (7.5 mM in DMSO) was diluted with 200 μL of 1 × aptamer selection buffer (1 × ASB: 20 mM Hepes, 5 mM $MgCl_2$, 125 mM KCl, pH 7.4) and loaded to streptavidin resin in portions with vigorous shaking. After incubating for 5 min, the beads were washed 6 times with 1 × ASB (1 mL). For negative selection, the streptavidin beads were used directly without functionalization.

4.3 Selection protocol

1) Large scale RNA preparation: The RNA library for the first selection round was prepared via large scale *in vitro* transcription reaction (10 mL) containing: the double-stranded (ds) DNA template (1.5 µM), DTT (10 mM), NTPs (4 mM of each ATP, CTP, GTP, UTP), T7 RNA polymerase, and transcription buffer (40 mM Tris pH 8.1, 1 mM spermidine, 22 mM $MgCl_2$, 0.01% Triton-X-100). After incubation for 4 h at 37 °C, DNaseI (1 U/µL) was added and incubated for another 30 min. The transcription mixture was extracted with PCI (25:24:1, pH 4.5–5) and precipitated with isopropanol. The pellet was washed twice with 75% ethanol and dissolved in Millipore water. **2) RNA folding:** The desired amount of RNA (round 1: 100 µM, round 2: 50 µM, round 3–11: 10 µM) was dissolved in Millipore water, incubated at 75 °C for 2 min, followed by slow cooling to 25 °C. 1/5 volume of 6 × ASB was added to the solution and the mixture was kept at 25 °C for 15 min. **3) Mock selection:** The folded RNA was first incubated with mock resin (round 1: 500 µL, round 2-11: 250 µL) at 25 °C for 1 h to remove resin-binding RNA. **4) RNA binding:** The remaining unbound RNA were incubated with the **BC1** conjugated resin at 25 °C for 30 min. The resin was then washed with 1 × ASB (round 1: 6 column volumes (cv), round 2–5: 12 cv, round 6–11: 30 cv). **5) RNA elution:** The bound RNA was eluted with 98% formamide containing 10 mM EDTA at 65 °C for 5 min (round 1–5) or 50 mM DTT at 25 °C for 10 min (round 6–11). The eluted RNA was co-precipitated with glycogen using ethanol. The RNA pellet was washed two times with 75% ethanol and dissolved in Millipore water. **6) Reverse transcription and PCR amplification:** The RNA was reverse transcribed (RT) by SuperScript™ IV reverse transcriptase (Invitrogen) according to the manufacturer's instruction (200 µL of total volume). The RT reaction was immediately used for PCR amplification (800 µL, 10-cycles). The RT-PCR products were purified using a PCR purification kit (Qiagen). **7) RNA *in vitro* transcription:** RNA was transcribed from the DNA template (reaction volume for rounds 2–11: 800 µL), purified by 10% denaturing polyacrylamide gel electrophoresis (PAGE) and used as input RNA for the next selection round. The selection was carried out for 11 rounds using the described protocol. The selection was monitored by calculating the fraction of eluted RNA (Number of eluted RNA molecules / Number of RNA molecules used as input) based on UV-spectrometric measurements (Nanodrop).

4.4 Sanger sequencing

The RNA eluted from selection round 11 was reverse transcribed and PCR-amplified using the standard protocol described above (section 4.3). The purified DNA product was phosphorylated

(Fast DNA End Repair Kit, Thermo Fisher Scientific) and ligated into pDisplay-AP-CFP-TM plasmid (Addgene, plasmid #20861) which had already been digested with EcoRV and dephosphorylated. The vectors were transformed into competent DH5α cells. 96 colonies were picked and sent for Sanger sequencing.

4.5 Activity screening of sequenced RNA pool

All 52 unique sequences were PCR-amplified, *in vitro* transcribed individually, and PAGE-purified using the standard protocol described above (section 4.1 and 4.3). The folded RNA (10 µM) were incubated with **BC1-DN** (100 nM, diluted from 10 µM stock solution in DMSO) and directly used for the fluorescence measurement. Emission spectra were recorded using an excitation wavelength of 652 nm (± 5 nm slit width). The fluorescence turn-on fold was determined by dividing the maximal fluorescence intensity of **BC1-DN** in the presence of RNA aptamer (10 µM) to that of **BC1-DN** in buffer solution (1 × ASB) alone.

4.6 Sequence alignment

Sequence alignment of all 52 unique sequences was conducted using MultAlin[1]. Symbol comparison table: blosum62; Gap weight: 12; Gap length weight: 2.

4.7 Dissociation constant measurement of active sequences

The RNA (25 µM) was folded and diluted to the desired concentrations with 1 × ASB (0, 0.01, 0.1, 0.25, 0.5, 1, 2.5, 5, 10, 25 µM). The fluorescence of **BC1-DN** (100 nM) was measured as a function of RNA concentration. The excitation was 652 nm (± 5 nm slit width). The maximal fluorescence intensity was recorded and plotted as a function of RNA concentration. All the curves were fitted and the dissociation constants (K_D) was determined using Hill1 equation in OriginPro 2015.

4.8 Truncation analysis of RNA8

T7 RNA polymerase was used to *in vitro* transcribe RNA8-1, RNA8-2, RNA8-3 from their corresponding dsDNA templates, produced via PCR using region-specific primers. The crude RNA was PAGE-purified, folded, incubated with **BC1-DN** (100 nM), and subjected to a fluorescence assay. The emission spectra were recorded at 652 nm excitation (± 5 nm slit width). The fluorescence turn-on values were determined by dividing the maximal fluorescence intensities of **BC1-DN** in the presence of RNA aptamer (10 µM) by that of **BC1-DN** in buffer solution (1 × ASB) alone. The fluorescence intensities at emission maximum (690 nm) were

plotted and fitted using least-square fitting according to the given equation, and the K_D values were determined (OriginPro 2015).

$$F = F_0 + \frac{(F_\infty - F_0) \times \left\{(K_D + P_0 + [Apt]) - \sqrt{([Apt] - P_0)^2 + K_D \times (K_D + 2[Apt] + 2P_0)}\right\}}{2P_0}$$

F: fluorescence intensity at any given RNA concentration; F_0: fluorescence of a free probe with an initial concentration of P_0; F_∞: maximum fluorescence of the complex; $[Apt]$: concentration of added RNA; K_D: dissociation constant.

4.9 Mutant activity screening

All DNA mutant templates were ordered from IDT. M1–12 were prepared via PCR-amplification, *in vitro* transcription, and PAGE purification. K_D values and fluorescence turn-on folds for each complex were determined as described above (section 4.8).

5. Substrate Screening for BeCA

5.1 Absorbance and emssion spectra of BC 1–7

BC 1–7 (1 μM) in 20 mM Hepes buffer containing 0.1% Triton X-100 were prepared. The absorbance spectra of **BC 1–7** were recorded individually. For the emission spectra of both spirocyclic and zwitterionic form, **BC 1–7** were excited at their excitation maxima (± 5 nm slit width).

5.2 Absorbance spectra of BC 1–7 at various dielectric constants

Solutions of **BC 1–7** (5 μM) in water-dioxane mixtures containing 10%, 20%, 30%, 40%, 50%, 60%, 70%, 80% and 90% of dioxane (by volume) were prepared and the absorbance spectra were recorded. The absorbance of the zwitterionic form of **BC 1–7** at each dielectric constant condition was normalized to that of each probe in water containing 0.3% sodium dodecyl sulfate (SDS) in which the fluorophores were predominantly converted to their corresponding zwitterions. One exception was **BC5,** which predominately existed as a spirolactam in water and could not be converted to its corresponding zwitterion by SDS. Thus, the absorbance of the **BC5** zwitterion at different dielectric constants was normalized to that of its parent structure **BC1** in water containing 0.3% SDS. The normalized absorbance of **BC 1–7** was plotted against dielectric constants of water-dioxane mixtures[2].

5.3 Fluorescence light-up studies of BC 1–7 upon binding BeCA

BeCA (10 μM) was folded in 1 × ASB and incubated with **BC 1–7** (100 nM), respectively. Emission spectra were recorded using the excitation maxima (± 5 nm slit width) of each probe. Near-infrared (NIR) fluorescence turn-on factors were determined by dividing the maximal fluorescence intensities of **BC 1–7** in the presence of **BeCA** (10 μM) by those of fluorophores alone.

6. Characterization of BeCA-BC6

6.1 Absorption, excitation and emission spectra of BeCA-BC6

The absorption, excitation and emission spectra of **BC6** (1 μM) in the presence of **BeCA** (25 μM) or **BC6** (1 μM) in buffer alone were recorded. Similarly, the maximal fluorescence intensities of cyan and NIR channels were recorded by titrating **BC6** (100 nM) with different **BeCA** concentrations (0, 0.005, 0.01, 0.05, 0.025 0.1, 0.25, 0.5, 1, 2.5, 5, 10, 25 μM) and the ratios between cyan/NIR were plotted as a function of **BeCA** concentration. The excitation wavelengths used for measurements were 420 nm (± 5 nm slit width) and 630 nm (± 5 nm slit width) respectively. Measurements were performed in 1 × ASB containing 0.05% Triton X-100.

6.2 Dissociation constant measurement of BeCA-BC6

The spectra were recorded and K_D values were determined using the method described above (section 4.8). An excitation wavelength of 665 nm (± 5 nm slit width) was used.

6.3 Temperature dependence of BeCA-BC6

BeCA (5 μM) in 1 × ASB was prepared and incubated with **BC6** (5 μM). The fluorescence intensity was recorded upon increasing the temperature from 20 °C to 70 °C (2 °C interval in 2 min). The excitation was 665 nm (± 5 nm slit width).

6.4 Magnesium and potassium concentration dependence of BeCA-BC6

BeCA (5 μM) was folded in 1 × ASB containing different amounts of $MgCl_2$ (0, 10, 25, 50, 100, 250, 500, 1000, 2500, 5000, 10000 μM) or KCl (0, 1, 2.5, 5, 10, 25, 50, 100, 200, 300 mM). Emission spectra were recorded upon addition of **BC6** (5 μM) using an excitation wavelength of 665 nm (± 5 nm slit width) and the fluorescence intensities at emission maximum (684 nm) were plotted against the magnesium/potassium concentration.

6.5 Photophysical properties of BC6 and BeCA-BC6

The absorbance and emission spectra of free **BC6** (0.2, 0.4, 0.6, 0.8, 1 μM) or the **BeCA-BC6** complex prepared by mixing varying concentrations of **BC6** (0.2, 0.4, 0.6, 0.8, 1 μM) with excess

amounts of **BeCA** (25 µM) in 1 × ASB containing 0.05% Triton X-100 were recorded**.** The excitation wavelengths for the spirolactam and zwitterion were 420 nm (± 3 nm slit width) and 615 nm (± 3 nm slit width), respectively. The integrated areas under the fluorescence emission spectra were calculated and plotted against the absorbance of the fluorophores at the excitation wavelength. Coumarin 153 and oxazine 1 in ethanol were used as standard references for the two channels, respectively[3]. The slopes were determined using linear fit and the quantum yields were calculated using the following equation:

$$\emptyset = \emptyset_R \times \frac{S}{S_R} \times \frac{\eta^2}{\eta_R^2}$$

Φ: quantum yield; *R*: reference fluorophore; *S*: slope of the fitted line; *η*: refractive index of the solvent.

7. Live-cell RNA imaging using color-shifting BeCA-BC6

7.1 Cloning of *pET28-BeCA*

The sequence encoding *BeCA* embedded in a *tRNA$_{Lys}$* scaffold was PCR-amplified by Q5 high-fidelity DNA polymerase (New England Biolabs GmbH) using plasmid *pET28-tRNA$_{Lys}$* as a template[4]. The PCR product was phosphorylated (T4 polynucleotide kinase) and ligated (T4 DNA ligase) to yield *pET28-BeCA*.

pET28-BeCA sequence	…TAATACGACTCACTATAGCGGATTTAGCTCAGTTGGGAGAGCGCCAGGGA GGTACGTGGACTGCTTATGCAGTAGGAAGGCCTCCCTGGAGGTCCTGTGTT CATCCACAGAATTCGCACCA…<br>(Blue: T7 promoter; Green: tRNA scaffold; Red: **BeCA** sequence)
Forward primer	ATGCAGTAGGAAGGCCTCCCTGGAGGTCCTGTGTTCG
Reverse primer	AAGCAGTCCACGTACCTCCCTGGCGCTCTCCCAACTG

7.2 Cloning of *pET28-gfp-BeCA$_8$*

A 182-nt single-stranded DNA ultramer was ordered from IDT. It contained 4 synonymous repeats of **BeCA** flanking with SalI and XhoI recognition sites at 5´- and 3´- ends, respectively. The ultramer was PCR-amplified using a forward and a reverse primer. The resulting DNA was double-digested with SalI and XhoI and ligated (T4 DNA ligase) into the 1GFP plasmid which had already been digested by SalI and dephosphorylated (Fast AP thermosensitive alkaline phosphate). The obtained plasmid containing 4 repeats of **BeCA** was further digested by XhoI, dephosphorylated and ligated again with the double-digested PCR product to yield *pET28-gfp-BeCA$_8$*.

DNA ultramer sequence	AAAGTCGACGGAGGTACGTGGACTGCTTATGCAGTAGGAAGGCCTCCAAAAGGTCCTACGTGGACTGCTTATGCAGTAGGAAGGGGACCAAAAGCTGCTACGTGGACTGCTTATGCAGTAGGAAGGGCAGCAAAACGAGCTACGTGGACTGCTTATGCAGTAGGAAGGGCTCGCTCGAGAAA (Blue: SalI; Green: XhoI; Red: **BeCA** sequence)
pET28-gfp-BeCA₈ sequence	…GGAGGTACGTGGACTGCTTATGCAGTAGGAAGGCCTCCAAAAGGTCCTACGTGGACTGCTTATGCAGTAGGAAGGGGACCAAAAGCTGCTACGTGGACTGCTTATGCAGTAGGAAGGGCAGCAAAACGAGCTACGTGGACTGCTTATGCAGTAGGAAGGGCTCGCTCGACAAGCTTGCGGCCGCACTCGACGGAGGTACGTGGACTGCTTATGCAGTAGGAAGGCCTCCAAAAGGTCCTACGTGGACTGCTTATGCAGTAGGAAGGGGACCAAAAGCTGCTACGTGGACTGCTTATGCAGTAGGAAGGGCAGCAAAACGAGCTACGTGGACTGCTTATGCAGTAGGAAGGGCTCG… (Red: **BeCA** sequence)
Forward primer	AAAGTCGACGGAGGTAC
Reverse primer	TTTCTCGAGCGAGCC

7.3 Cloning of *pAV-Tornado-BeCA$_4$*

The ultramer ssDNA containing four repeats of synonymous BeCA (section 7.2) was PCR amplified using a forward and a reverse primer. The resulting DNA was double-digested with NotI and SacII and ligated (T4 DNA ligase) *into pAV-U6+27-Tornado-Broccoli* (Addgene, plasmid no. 124360) which had already been digested by NotI and SacII to yield *pAV-Tornado-BeCA$_4$.*

pAV-Tornado-BeCA$_4$ sequence	…AGTGCGGCCGCTGGAGGTACGTGGACTGCTTATGCAGTAGGAAGGTCTCCAAAAGGTCCTACGTGGACTGCTTATGCAGTAGGAAGGGGACCAAAAGCTGCTACGTGGACTGCTTATGCAGTAGGAAGGGCAGCAAAACGAGCTACGTGGACTGCTTATGCAGTAGGAAGGGCTCGAGTGGCCGCGGTCG… (Blue: NotI; Green: SacII; Red: **BeCA** sequence)
Forward primer	CCGAGTGCGGCCGCTGGAGGTACGTGGACT
Reverse primer	CGCCGACCGCGGCCACTCGAGCCCTTCCTACT

7.4 General method for live-cell imaging in bacteria

BL21 Star™ (DE3) competent *E. coli* cells were transformed with the plasmid of interest and grown overnight on LB agar plates (30 µg/mL kanamycin) at 37 °C. A single colony was picked and grown in LB medium (30 µg/mL kanamycin) at 37 °C overnight with shaking (170 rpm). The next day, a fresh culture in LB medium (30 µg/mL kanamycin) was started with an OD_{600} of 0.02. When OD_{600} reached 0.4, IPTG was added to the medium to initiate transcription (170 rpm, 37 °C, 3 h). 300 µL of the bacterial culture was removed, spun down (6000 rpm, 2 min), washed, and

resuspended in M9 buffer (1 mL). The suspension was transferred into a poly-D-lysine coated 8-well glass chamber. After cell attachment (room temperature, 15 min), the cells were washed twice with M9 buffer and incubated with **BC6** (in M9 buffer containing 0.2% DMSO) for 15 min at 37 °C. Images were taken on a point scanning confocal microscope with hybrid-scanner (galvano/resonant). Objective: Nikon N Apo 60 × NA 1.4 λs OI (WD 0.14 mm, FOV 0.21 × 0.21 mm) objective; Lasers: 405 nm (cyan channel), 488 nm (green channel), 640 nm (NIR channel); Signal detection (center wavelength [nm]/bandwidth [nm]): 450/50 (cyan channel), 515/30 (green channel), 700/75 (NIR channel). Images were analyzed by ImageJ Fiji.

7.5 Total RNA isolation and gel analysis of BeCA

The bacterial cultures treated with different IPTG amounts (0, 10, 20, 50, 100, 1000 µM) were prepared as described above (section 7.4). After 3 h of induction, 3 mL of bacterial culture from each IPTG-treated group was collected, washed and resuspended in M9 buffer (600 µL). The OD_{600} value of each group was recorded and the bacterial suspensions were normalized to the same number of bacteria according to the OD_{600} values (final volume: 450 µL in M9 buffer). A small portion (50 µL) of the cells was used for imaging while the rest was used for total RNA isolation. Total RNA from the bacteria was isolated using RNAzol®RT according to the manufacturers' instructions. Precipitated RNA was dissolved in water (100 µL) and 10 µL was loaded on a 10% PAGE gel. The gel was stained using ethidium bromide.

7.6 General method for circular RNA imaging in mammalian cells

HEK293T cells were cultured at 37 °C under 5% CO_2 in Dulbecco's Modified Eagle's Medium (high glucose, without phenol red) supplemented with 10% FBS, 100 U/mL of penicillin and 100 µg/mL of streptomycin. For imaging experiments, cells were seeded in a poly-D-lysine coated 8-well glass chamber containing 300 µl of medium each overnight. On the following day, cells were transfected with the appropriate plasmid using the FuGeneHD transfection reagent (Promega) according to the manufacturer's protocol. After 48 h, the medium was exchanged with Leibowitz (L15) medium containing 500 nM of **BC6**. After incubation for 1 h, cells were imaged at 37 °C. Images were taken as described for bacterial cells and analyzed by ImageJ Fiji.

8. Supplemental Spectra

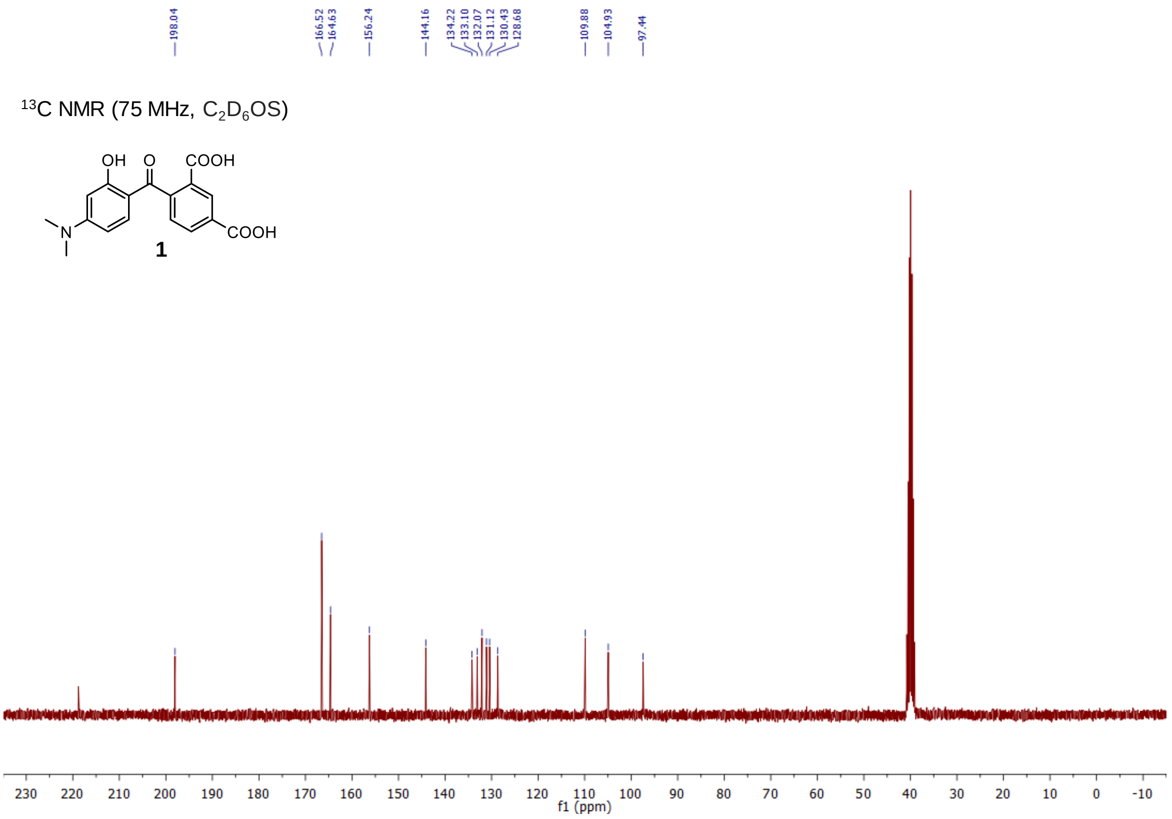

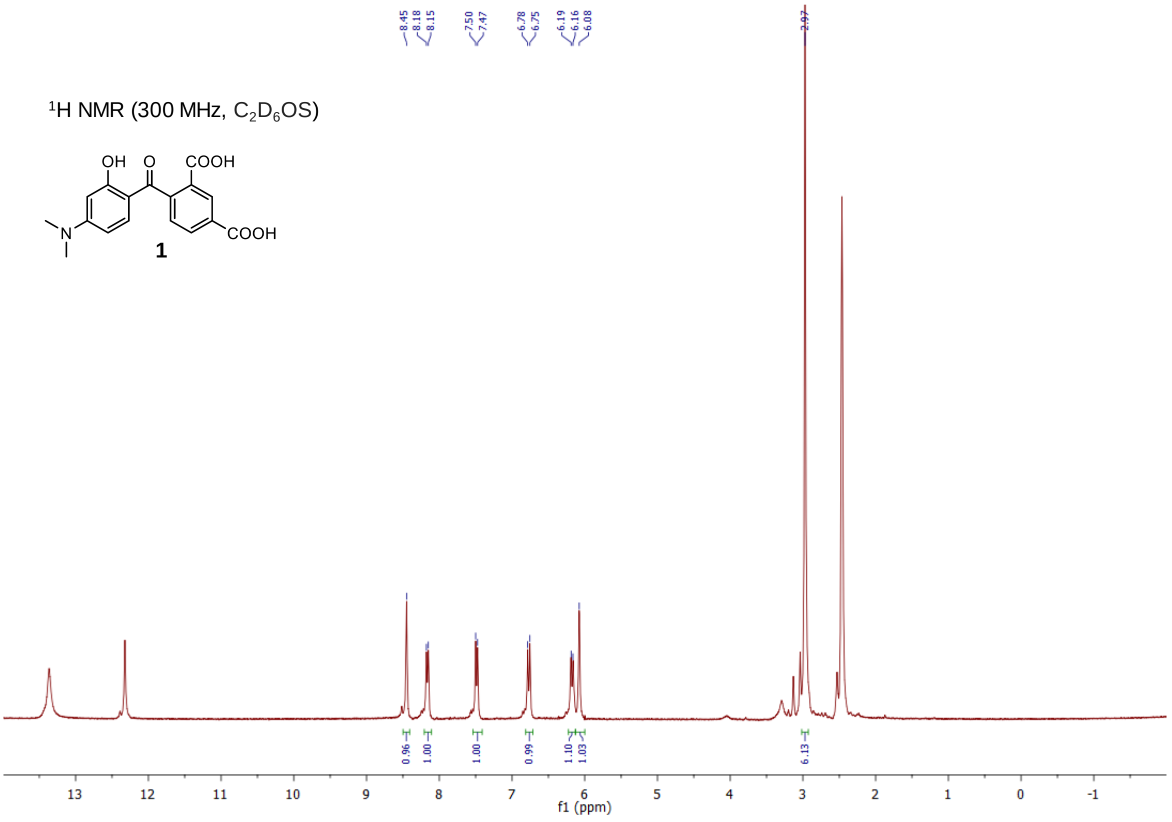

^{1}H NMR (300 MHz, $CDCl_3$)

2

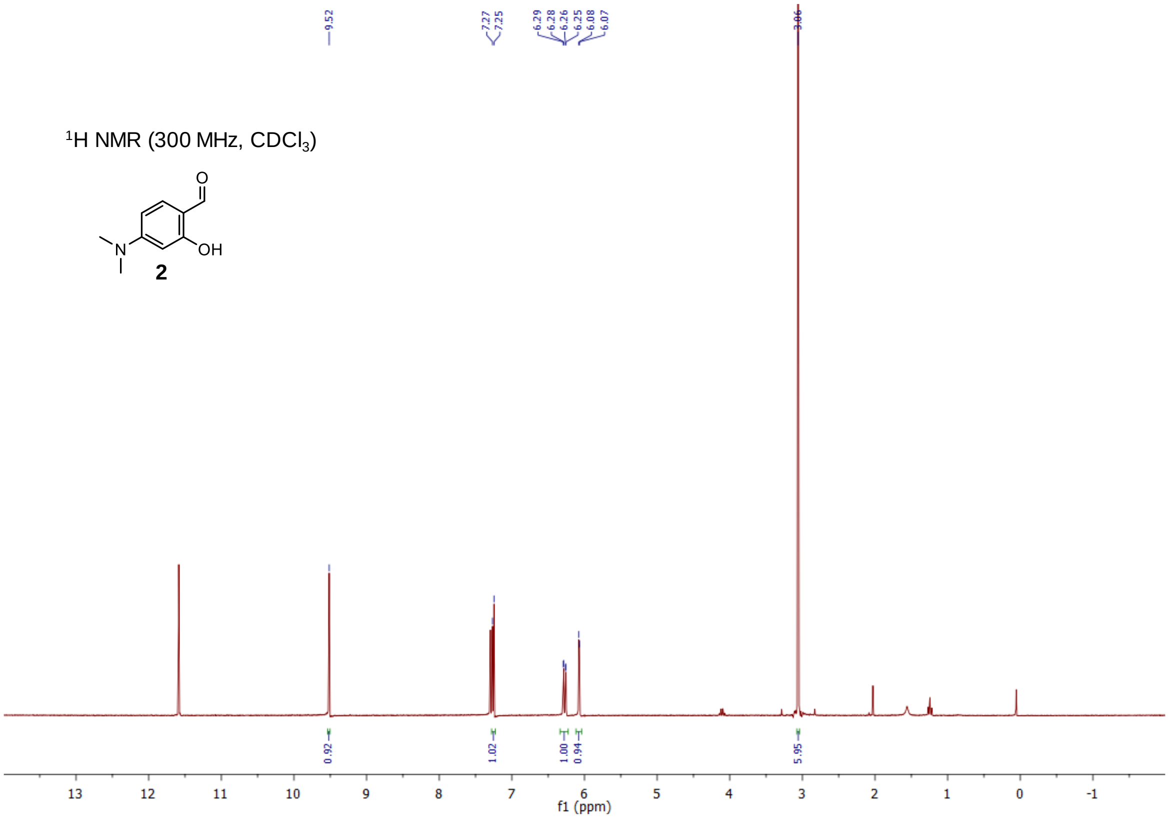

^{13}C NMR (75 MHz, $CDCl_3$)

2

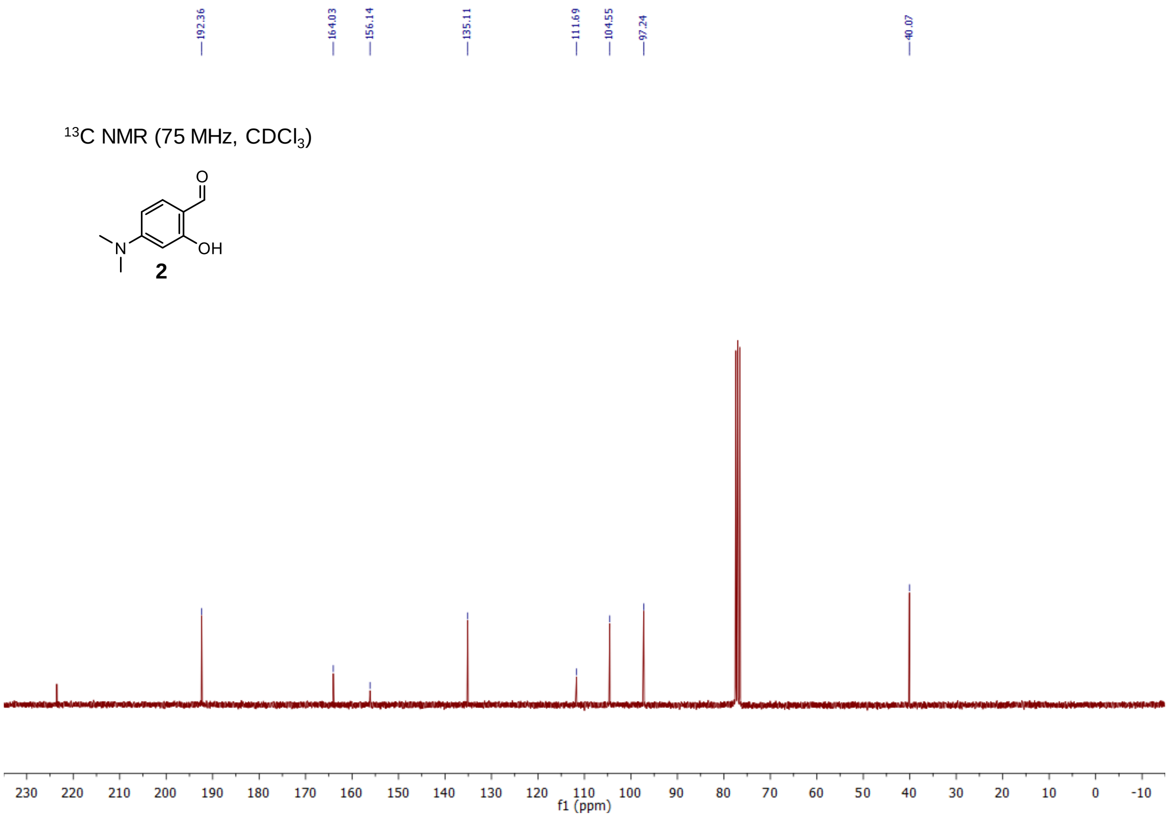

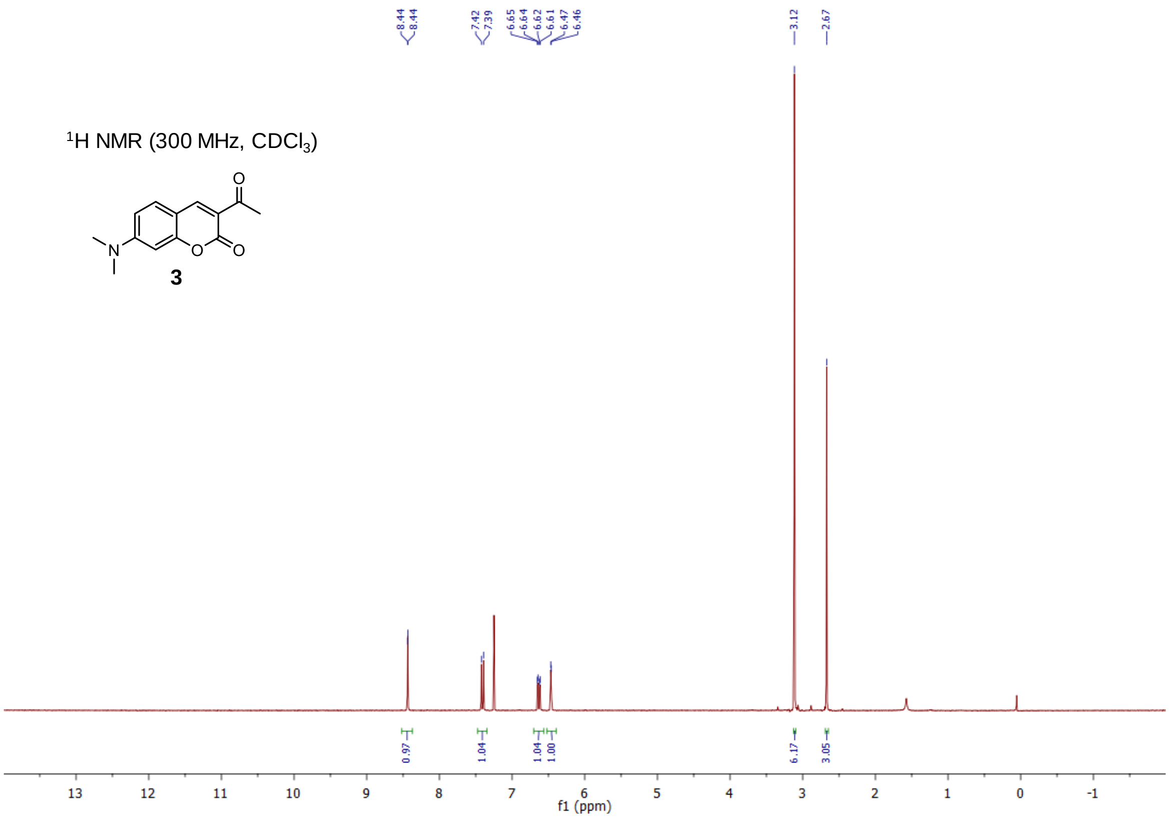

^{1}H NMR (300 MHz, $CDCl_3$)
3
8.44
8.44
7.42
7.39
6.65
6.64
6.62
6.61
6.47
6.46
3.12
2.67
0.97
1.04
1.04
1.00
6.17
3.05
13 12 11 10 9 8 7 6 5 4 3 2 1 0 -1
f1 (ppm)

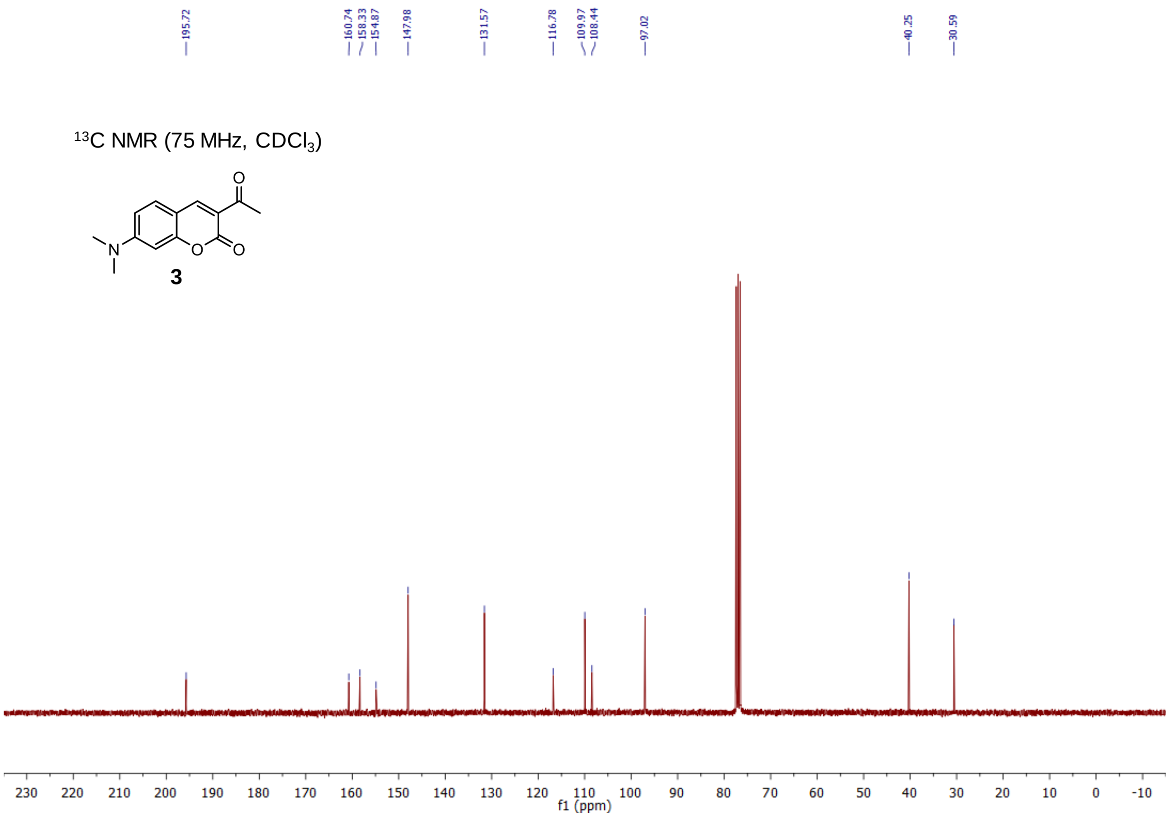

^{13}C NMR (75 MHz, $CDCl_3$)
3
195.72
160.74
158.33
154.87
147.98
131.57
116.78
109.97
108.44
97.02
40.25
30.59
230 220 210 200 190 180 170 160 150 140 130 120 110 100 90 80 70 60 50 40 30 20 10 0 -10
f1 (ppm)

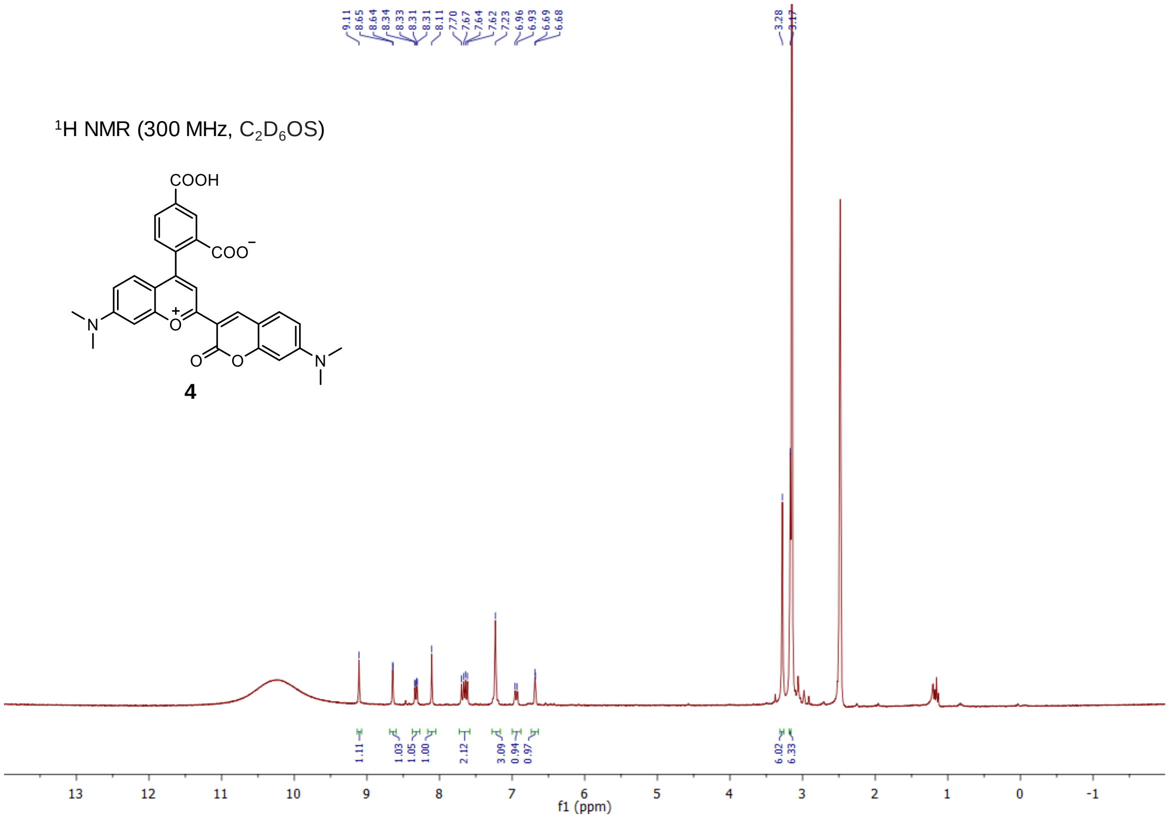
^{1}H NMR (300 MHz, C_2D_6OS)
COOH
COO$^-$
N
O
+
O
O
N
4
9.11 8.65 8.64 8.34 8.33 8.31 8.31 8.11 7.70 7.67 7.64 7.62 7.23 6.96 6.93 6.69 6.68
3.28 3.17
1.11 1.03 1.05 1.00 2.12 3.09 0.94 0.97
6.02 6.33
13 12 11 10 9 8 7 6 5 4 3 2 1 0 -1
f1 (ppm)

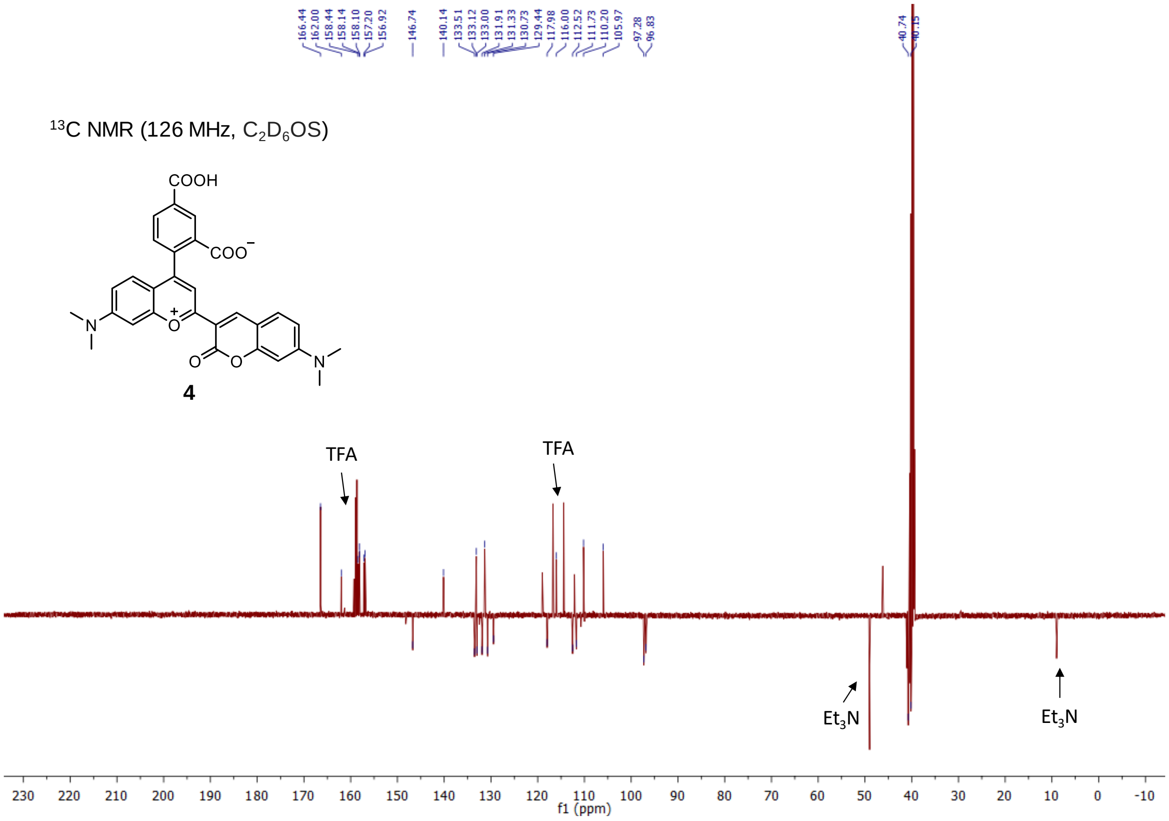
^{13}C NMR (126 MHz, C_2D_6OS)
COOH
COO$^-$
N
O
+
O
O
N
4
166.44 162.00 158.44 158.14 158.10 157.20 156.92 146.74 140.14 133.51 133.12 133.00 131.91 131.33 130.73 129.44 117.98 116.00 112.52 111.73 110.20 105.97 97.28 96.83
40.74 40.15
TFA
TFA
Et_3N
Et_3N
230 220 210 200 190 180 170 160 150 140 130 120 110 100 90 80 70 60 50 40 30 20 10 0 -10
f1 (ppm)

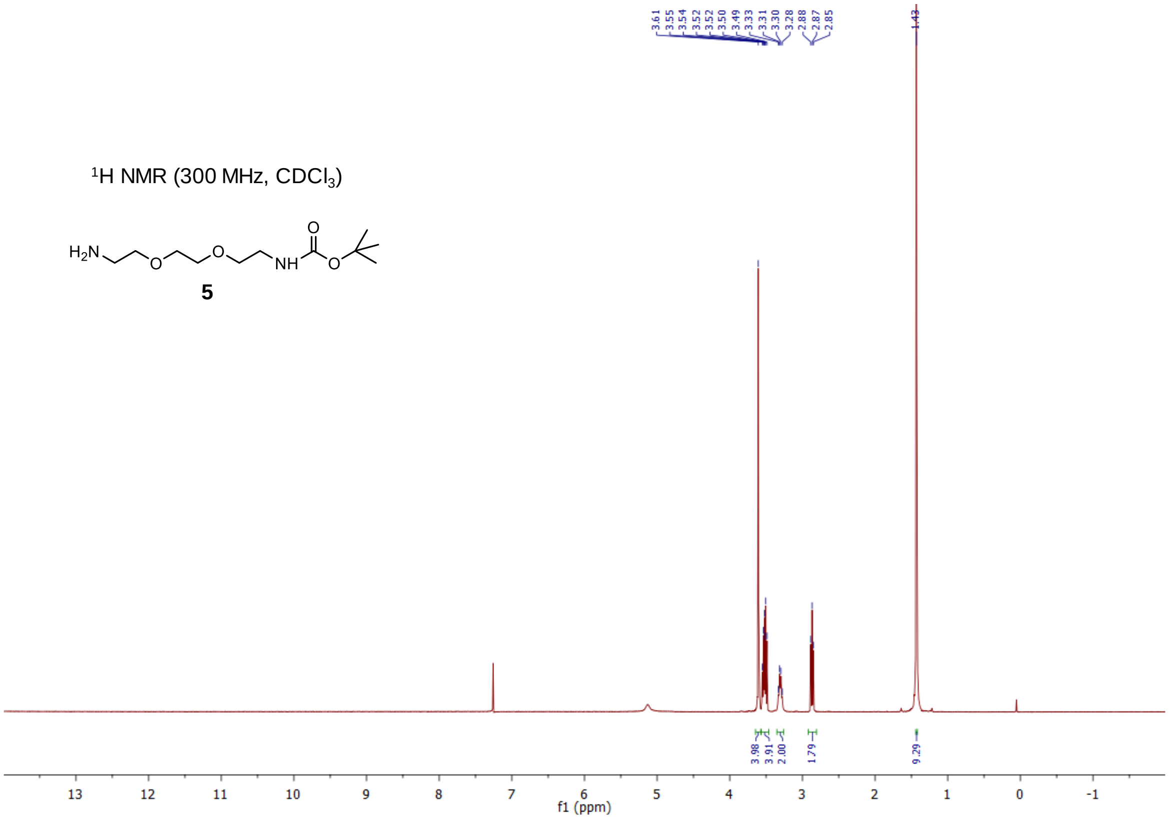

^{1}H NMR (300 MHz, $CDCl_3$)
5
f1 (ppm)

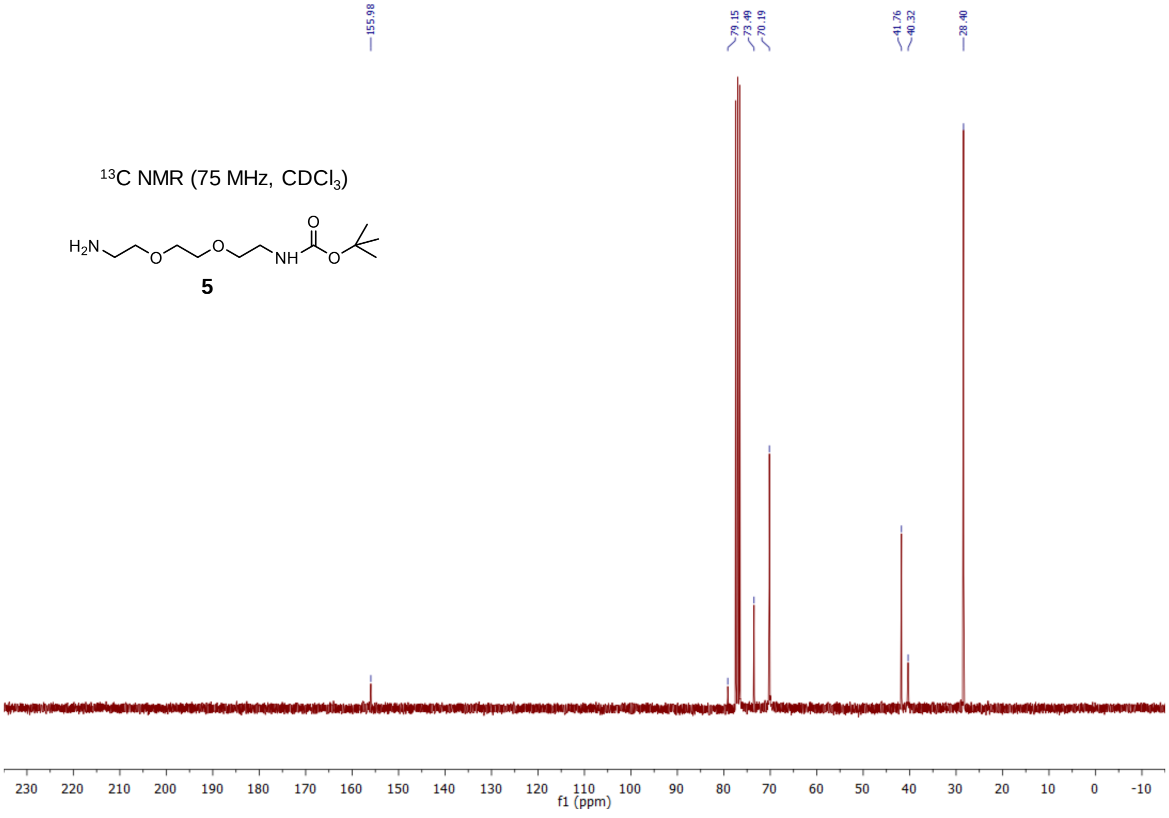

^{13}C NMR (75 MHz, $CDCl_3$)
5
f1 (ppm)

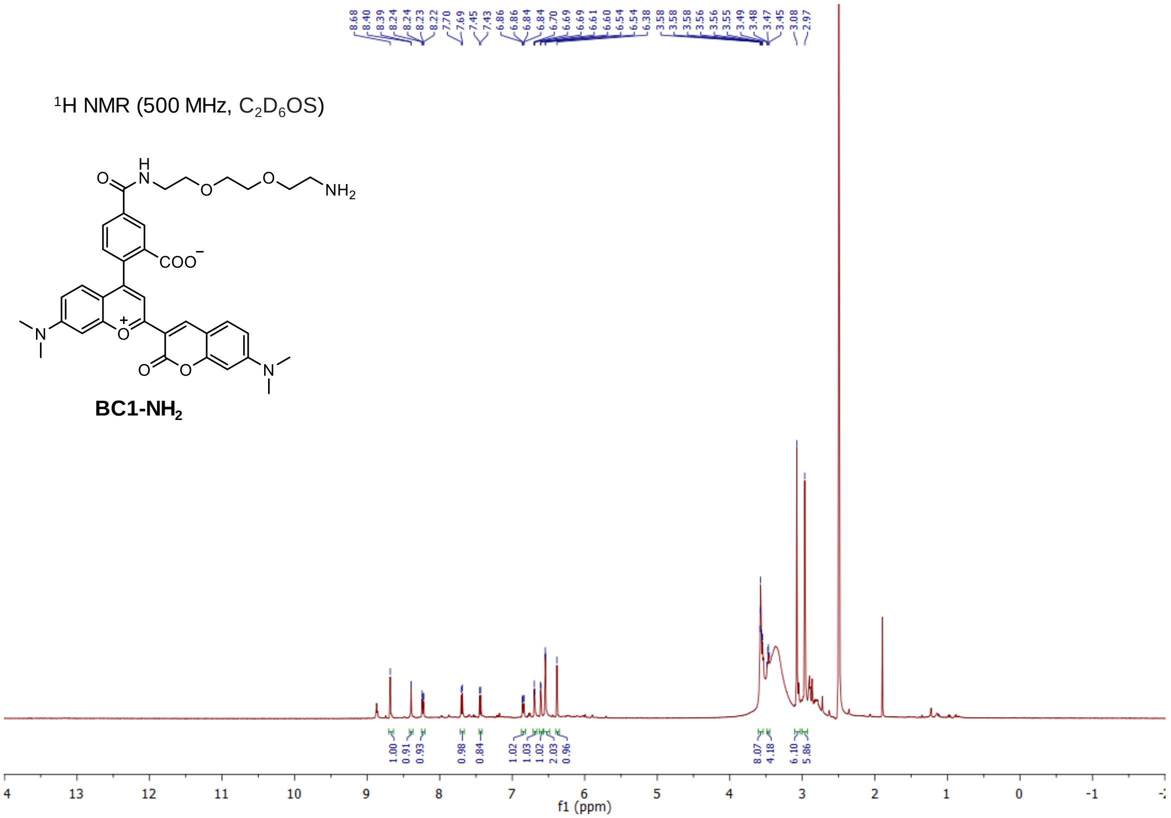
1H NMR (500 MHz, C2D6OS)
BC1-NH2
8.68 8.40 8.39 8.24 8.24 8.23 8.22 7.70 7.69 7.45 7.43 6.86 6.86 6.84 6.84 6.70 6.69 6.69 6.61 6.60 6.54 6.38 3.58 3.58 3.56 3.56 3.55 3.49 3.48 3.47 3.45 3.08 2.97
1.00 0.91 0.93 0.98 0.84 1.02 1.03 1.02 2.03 0.96 8.07 4.18 6.10 5.86
f1 (ppm)

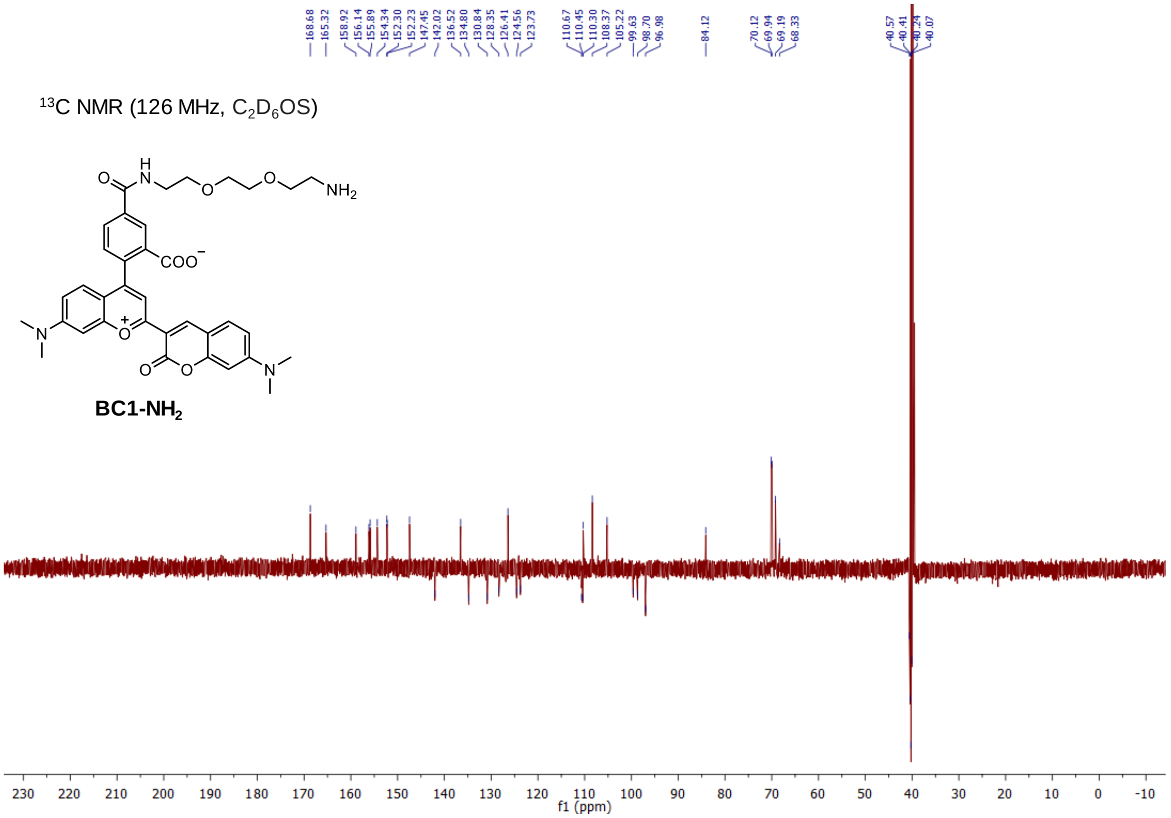
13C NMR (126 MHz, C2D6OS)
BC1-NH2
168.68 165.32 158.92 156.14 155.89 154.34 152.30 152.23 147.45 142.02 136.52 134.80 130.84 128.35 126.41 124.56 123.73 110.67 110.45 110.30 108.37 105.22 99.63 98.70 96.98 84.12 70.12 69.94 69.19 68.33 40.57 40.41 40.24 40.07
f1 (ppm)

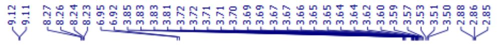

^{1}H NMR (300 MHz, $CDCl_3$)

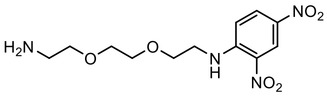

7

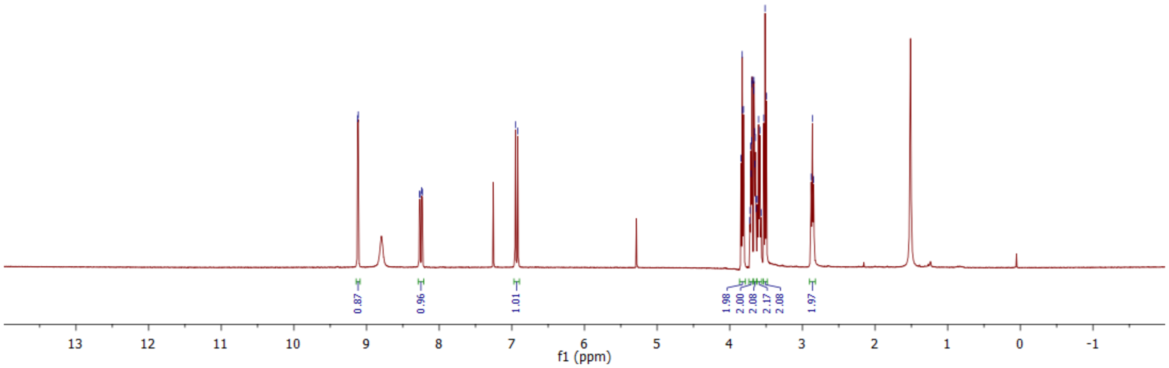

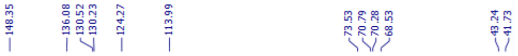

^{13}C NMR (75 MHz, $CDCl_3$)

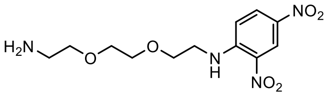

7

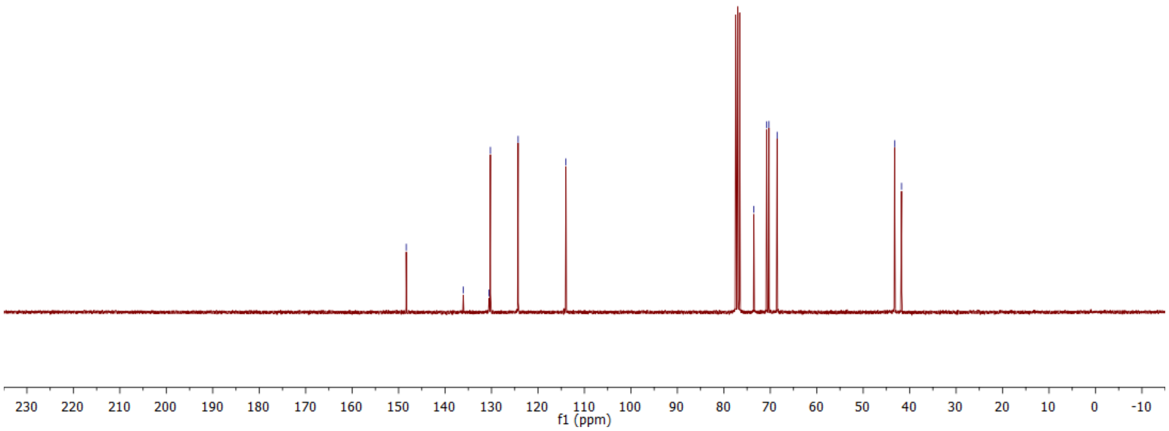

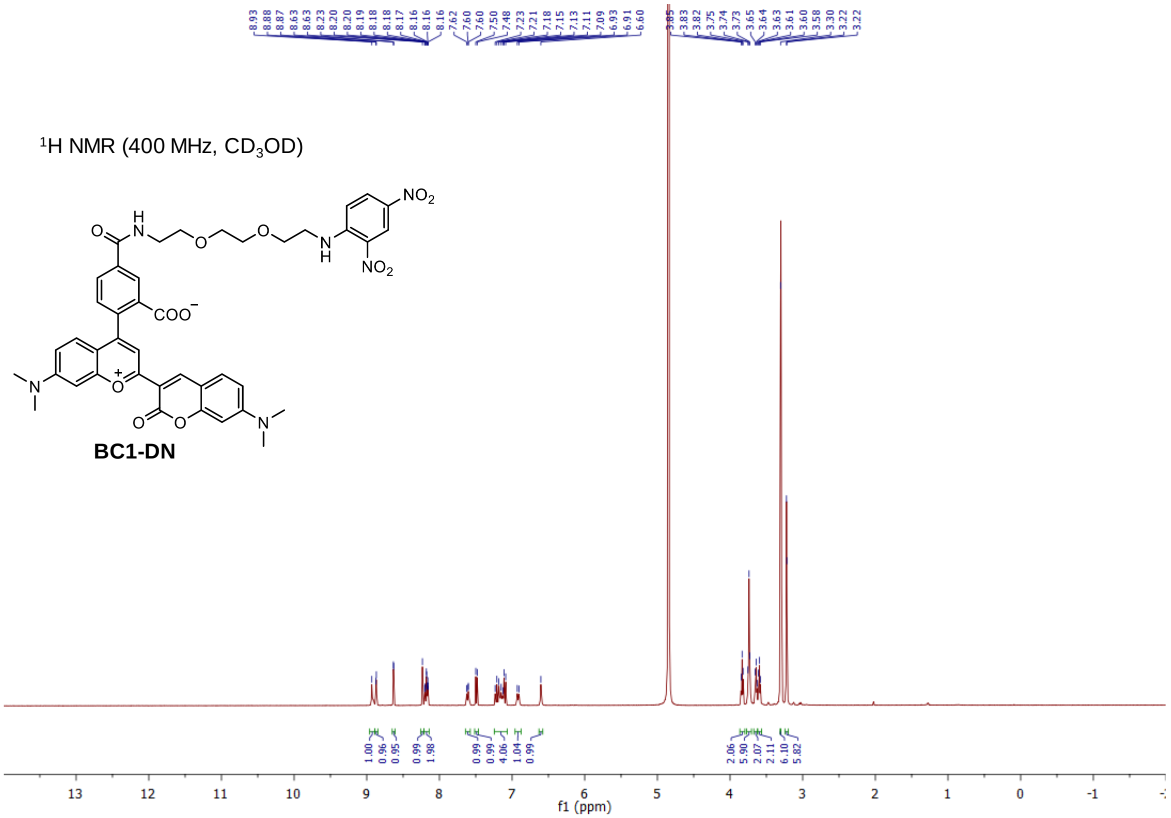

^{1}H NMR (400 MHz, CD_3OD)
BC1-DN
f1 (ppm)

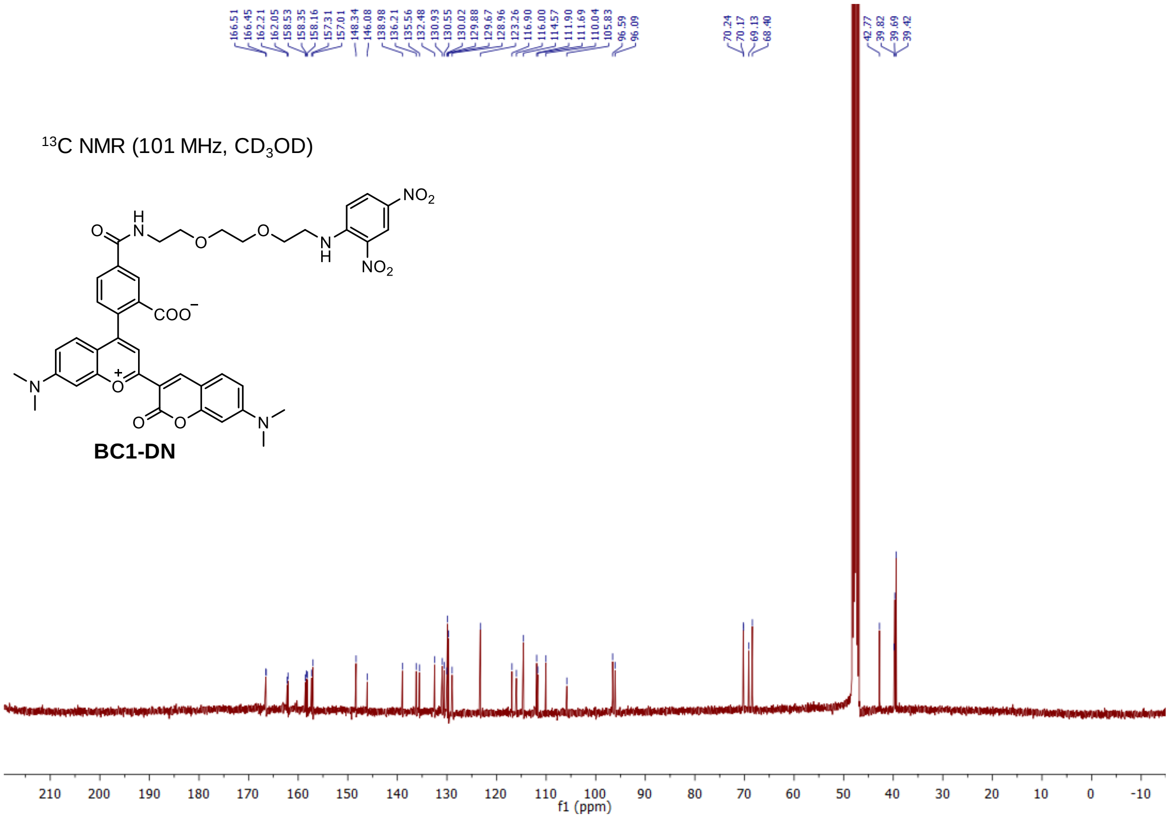

^{13}C NMR (101 MHz, CD_3OD)
BC1-DN
f1 (ppm)

^{1}H NMR (300 MHz, $CDCl_3$)

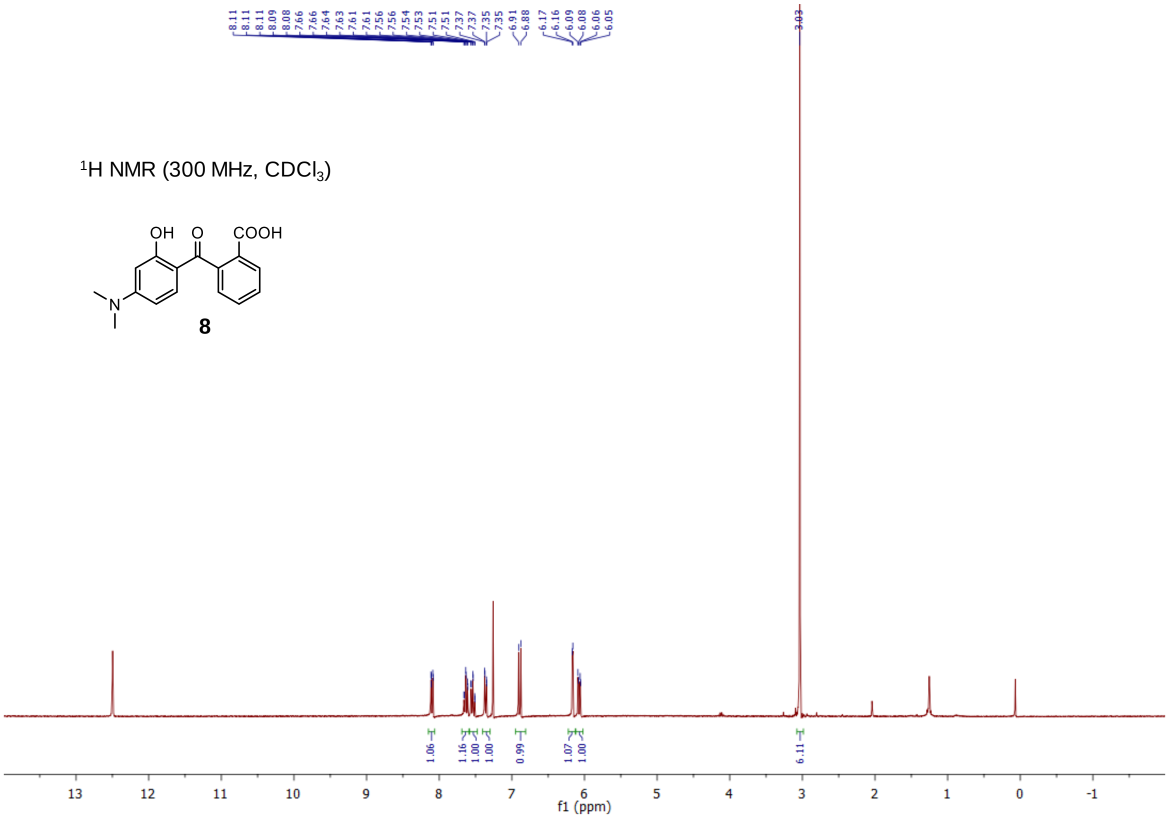

8

^{13}C NMR (75 MHz, $CDCl_3$)

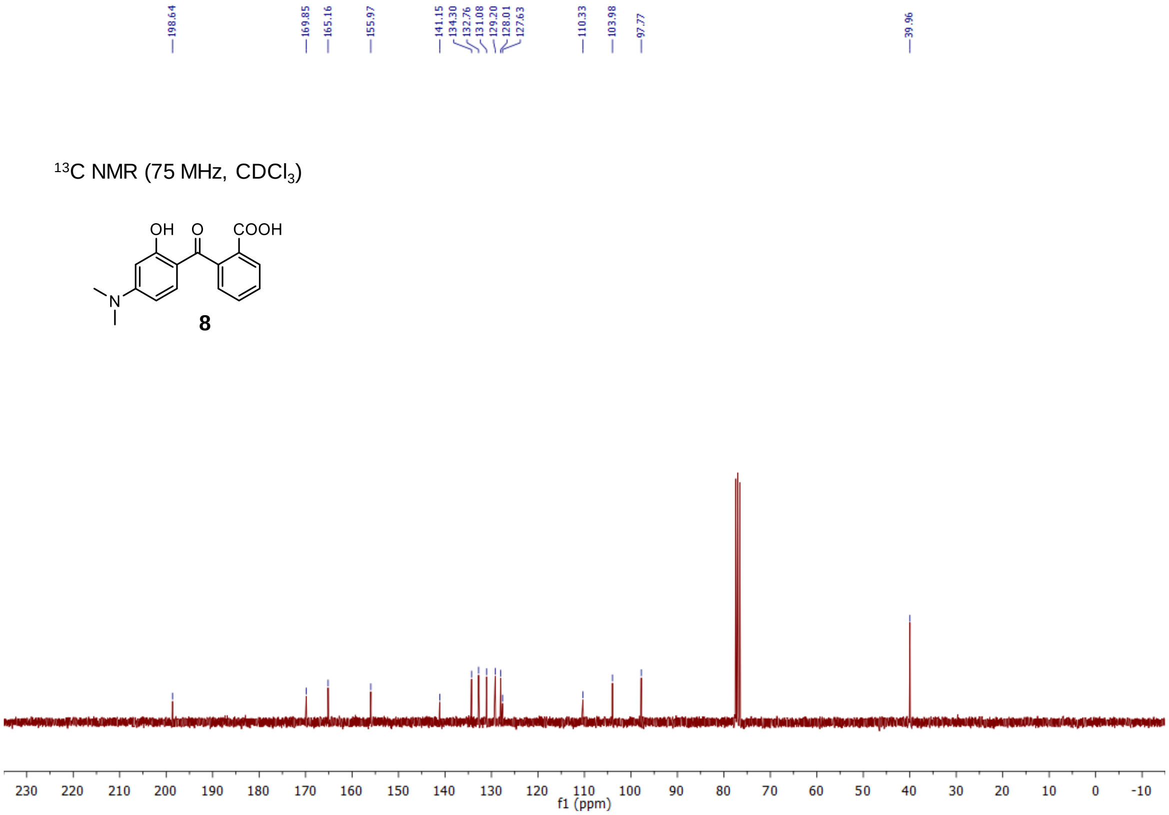

8

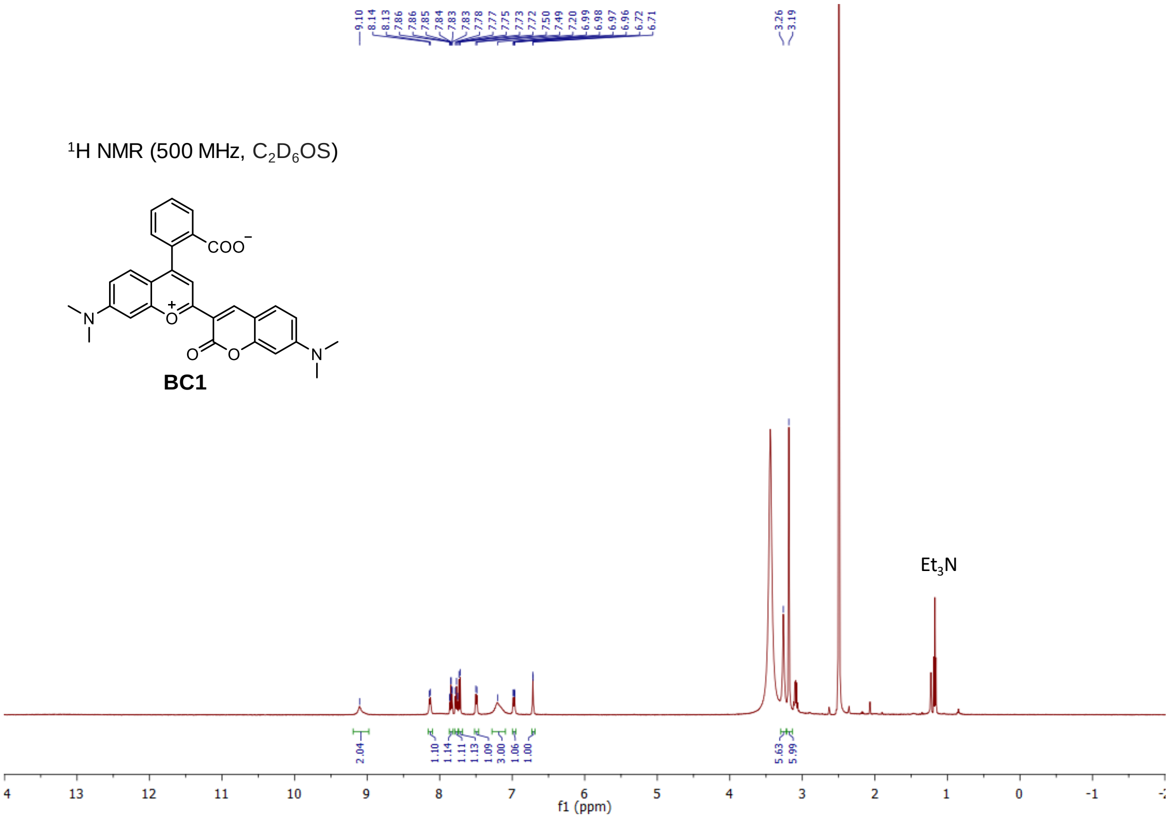

^{1}H NMR (500 MHz, C_2D_6OS)
BC1
Et$_3$N
f1 (ppm)

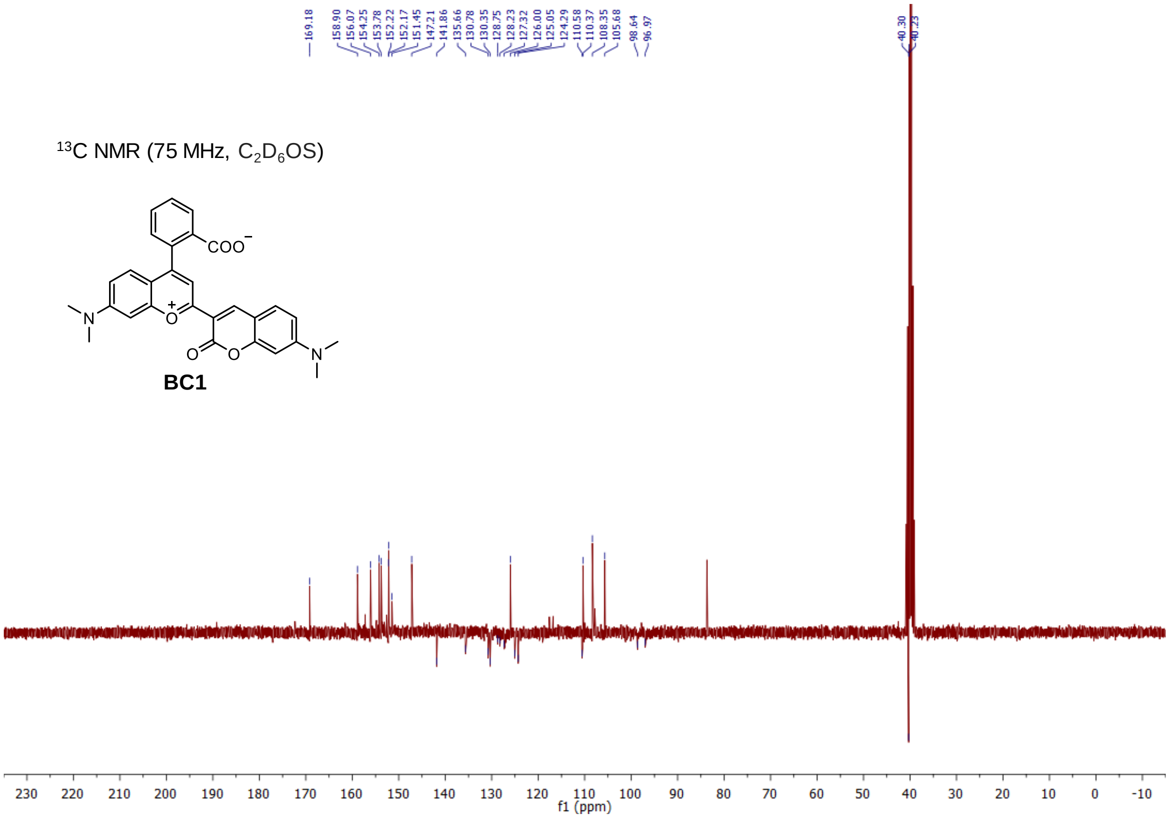

^{13}C NMR (75 MHz, C_2D_6OS)
BC1
f1 (ppm)

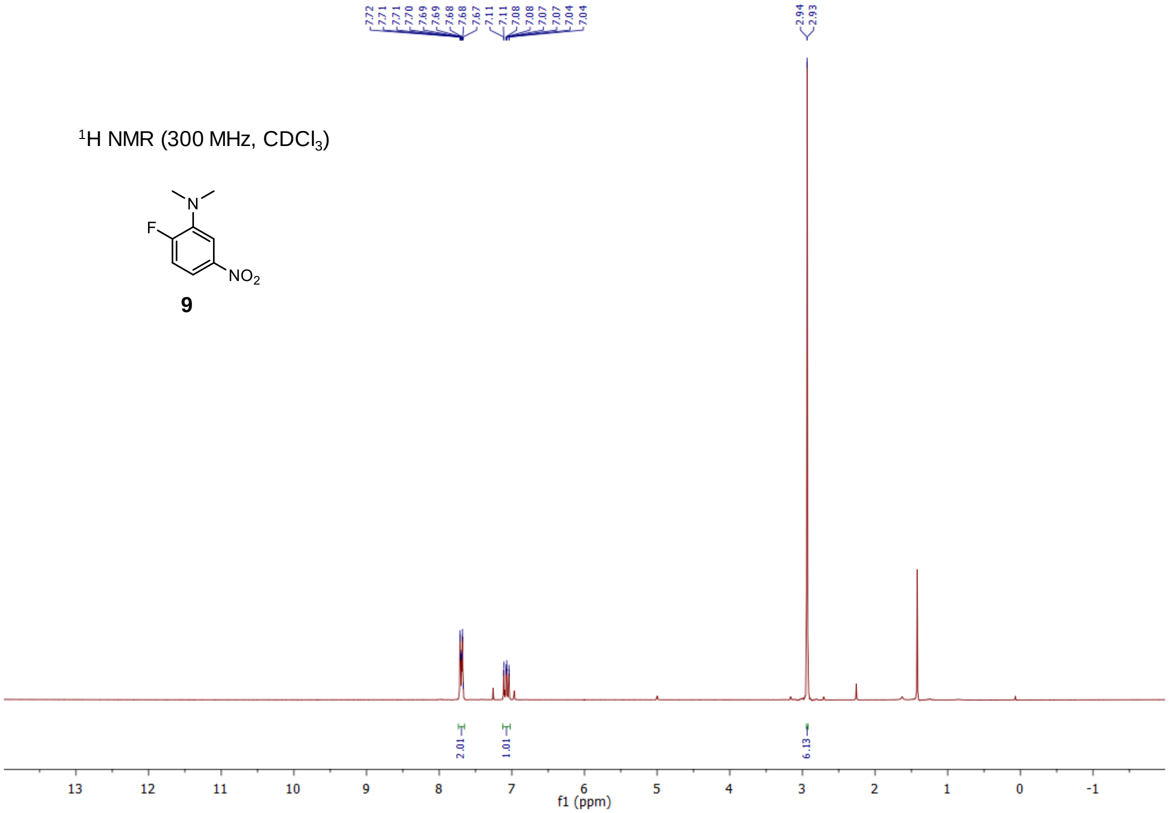

^{1}H NMR (300 MHz, $CDCl_3$)

9

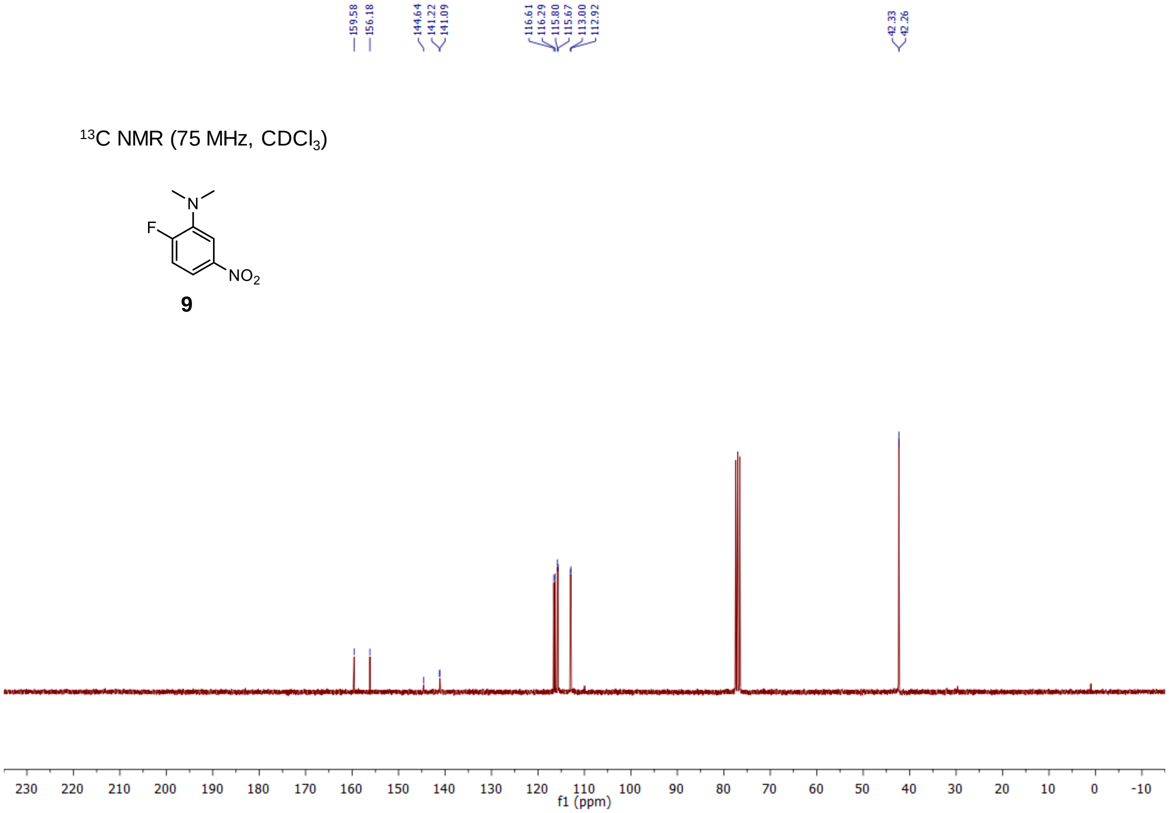

^{13}C NMR (75 MHz, $CDCl_3$)

9

^{1}H NMR (300 MHz, $CDCl_3$)

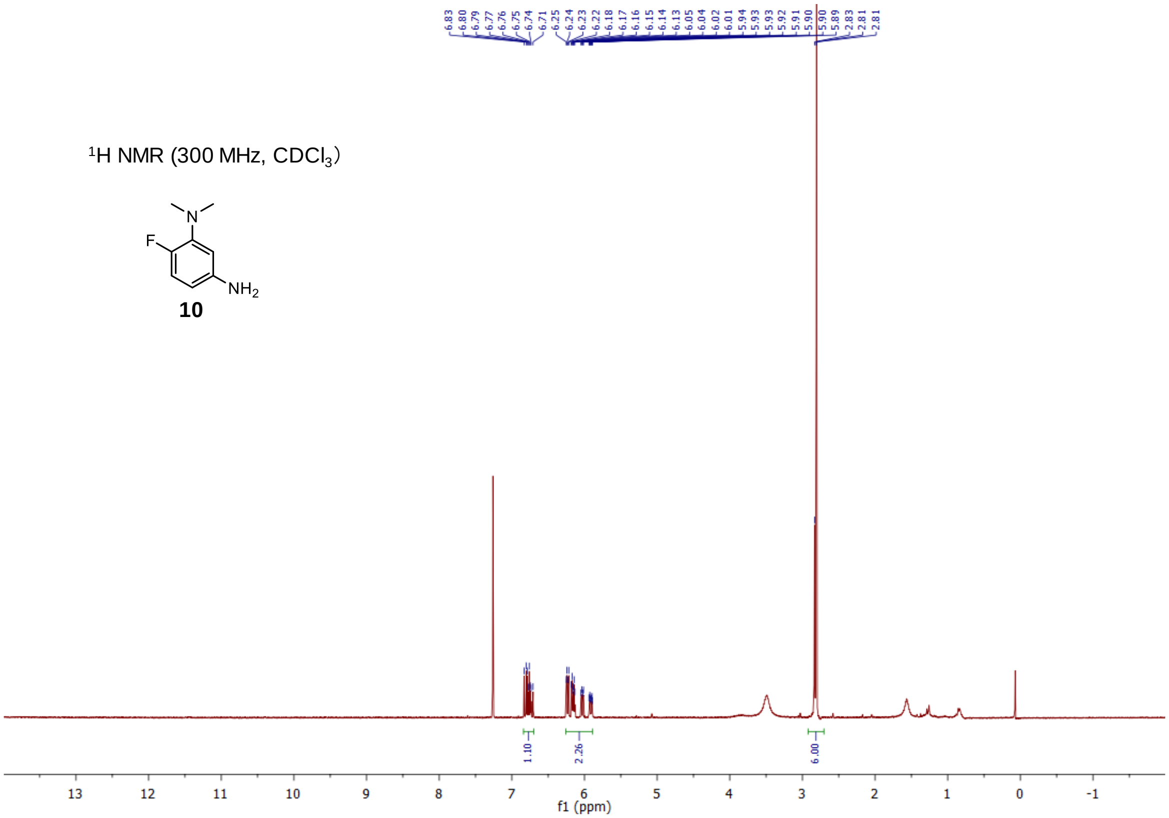

10

^{13}C NMR (75 MHz, $CDCl_3$)

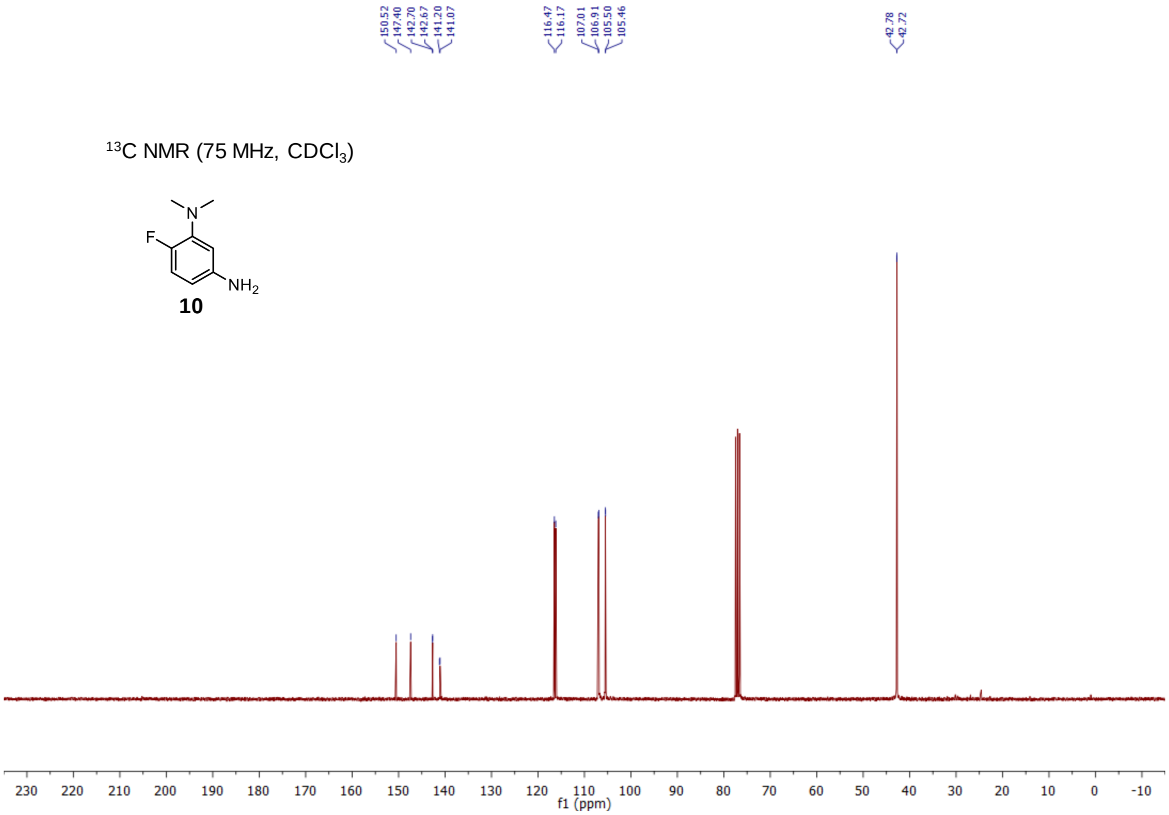

10

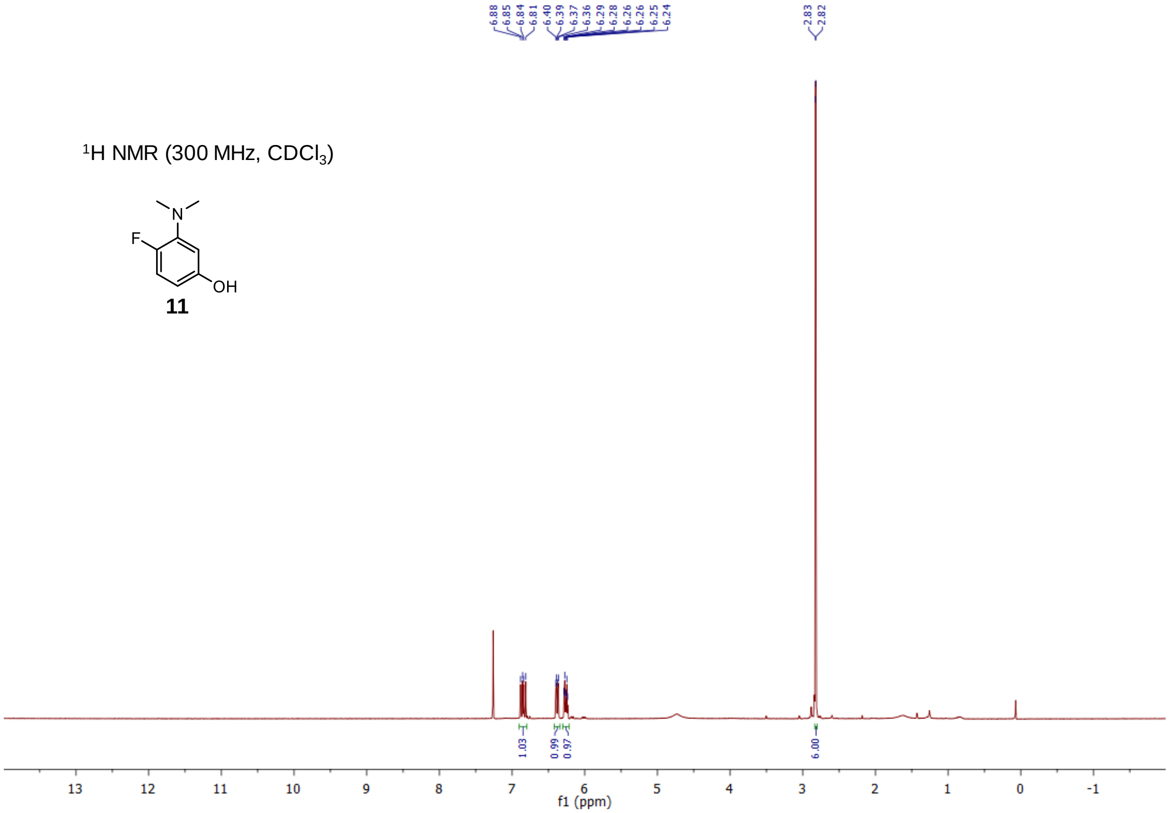

^{1}H NMR (300 MHz, $CDCl_3$)
F
N
OH
11
6.88 6.85 6.84 6.81 6.40 6.39 6.37 6.36 6.29 6.28 6.26 6.26 6.25 6.24
2.83 2.82
1.03
0.99
0.97
6.00
f1 (ppm)

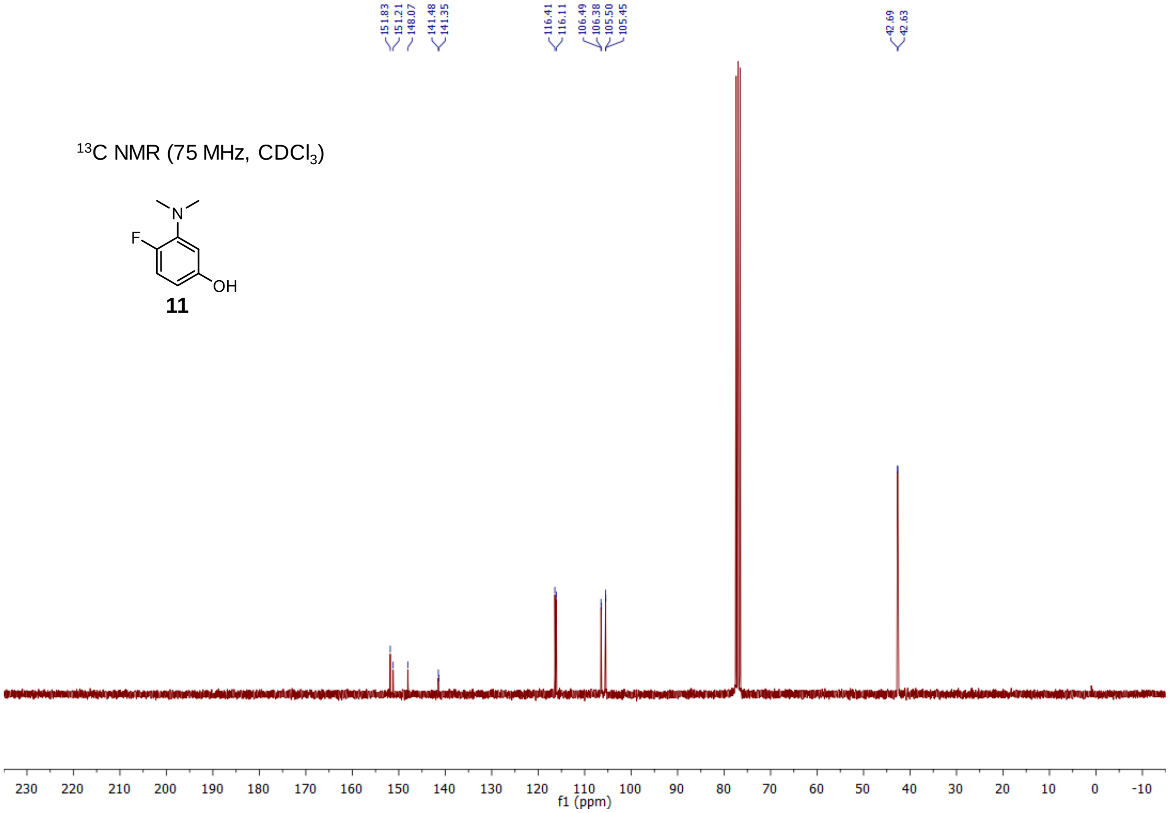

^{13}C NMR (75 MHz, $CDCl_3$)
F
N
OH
11
151.83 151.21 148.07 141.48 141.35
116.41 116.11 106.49 106.38 105.50 105.45
42.69 42.63
f1 (ppm)

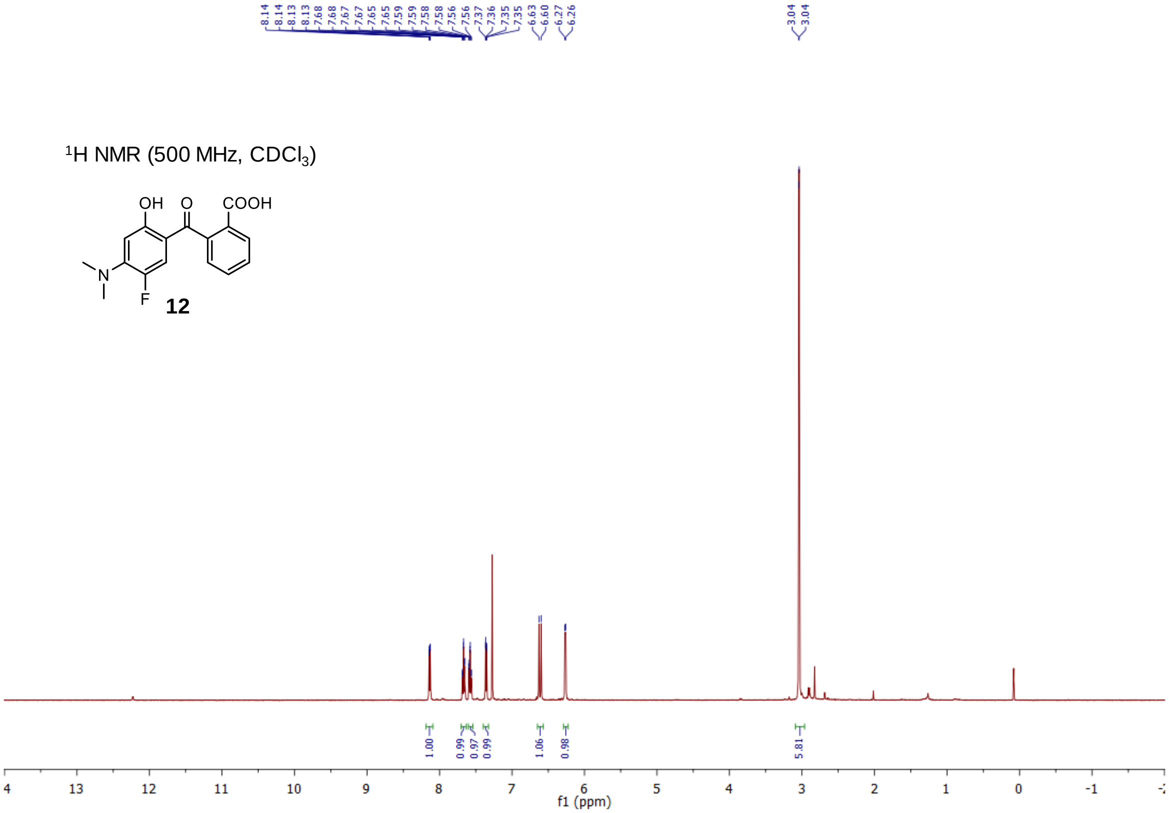
8.14
8.14
8.13
8.13
7.68
7.68
7.67
7.65
7.65
7.59
7.59
7.58
7.58
7.56
7.56
7.37
7.36
7.35
7.35
6.63
6.60
6.27
6.26
3.04
3.04
^{1}H NMR (500 MHz, $CDCl_3$)
OH
O
COOH
N
F
12
1.00
0.99
0.97
0.99
1.06
0.98
5.81
f1 (ppm)

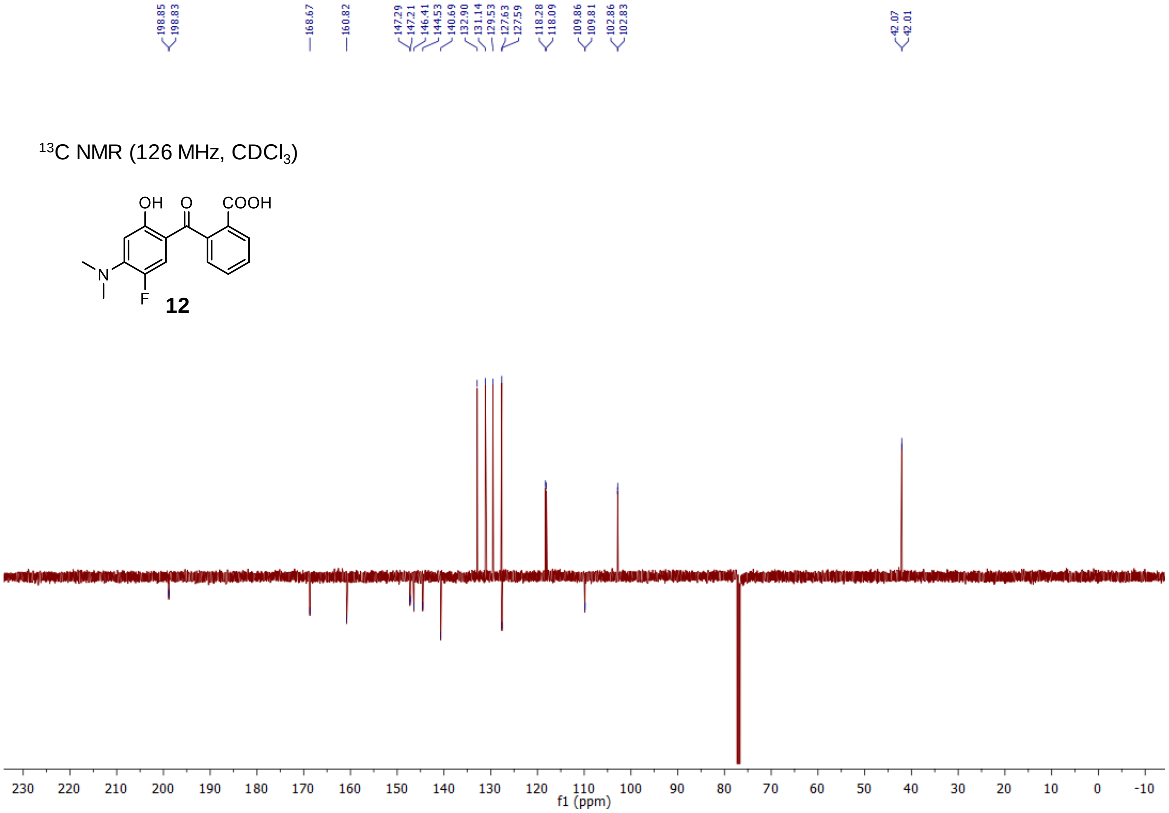
198.85
198.83
168.67
160.82
147.29
147.21
146.41
144.53
140.69
132.90
131.14
129.53
127.63
127.59
118.28
118.09
109.86
109.81
102.86
102.83
42.07
42.01
^{13}C NMR (126 MHz, $CDCl_3$)
OH
O
COOH
N
F
12
f1 (ppm)

^{1}H NMR (300 MHz, $CDCl_3$)

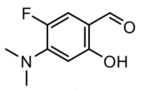

13

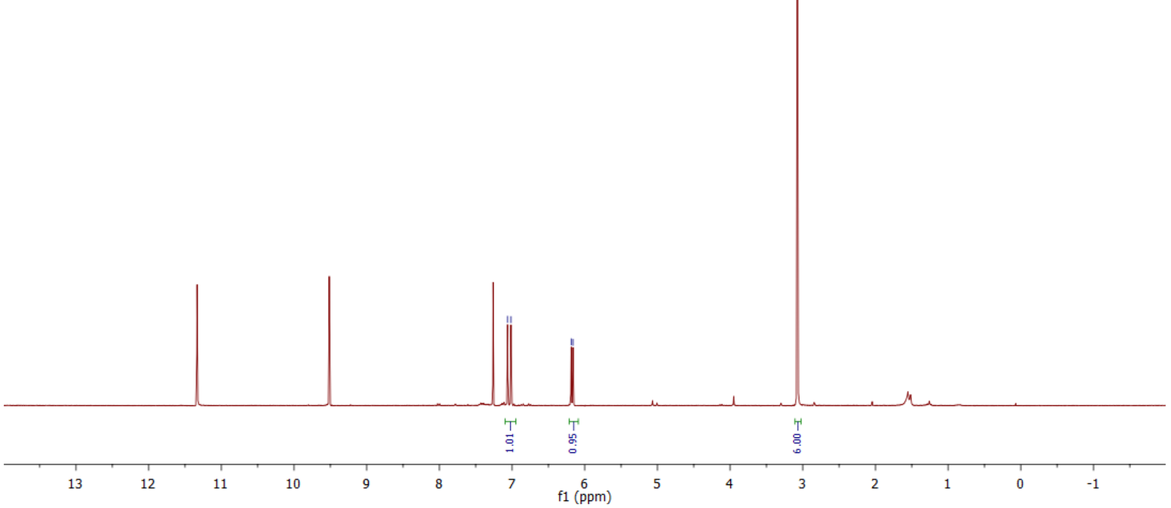

^{13}C NMR (75 MHz, $CDCl_3$)

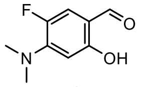

13

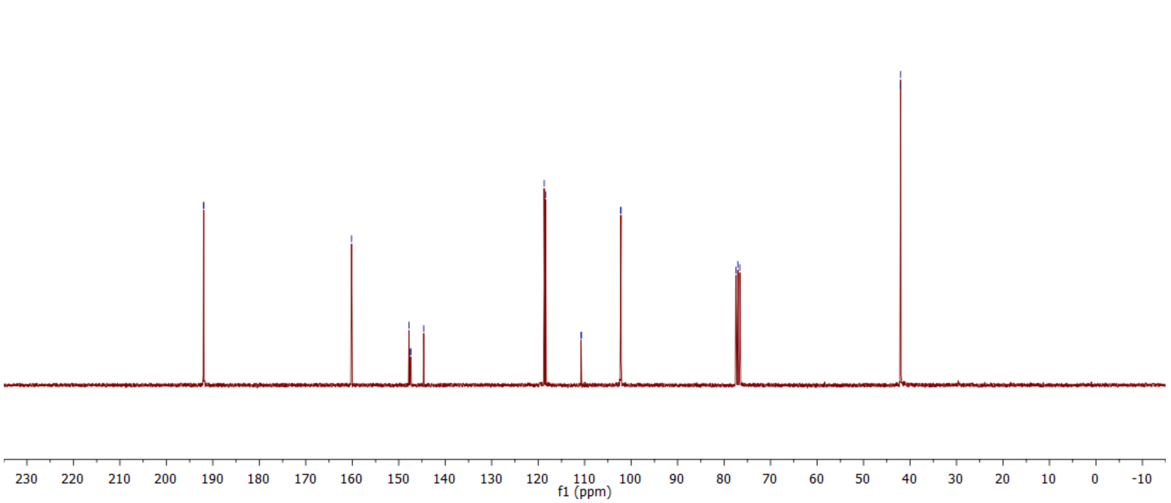

^{1}H NMR (500 MHz, $CDCl_3$)

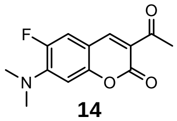

14

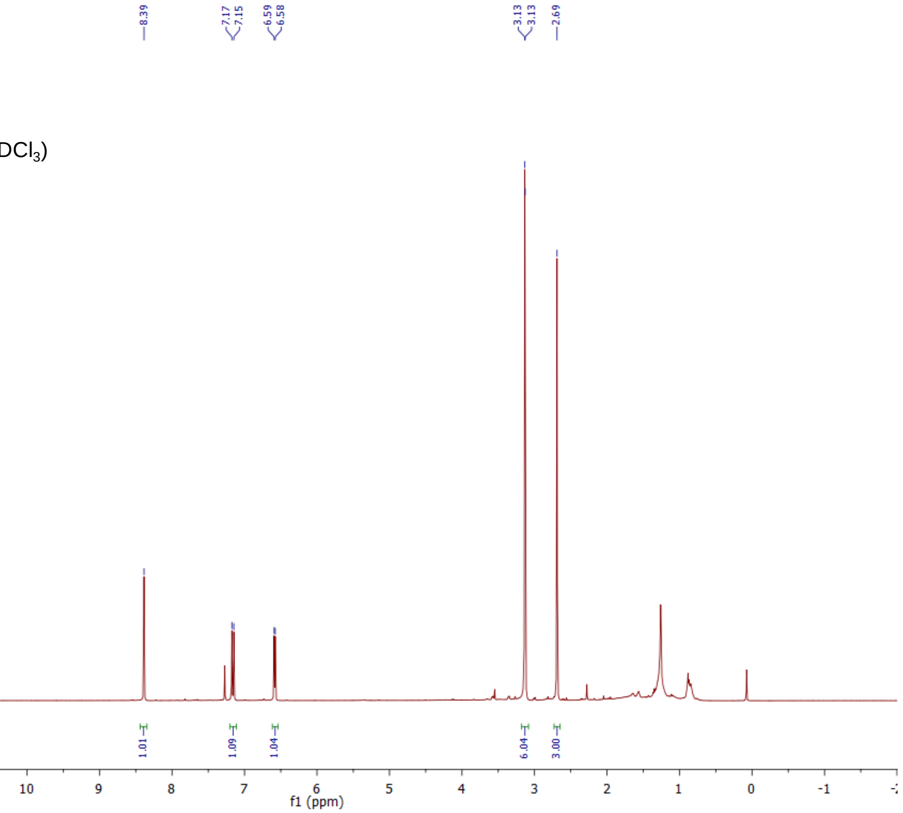

^{13}C NMR (126 MHz, $CDCl_3$)

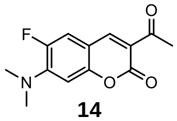

14

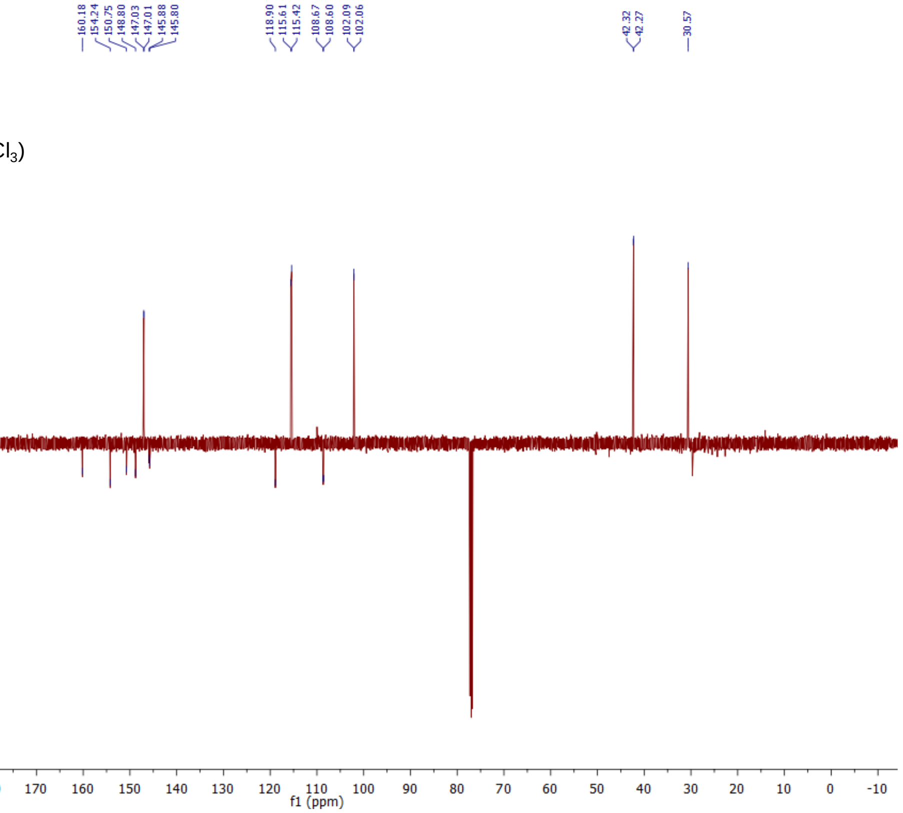

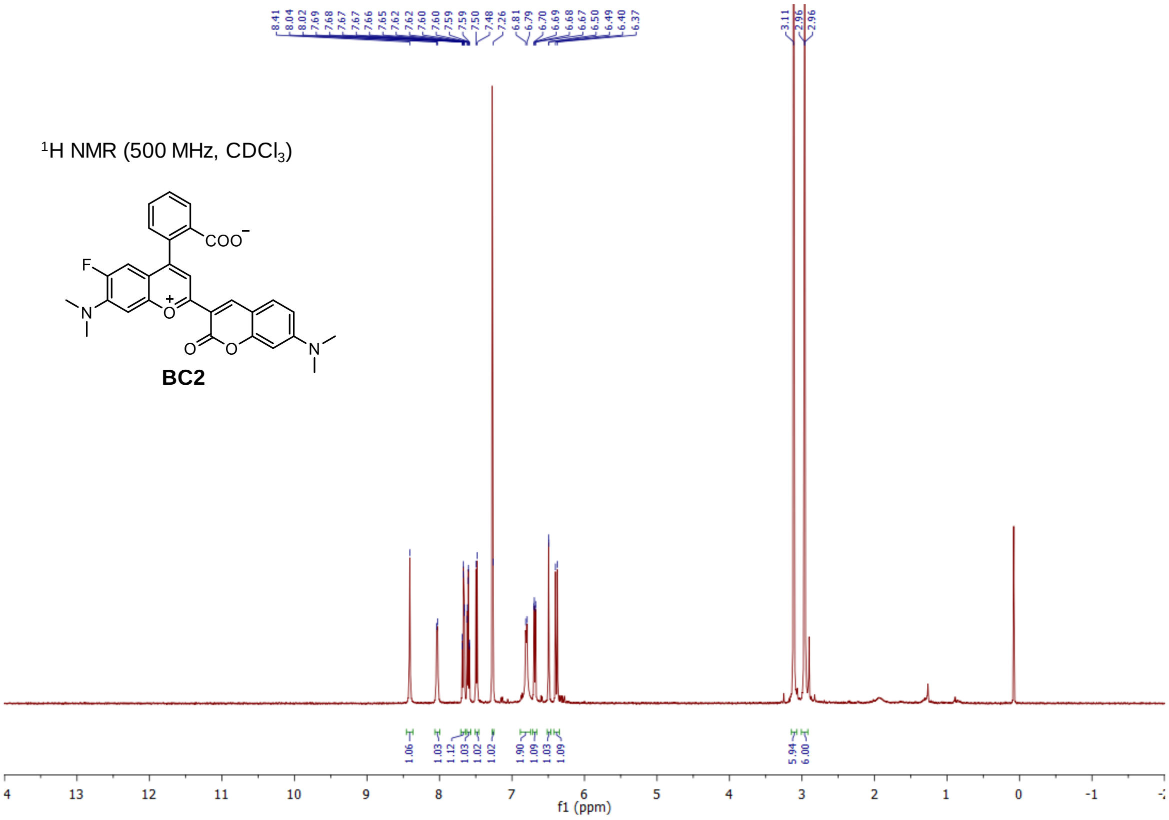

^{1}H NMR (500 MHz, $CDCl_3$)
BC2
f1 (ppm)

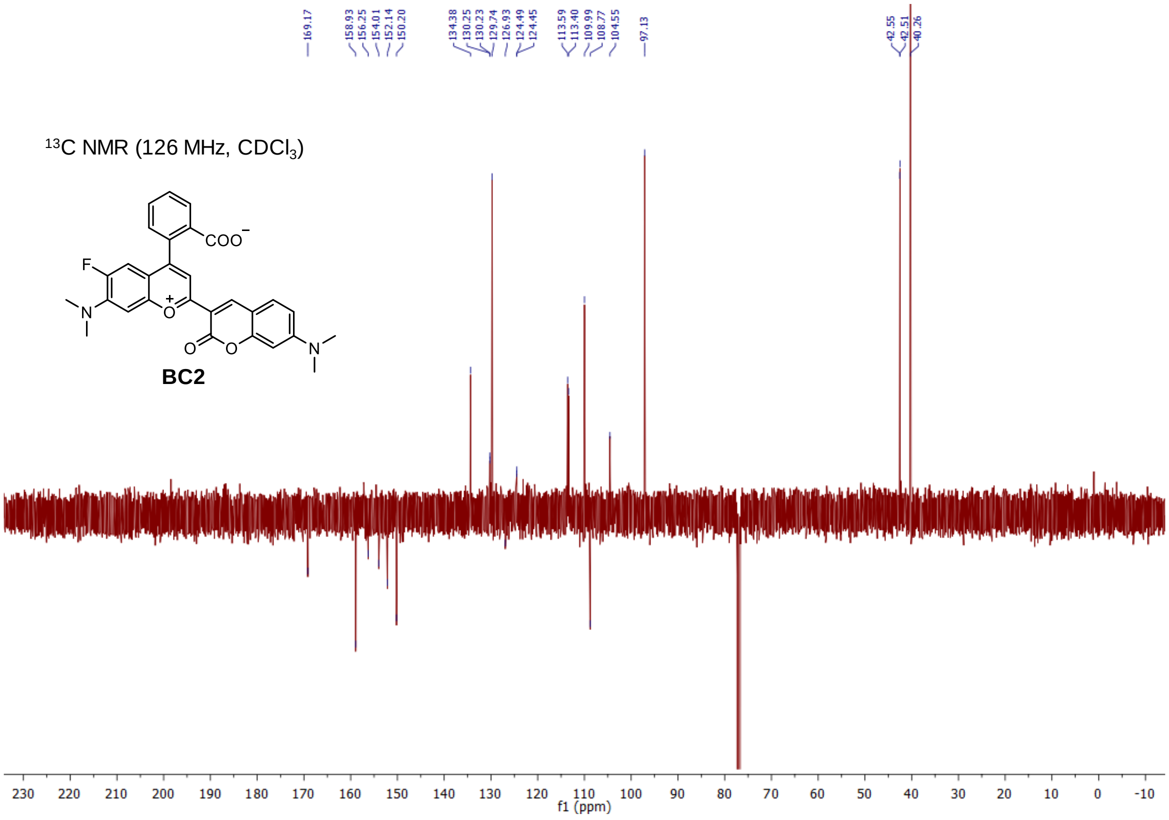

^{13}C NMR (126 MHz, $CDCl_3$)
BC2
f1 (ppm)

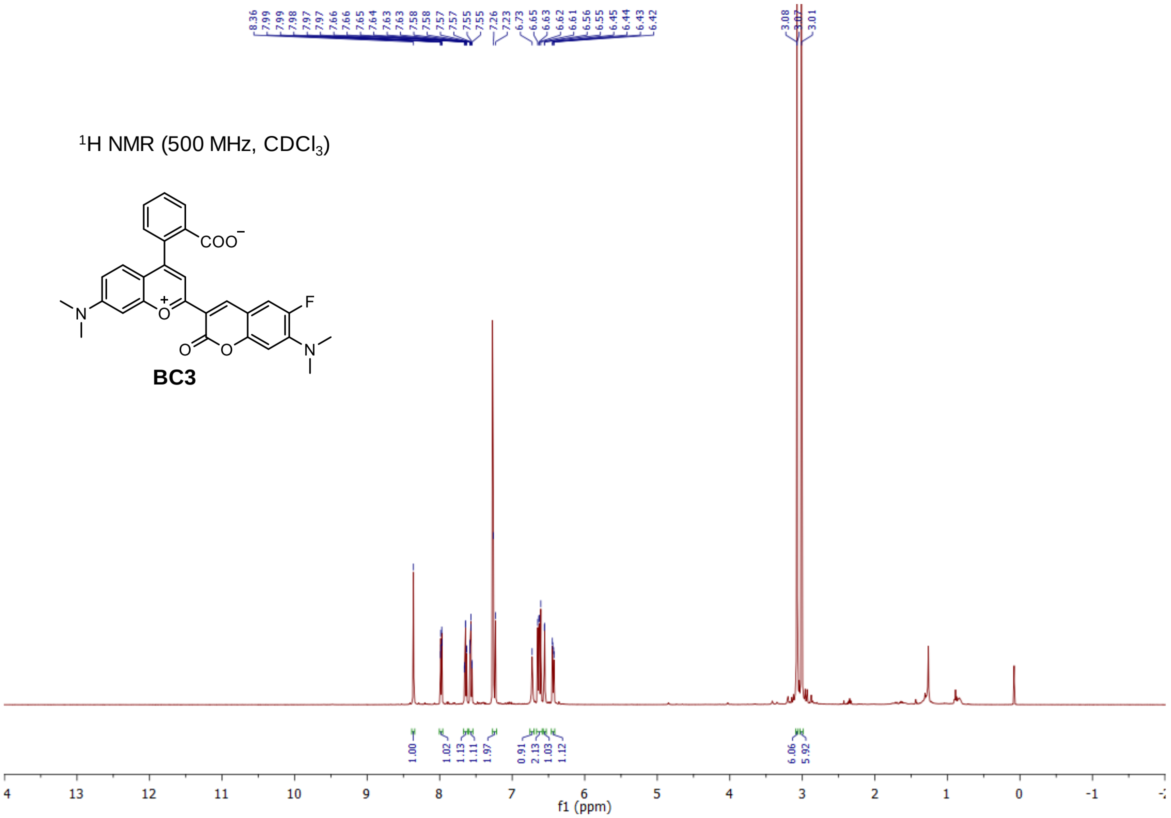
8.36 7.99 7.98 7.97 7.97 7.66 7.65 7.64 7.63 7.63 7.58 7.58 7.57 7.57 7.55 7.55 7.26 7.23 6.73 6.65 6.63 6.62 6.61 6.56 6.55 6.45 6.44 6.43 6.42
3.08 3.02 3.01
^{1}H NMR (500 MHz, $CDCl_3$)
COO$^-$
F
N
O
BC3
1.00 1.02 1.13 1.11 1.97 0.91 2.13 1.03 1.12 6.06 5.92
f1 (ppm)

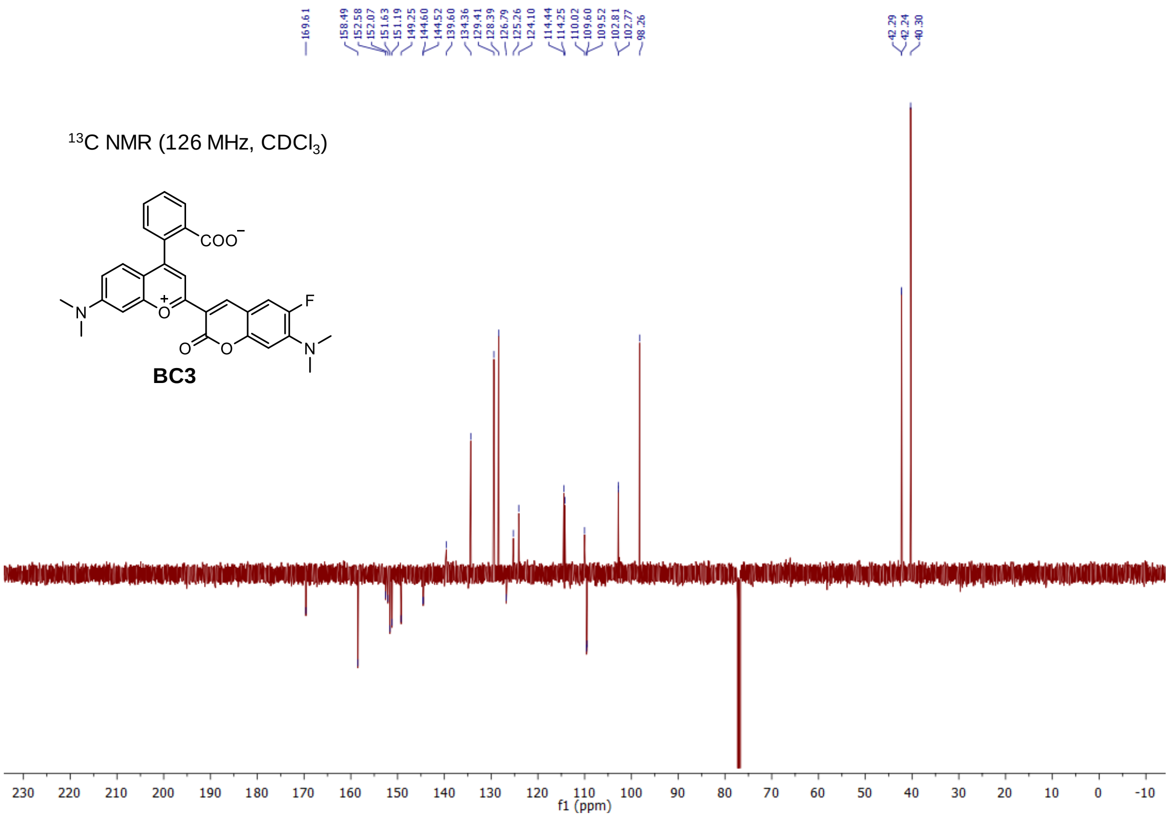
169.61 158.49 152.58 152.07 151.63 151.19 149.25 144.60 144.52 139.60 134.36 129.41 128.39 126.79 125.26 124.10 114.44 114.25 110.02 109.60 109.52 102.81 102.77 98.26
42.29 42.24 40.30
^{13}C NMR (126 MHz, $CDCl_3$)
COO$^-$
F
N
O
BC3
f1 (ppm)

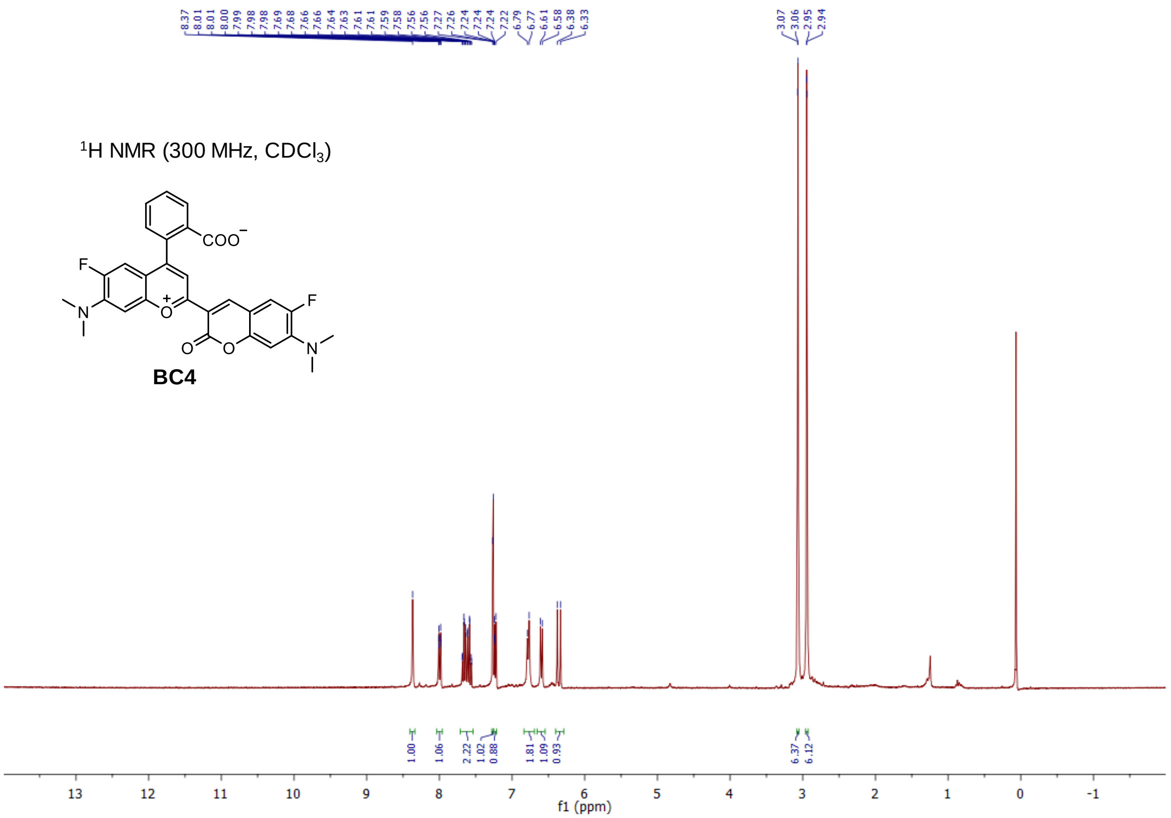
1H NMR (300 MHz, CDCl3)
COO−
F
N
O
O
F
N
O
BC4
f1 (ppm)

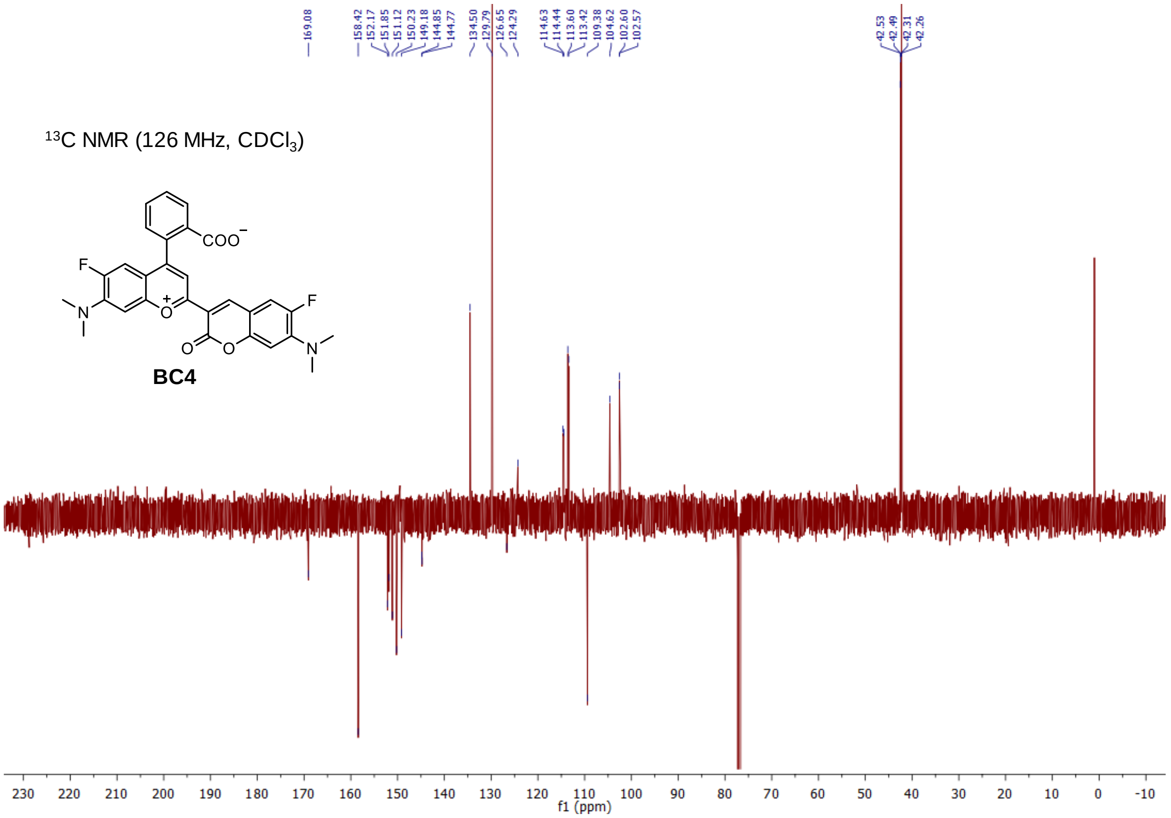
13C NMR (126 MHz, CDCl3)
COO−
F
N
O
O
F
N
O
BC4
f1 (ppm)

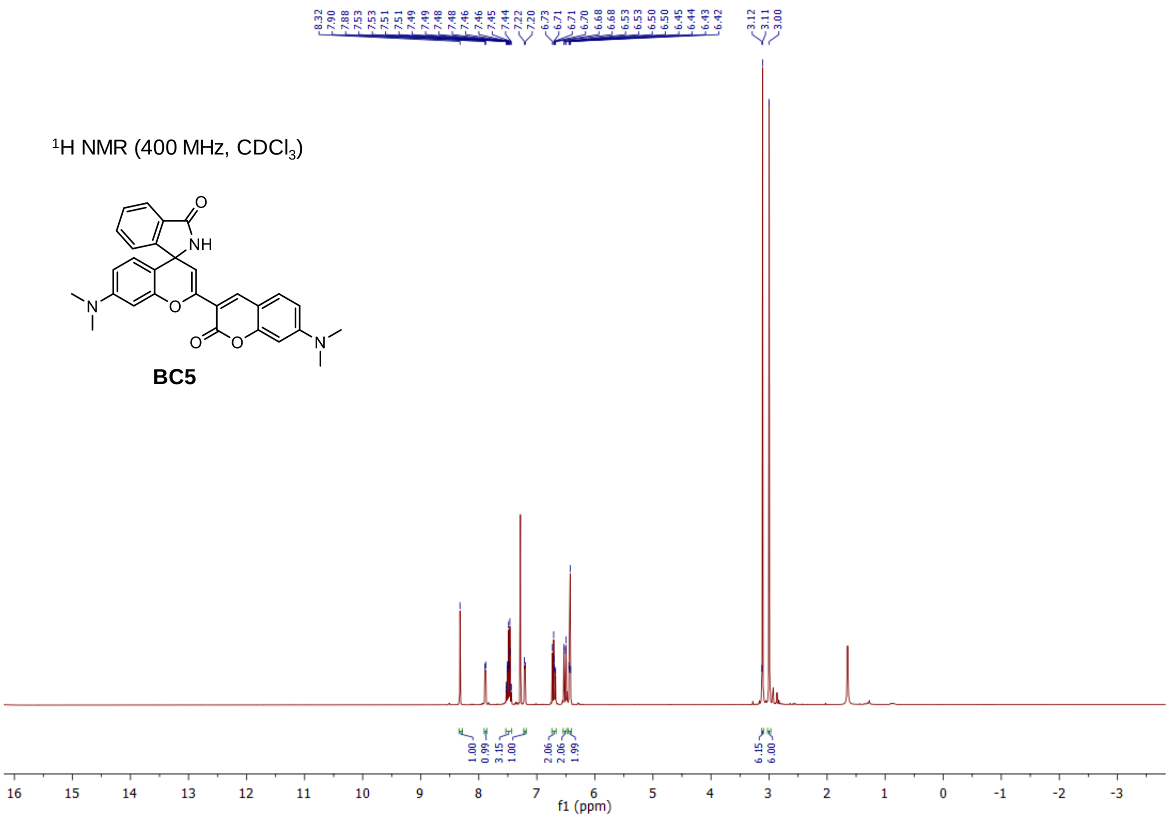
1H NMR (400 MHz, CDCl3)
BC5
f1 (ppm)

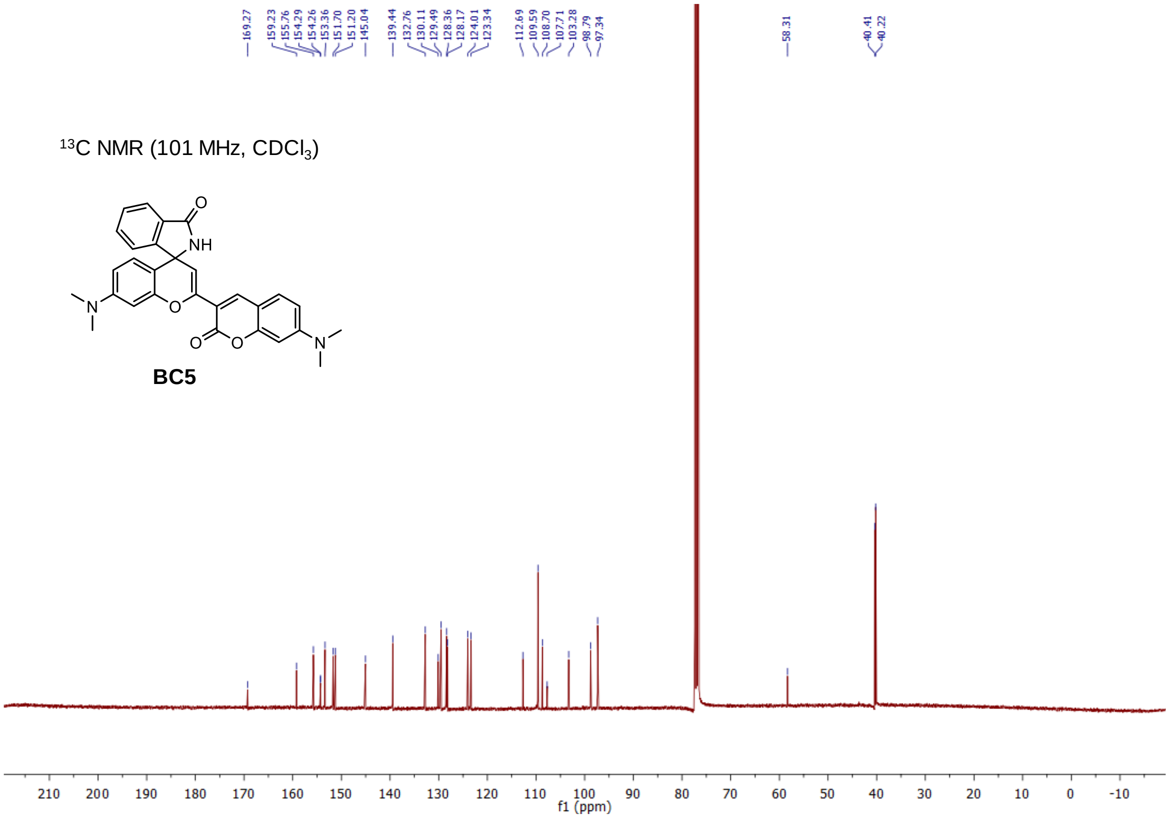
13C NMR (101 MHz, CDCl3)
BC5
f1 (ppm)

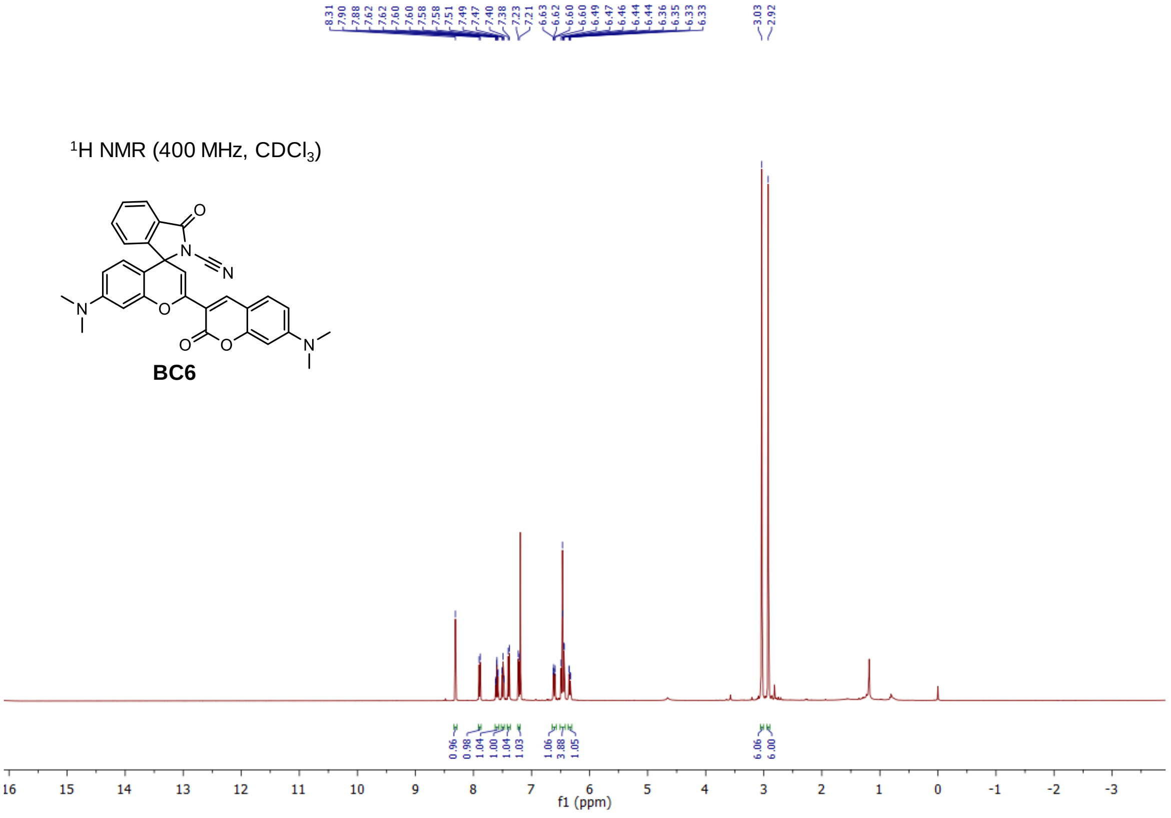
8.31 7.90 7.88 7.62 7.62 7.60 7.60 7.58 7.58 7.51 7.49 7.47 7.40 7.38 7.23 7.21 6.63 6.62 6.60 6.60 6.49 6.47 6.46 6.44 6.44 6.36 6.35 6.33 6.33
3.03 2.92
1H NMR (400 MHz, CDCl3)
BC6
0.96 0.98 1.04 1.00 1.04 1.03 1.06 3.88 1.05 6.06 6.00
f1 (ppm)

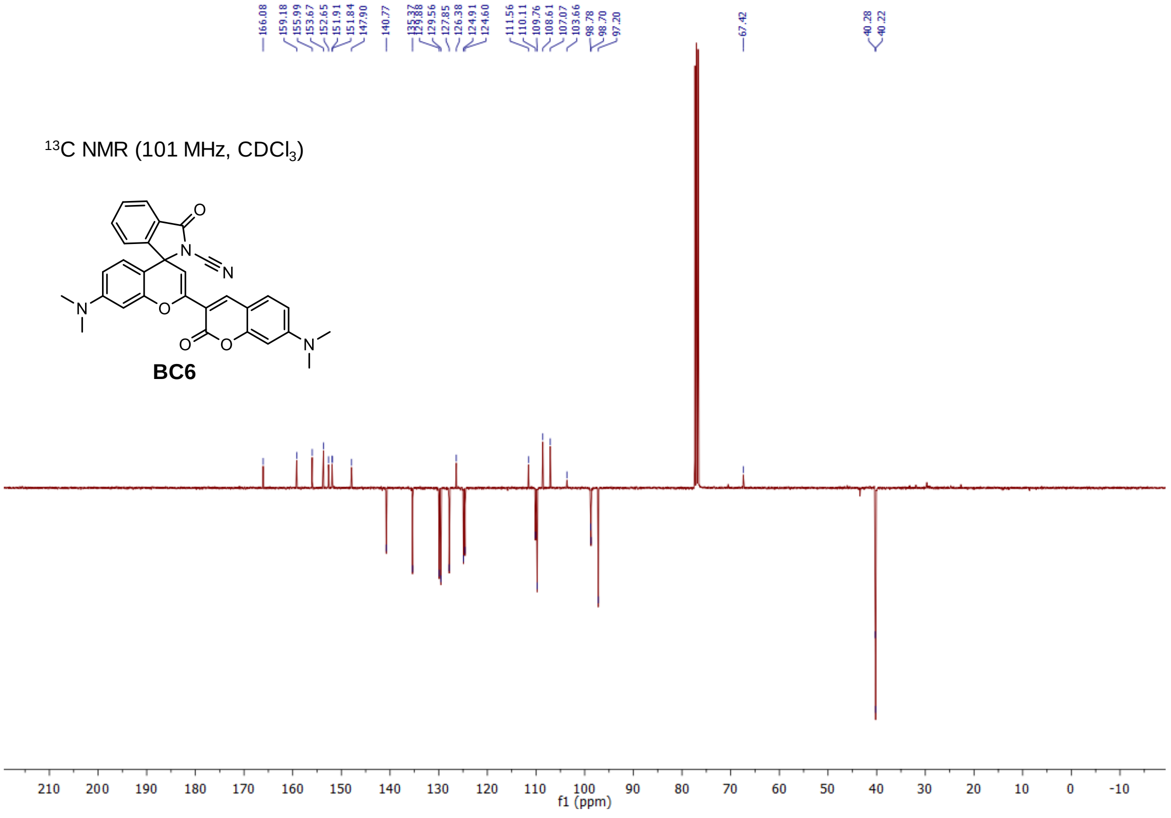
166.08 159.18 155.99 153.67 152.65 151.91 151.84 147.90 140.77 135.37 129.88 129.56 127.85 126.38 124.91 124.60 111.56 110.11 109.76 108.61 107.07 103.66 98.78 98.70 97.20 67.42 40.28 40.22
13C NMR (101 MHz, CDCl3)
BC6
f1 (ppm)

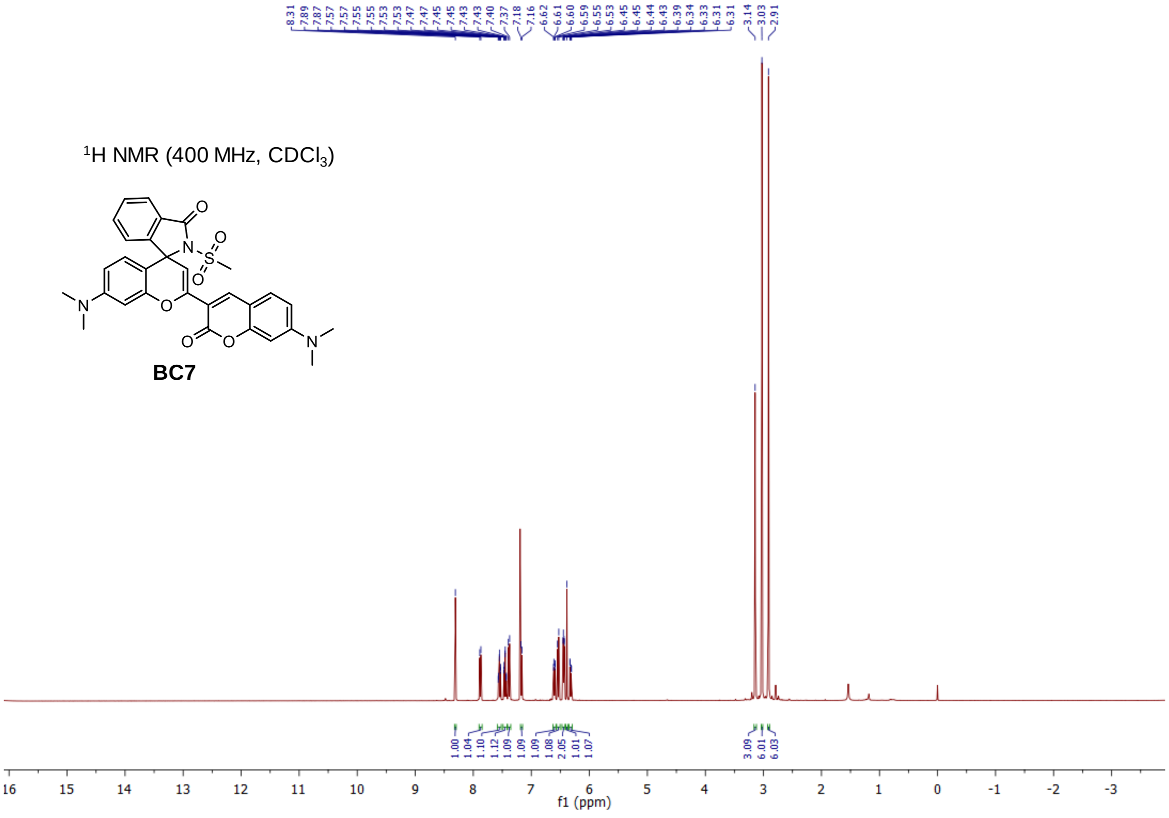

^{1}H NMR (400 MHz, $CDCl_3$)
BC7
f1 (ppm)

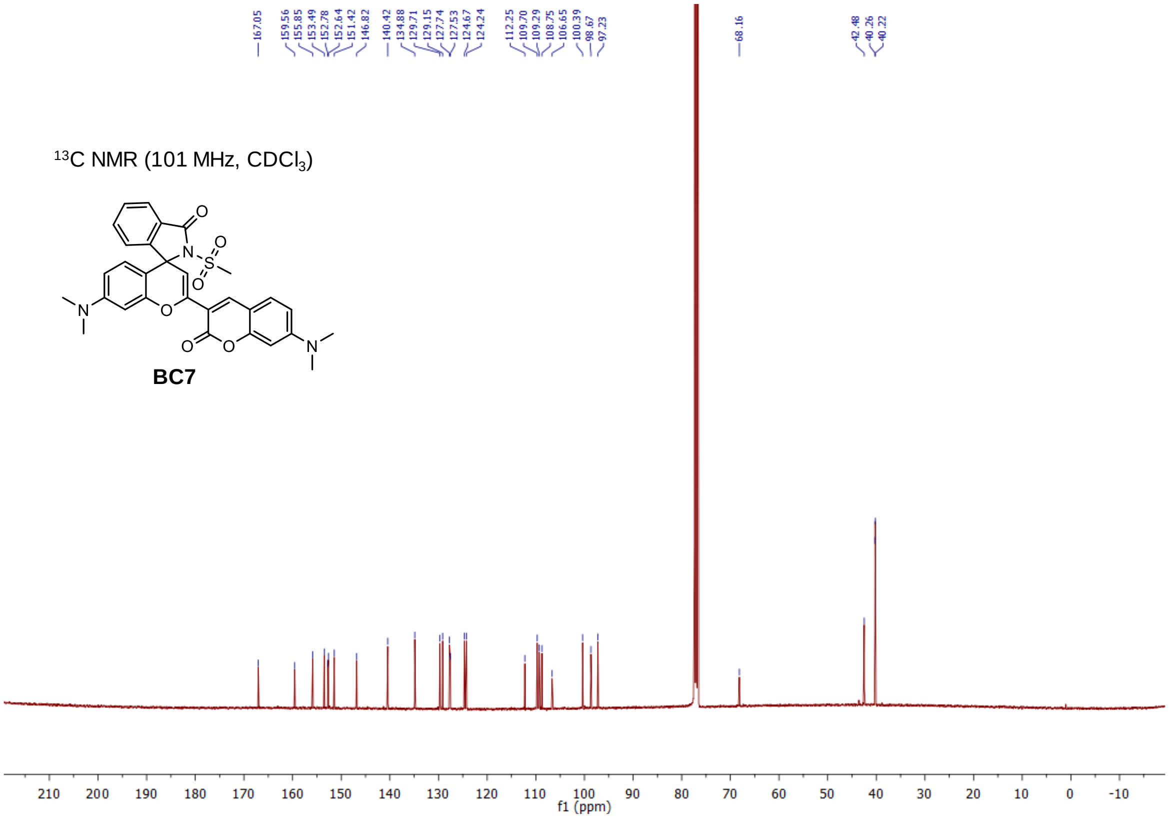

^{13}C NMR (101 MHz, $CDCl_3$)
BC7
f1 (ppm)

9. References

[1] F. Corpet, *Nucleic Acids Res* **1988**, *16*, 10881-10890.
[2] F. E. Critchfield, J. A. Gibson, J. L. Hall, *J Am Chem Soc* **1953**, *75*, 1991-1992.
[3] C. Wurth, M. Grabolle, J. Pauli, M. Spieles, U. Resch-Genger, *Nat Protoc* **2013**, *8*, 1535-1550.
[4] M. Sunbul, A. Jäschke, *Angew Chem Int Ed Engl* **2013**, *52*, 13401-13404.
